# Investigation of long non-coding RNAs in extracellular vesicles from low-volume blood serum specimens of colorectal cancer patients

**DOI:** 10.1007/s10238-024-01323-1

**Published:** 2024-04-03

**Authors:** Marie Boudna, Tana Machackova, Petra Vychytilova-Faltejskova, Karolina Trachtova, Renata Bartosova, Tina Catela Ivkovic, Dagmar Al Tukmachi, Robin Jugas, Lucie Pifkova, Jana Orlickova, Jan Kotoucek, Marketa Pavlikova, Milana Sachlova, Lucia Bohovicova, Teodor Stanek, Jana Halamkova, Igor Kiss, Tomas Grolich, Martin Svoboda, Zdenek Kala, Kamila Souckova, Ondrej Slaby

**Affiliations:** 1 Centre for Molecular Medicine, Central European Institute of Technology, Masaryk University, Kamenice 5, 625 00 Brno, Czech Republic; 2 Department of Biology, Faculty of Medicine, Masaryk University, Kamenice 5, 625 00 Brno, Czech Republic; 3 Department of Pharmacology and Toxicology, Veterinary Research Institute, Brno, Czech Republic; 4 Department of Gastroenterology and Digestive Endoscopy, Masaryk Memorial Cancer Institute, Brno, Czech Republic; 5 Department of Comprehensive Cancer Care, Faculty of Medicine, Masaryk Memorial Cancer Institute, Brno, Czech Republic; 6 Department of Surgical Oncology, Faculty of Medicine, Masaryk Memorial Cancer Institute, Brno, Czech Republic; 7 Department of Surgery, Faculty of Medicine, University Hospital Brno Bohunice, Masaryk University, Brno, Czech Republic

**Keywords:** lncRNAs, EVs, Colorectal cancer, Biomarker

## Abstract

Colorectal cancer (CRC) is the second most prevalent cancer type worldwide, which highlights the urgent need for non-invasive biomarkers for its early detection and improved prognosis. We aimed to investigate the patterns of long non-coding RNAs (lncRNAs) in small extracellular vesicles (sEVs) collected from low-volume blood serum specimens of CRC patients, focusing on their potential as diagnostic biomarkers. Our research comprised two phases: an initial exploratory phase involving RNA sequencing of sEVs from 76 CRC patients and 29 healthy controls, and a subsequent validation phase with a larger cohort of 159 CRC patients and 138 healthy controls. Techniques such as dynamic light scattering, transmission electron microscopy, and Western blotting were utilized for sEV characterization. Optimized protocol for sEV purification, RNA isolation and preamplification was applied to successfully sequence the RNA content of sEVs and validate the results by RT-qPCR. We successfully isolated sEVs from blood serum and prepared sequencing libraries from a low amount of RNA. High-throughput sequencing identified differential levels of 460 transcripts between CRC patients and healthy controls, including mRNAs, lncRNAs, and pseudogenes, with approximately 20% being lncRNAs, highlighting several tumor-specific lncRNAs that have not been associated with CRC development and progression. The validation phase confirmed the upregulation of three lncRNAs (*NALT1*, *AL096828*, and *LINC01637*) in blood serum of CRC patients. This study not only identified lncRNA profiles in a population of sEVs from low-volume blood serum specimens of CRC patients but also highlights the value of innovative techniques in biomolecular research, particularly for the detection and analysis of low-abundance biomolecules in clinical samples. The identification of specific lncRNAs associated with CRC provides a foundation for future research into their functional roles in cancer development and potential clinical applications.

**Supplementary Information:**

The online version contains supplementary material available at 10.1007/s10238-024-01323-1.

## Background

Colorectal cancer (CRC) accounts for an estimated 10% of all cancers diagnosed in men and women and is the second leading cause of cancer death worldwide [1]. Despite improvements in detection and treatment approaches, a significant number of patients with CRC face an unfavorable prognosis, which largely depends on the extent of disease at the time of diagnosis [2]. As CRC often develops through a gradual progression from adenoma to carcinoma [3], early diagnosis and resection of precancerous tissue would lead to an improved patient prognosis [4]. In this context, the availability of non-invasive biomarkers that can provide reliable and early detection of CRC is of high priority.

In recent years, extracellular vesicles (EVs) have emerged as potential reservoirs of clinically useful biomarkers that are present in all body fluids [5]. EVs represent a heterogeneous class of membranous vesicles, characterized by distinct biogenesis, size, biochemical composition, and cells of origin [6]. In relation to their size, small EVs (sEVs) typically measure 30–200 nm in diameter, in contrast to medium/large EVs that exceed this size range [7]. Secreted by a variety of cell types including cancer cells, EVs primarily function as mediators of cell-to-cell communication, facilitating intercellular crosstalk at both local and distant levels [8]. By transferring biomolecules such as proteins, lipids, and nucleic acids between cells, they reflect the various physiological and pathological states of the originating cells and influence the behavior of recipient cells [9]. Particularly in cancer pathogenesis, EVs play a crucial role by selectively packaging and transporting oncogenic cargos to target cells. This selective transfer facilitates processes such as the modulation of interactions within the tumor microenvironment, promotion of angiogenesis and development of metastasis, thereby emphasizing the involvement of EVs in tumor progression and metastatic dissemination [10]. With regard to the transfer of nucleic acid to recipient cells, lncRNAs carried by EVs have gained significant attention for their potential as biomarkers in early diagnosis. Considering the enhanced stability of EVs in the bloodstream [11], the detection of lncRNAs within these vesicles makes them highly promising candidates for non-invasive CRC detection.

Long ncRNAs are a group characterized by transcripts of at least 200 nucleotides in length that are not translated into protein [12]. Since lncRNAs are often tissue-specific and can serve as signaling molecules in intercellular communication, their quantification in EVs has been proposed as a non-invasive method for early detection of CRC [12, 13]. To date, more than thirty oncogenic lncRNAs involved in key signaling pathways related to the molecular pathogenesis of CRC have been identified, and the number is expected to increase [14]. For example, one of the described exosomal lncRNAs is CRNDE-h, whose elevated levels in CRC patients were significantly correlated with adverse prognosis, lymph node metastasis and the presence of distant metastases [15]. Given the growing body of research indicating that the content of EVs mirrors the biological state of their originating cells, and considering their production by cancer cells, we aimed to analyze sEV-derived lncRNAs as potential biomarkers for early disease diagnosis.

## Material and methods

### Study population and blood serum collection

Human blood sera were obtained with informed consent, and studies were approved (ID 2018/1671/MOU) by the Ethical Committee of Masaryk Memorial Cancer Institute (MMCI) in Brno, Czech Republic. Informed consent was obtained from all individuals included in this study. Blood sera were collected from patients with histopathologically verified CRC prior to surgery, or from participants undergoing cancer preventive screening at the MMCI. These participants had negative results for the Fecal Occult Blood Test, the markers CEA and CA19-9, and abdominal ultrasound. After the collection, samples were stored in a biobank at − 80 °C. A cohort of 76 patients and 29 healthy controls was enrolled for the exploratory phase of this study, and 159 CRC patients and 138 healthy controls were included in the subsequent validation phase. Clinical and pathological characteristics of the study population are summarized in Table 1.

**Table 1:** Clinical and pathological characteristics of CRC patients and healthy controls

	Exploratory cohort (n = 105)		*P* value	Validation cohort (n = 297)		*P* value
	CRC patients n = 76	Healthy controls n = 29		CRC patients n = 159	Healthy controls n = 138	
Age (yr)						
≤ 55	12	6	0.474	29	35	0.137
> 55	64	23		130	103	
Gender						
Male	42	15	0.745	105	72	0.015
Female	34	14		54	66	
Diagnosis (ICD) and disease stage						
C18		–			–	
Stage I	12			11		
Stage II	12			22		
Stage III	10			31		
Stage IV	4			19		
C19		–			–	
Stage I	2			1		
Stage II	2			12		
Stage III	4			10		
Stage IV	3			8		
C20		–			–	
Stage I	5			18		
Stage II	9			15		
Stage III	7			6		
Stage IV	6			6		

*ICD* International Classification of Diseases, *C18* malignant neoplasm of the colon, *C19* malignant neoplasm of the rectosigmoid, *C20* malignant neoplasm of the rectum. *P* value for Pearson’s χ^2^ test

### Purification of sEVs from human blood serum

Blood was left in serum separator tubes at room temperature for 30 min in an upright position until a clot formed; then, it was centrifuged at 2200× g for 15 min at 4 °C. The serum supernatant was transferred in Eppendorf tubes and stored at − 80 °C. After thawing on ice, 250 μl of blood serum was processed by differential centrifugation at 4 °C. Serum was spun at 1500× *g* for 10 min to sediment cell organelles and debris. The supernatant was then centrifuged at 10,000× *g* for 20 min to remove larger particles and microvesicles. Isolation of sEVs was achieved by size exclusion chromatography (SEC) as previously described [16]. In brief, 150 μl of purified serum was loaded onto a qEVsingle 35 nm (iZON Science Ltd., UK) column that was equilibrated with PBS. After discarding the first 800 μl of void volume, the fraction containing sEVs was eluted with 500 μl of PBS. For enzymatic treatment of the sEV fraction, 10 µl of proteinase K (20 mg/ml) and 5 μl of RNase Cocktail Enzyme Mix (20,000 U/ml) were added individually. The activity of RNase was inhibited by addition of 4 µl of SUPERase In RNase Inhibitor (20 U/μl) (all Invitrogen, USA).

### Negative stain transmission electron microscopy

Four μl of sEV fraction was applied onto copper grids coated with a thin carbon layer, which had been freshly cleaned with plasma. This was followed by staining with 2% uranyl acetate, allowing 30 s for sample incubation and 1 min for the staining process. The grids were loaded into Talos F200C (ThermoScientific) transmission electron microscope for imaging, and the microscope was operated at 200 kV. The EV images were collected on a Ceta-16 M CMOS camera at the 36,000× nominal magnification with an underfocus of 2–4 μm.

### Multi-angle dynamic light scattering

To determine the particle size and concentration, 50 µl of sEV fraction was placed in low-volume quartz batch cuvette ZEN2112 (Malvern Panalytical Ltd, UK) and measured using Multi-angle dynamic light scattering technique (MADLS), Zetasizer Ultra (Malvern Panalytical Ltd, UK) at a constant temperature of 25 °C. The light scattering data were collected at three angles, 173°, 90°, and 13°, and evaluated using ZS Xplorer software version 2.50 (Malvern Panalytical Ltd, UK). The hydrodynamic diameter, polydispersity index, and concentration results are reported as mean value (*n* = 3) ± standard deviation.

### Western blot

EV sample was concentrated using Concentrator plus 5305 Vacuum Centrifuge (Eppendorf AG, Germany), and protein concentration was measured with Pierce BCA Protein Assay Kit (Thermo Scientific). Concentrated sEV preparations and lysate of HCT116 cells were lysed in Pierce Lane Marker Reducing Sample Buffer (Thermo Scientific), heated for 5 min at 95 °C, and subjected to electrophoresis using 10% SDS-PAGE. Proteins were transferred to an Immobilon-P PVDF Membrane (Merck Millipore) and the excess protein binding sites on the membrane were saturated with 5% bovine serum albumin blocking buffer (1 × TBS, 0.1% Tween-20) for 1 h. The membrane was incubated overnight at 4 °C with primary antibody. The following antibodies were used: anti-CD81 (1:250, mouse, catalogue number sc166029), anti-CD63 (1:300, mouse, sc5275) from Santa Cruz Biotechnology, anti-Alix (1:20000, rabbit, ab186429) from Abcam, anti-TSG101 (1:200, mouse, 612696) from BD Biosciences, and anti-Calnexin (1:1000, rabbit, 2679) from Cell Signaling. After incubation, the membrane was washed three times with 5% TBS-Tween and then, incubated with peroxidase-labelled secondary antibody (Santa Cruz Biotechnology) for one hour. After three washes, immobilized proteins were detected utilizing Clarity Western ECL Substrate (Bio-Rad) and the UVITEC chemiluminescence imager (UVITEC Cambridge, UK).

### Isolation of RNA from sEVs

Small EVs were disrupted by adding an equal volume of lysis buffer from Monarch Total RNA Miniprep Kit (New England Biolabs, USA) and vortexed shortly. An equal volume of ethanol (≥ 95%) was pipetted to the lysed sample and mixed thoroughly. The remaining steps were done according to the manufacturer’s protocol including DNase treatment provided with the kit. RNA was eluted with 50 μl of nuclease-free water.

### Library preparation and RNA sequencing

Isolated and undiluted RNA was concentrated from 50 to 5 μl using Concentrator plus 5305 Vacuum Centrifuge (Eppendorf AG, Germany). RNA was not further fragmented or subjected to any kind of selection. Sequencing libraries were prepared using NEBNext Ultra II Directional RNA Library Prep Kit for Illumina (New England BioLabs, USA) according to manufacturer’s recommendations with two exceptions; due to low RNA input, libraries were subjected to 18 cycles of amplification, and NEBNext Adaptor was diluted 200× before ligation. Libraries were individually barcoded with NEBNext Multiplex Oligos for Illumina (New England Biolabs, USA). Library concentration and quality were assessed fluorometrically using Qubit 4.0 Fluorometer and Qubit HS DNA Assay Kit (Thermo Fisher Scientific) and electrophoretically using Agilent 2200 TapeStation System and High Sensitivity D1000 ScreenTape (Agilent Technologies). Each library was diluted to a final concentration of 4 nM and pooled equimolar prior to clustering. RNA sequencing (single read, 75 cycles) was performed using the NextSeq 500/550 High Output Kit v2 and the NextSeq 500/550 instrument (Illumina, USA).

### Bioinformatic analysis of sequencing data

Raw sequencing images from the Illumina NextSeq 550 were demultiplexed and converted to FASTQ format using bcl2fastq (version 2.20.0). Generated reads were single-ended and 100 nucleotides in length. Quality of FASTQ data was checked with FastQC (v0.11.9) and MultiQC (v1.8). Adapters and low-quality ends were trimmed using Trimmomatic (v0.39) and reads shorter than 35 nt were discarded. Pre-processed reads were mapped to the reference human genome (Gencode GRCh38, release 37) with STAR. The quality of mapping was evaluated with tools RSeQC (v2.6.4) and Picard (v2.22.3), and rRNA content was checked with FastQ Screen (v0.14.0). Gene quantification was performed on uniquely mapped reads only with featureCounts (v2.0.1). Differential level analysis was carried out in R (version 4.0.3) with DESeq2 package (v1.28.1).

### RT-qPCR analysis

RNA samples purified from serum sEVs of CRC patients and healthy controls were pooled into individual groups, each consisting of three samples, and concentrated to 6 µl by Concentrator Plus 5305 Vacuum Centrifuge (Eppendorf AG, Germany). Next, 5 μl of RNA was converted to cDNA using High Capacity cDNA Reverse Transcription kit (Applied Biosystems) and preamplified for 14 cycles with TaqMan PreAmp Master Mix (Applied Biosystems) according to the manufacturer’s instructions. Briefly, 2.5 µl cDNA was added to a reaction mix containing 5 µl of TaqMan PreAmp Master Mix and 2.5 µl of primer pool (200 nM). Preamplification was performed in a thermal cycler set for 1 cycle at 95 °C for 10 min, followed by 14 cycles of amplification at 95 °C for 15 s and 60 °C for 4 min, before enzyme inactivation at 99 °C for 10 min. Amplified samples were diluted 1:20 in 1 × TE buffer, and a 2.5 µl aliquot was used for qPCR reaction in a total reaction volume of 10 µl. PowerUp SYBR Green Master Mix (Applied Biosystems) was utilized in the reaction mix following manufacturer’s instructions. Primer sequences were designed with PrimerQuest™ Tool and synthesized by Integrated DNA Technologies (Supplementary Table S1). For the measurement of reference genes, preamplification of cDNA was also performed for 14 cycles with the following changes in the reaction setup: 1.5 µl of cDNA was added to a reaction mix containing 5 µl of TaqMan PreAmp Master Mix, 2.5 µl of pooled assays (0.2×), and 1 µl of nuclease-free water. Amplified samples were diluted 1:20 in 1 × TE buffer, and 2.5 µl aliquot was used for a qPCR reaction containing 5 µl of TaqMan Gene Expression Master mix (Thermo Fisher Scientific, USA), 0.5 µl of TaqMan Gene Expression assay (20×), and 2 µl of nuclease-free water. Ct values of all genes including reference genes were detected on QuantStudio 12 K Flex (Applied Biosystems).

### Statistical analysis of RT-qPCR results

The threshold cycle (CT) expression value was set to 0.2. Relative abundance of lncRNA genes detected in sEVs was calculated with 2^−ΔCt^ formula using a combination of *GAPDH* and *ACTB* for data normalization. The selection of reference genes was based on the algorithms geNorm and NormFinder [17, 18]. LncRNAs with a Ct value above 35 were considered undetectable (Ct = 40). Comparison of lncRNA levels between healthy controls and CRC patients was made using the nonparametric Mann–Whitney test, and the Kruskal–Wallis test was performed for comparisons across multiple groups (tumor localization, disease stage) in GraphPad Prism software (version 8.0). Statistical significance was established at *P* < 0.05.

## Results

### Isolation and characterization of sEVs purified from blood serum

**Fig. 1:**
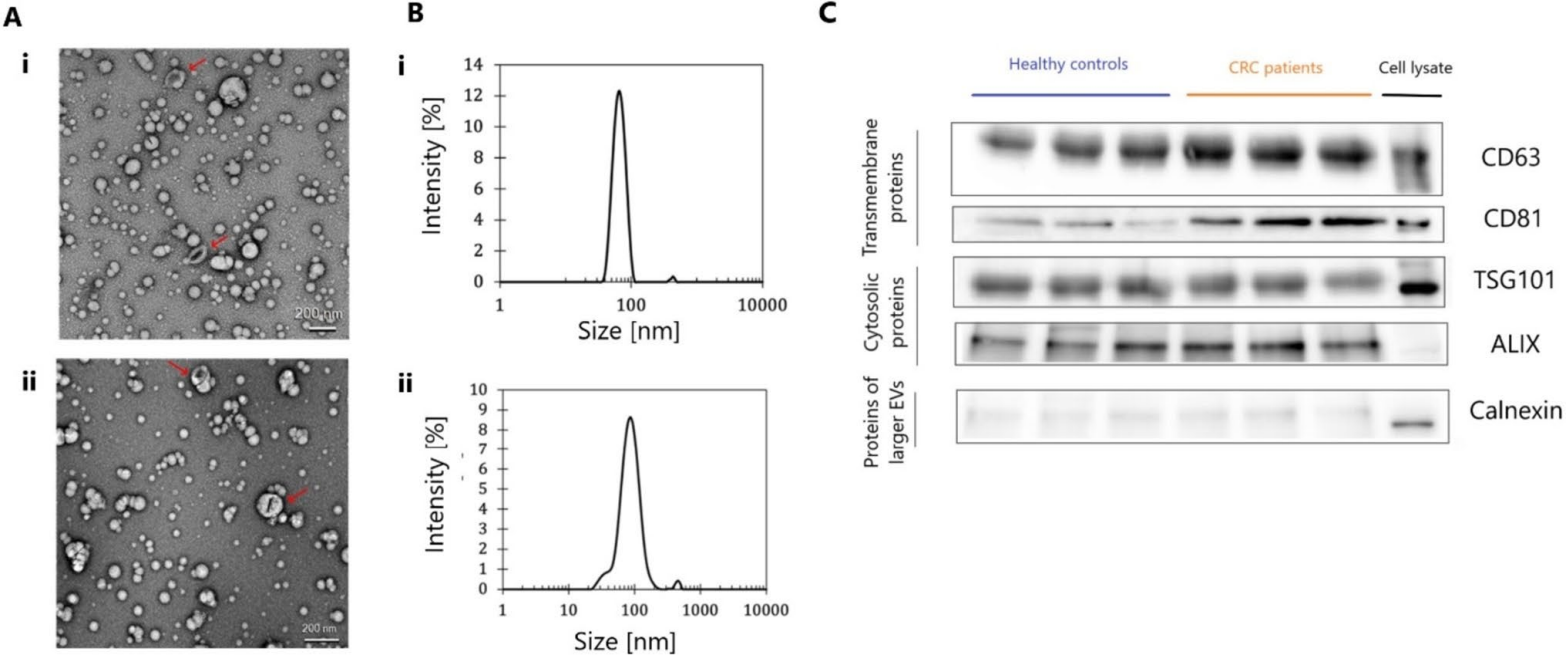
Characterization of isolated sEVs. **A** Representative transmission electron microscopy images of serum-derived sEVs isolated using size exclusion chromatography (scale bar, 200 nm) from CRC patient (**i**) and healthy control samples (**ii**). **B** Representative graphs by DLS analysis indicating concentration and size distribution of isolated particles from CRC patient (**i**) and healthy control samples (**ii**). **C** Representative Western blot images showing enrichment of EV markers CD63, CD81, TSG101, and Alix with a low level of endoplasmic reticulum proteins (calnexin) in serum-derived sEVs

To characterize isolated sEVs from blood serum of CRC patients and healthy controls, we utilized transmission electron microscopy (TEM) and dynamic light scattering (DLS) for particle size and concentration. TEM and DLS analysis were performed on a set of 40 samples (20 healthy controls and 20 CRC patients). In both types of specimens, TEM images (Fig. 1A) revealed particles that corresponded to the size of sEVs and had a characteristic cup shape as described in the literature [19, 20]. The presence of sEVs was also confirmed by the results of DLS analysis. In Fig. 1B, a visible peak in the region of about 50–150 nm corresponds to the size of these vesicles. The marginal peaks visible in both types of samples in the region of about 500 nm indicate the presence of particles of a larger size. In terms of an average particle size and polydispersity value index (PdI), the samples of healthy controls and CRC patients were comparable. The mean particle sizes were 82 ± 5 nm in healthy controls and 90 ± 7 nm in patient samples. Regarding PdI values, DLS measured 0.213 ± 0.063 in controls and 0.223 ± 0.084 in patient samples, which indicates that samples were purified. Slightly higher particle concentration was detected in the CRC patient samples, where it was determined to be 2.3 × 10^13^ ± 1.2 × 10^13^ particles per ml. In samples from healthy controls, the particle number was lower; the mean concentration was 2.5 × 10^12^ ± 8.1 × 10^11^ per ml. The protein content of purified sEVs was determined by Western blot in three patients and three healthy controls. The presence of characteristic markers of EVs such as CD63, CD81, TSG101 and Alix was confirmed in both CRC and healthy control samples. For CD81, a stronger signal was observed in all patient samples when compared to healthy controls. This could indicate an increased number of smaller vesicles in CRC patients, which is in agreement with the results of DLS analysis. However, this trend was not detected for the other protein markers. The detection of calnexin signal suggested the presence of endoplasmic reticulum proteins, however, in comparison with the signal of cell lysate it was very weak, indicating sufficiently purified population of sEVs.

### RNA sequencing of sEVs purified from blood serum

**Fig. 2:**
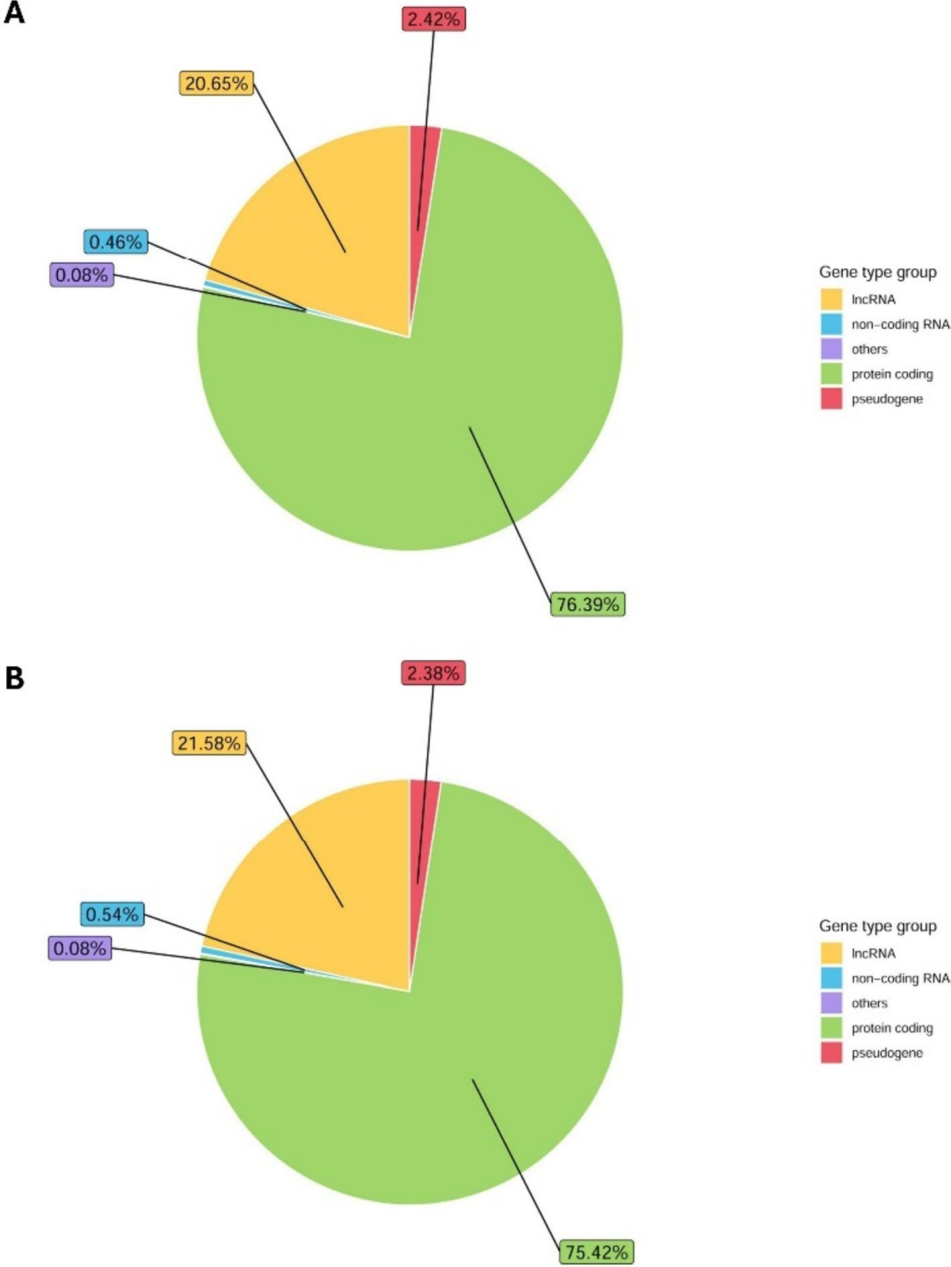
Gene biotypes detected by RNA sequencing of RNA content of sEVs from low-volume blood serum. **A** Percentage of counts assigned to gene types in CRC patients. **B** Percentage of counts assigned to gene types in healthy controls

**Fig. 3:**
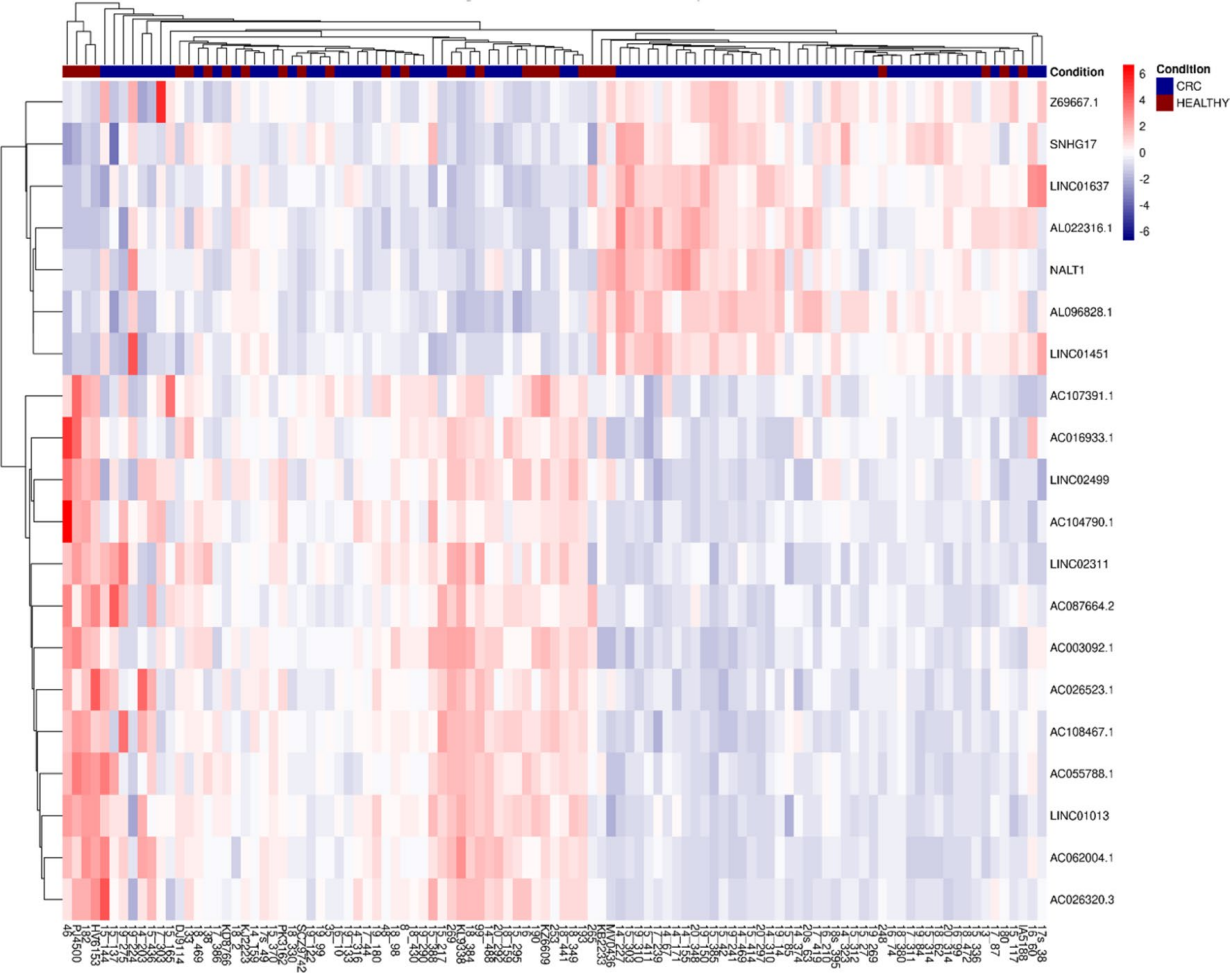
Heatmap clustering CRC patients and healthy control groups based on the 20 most differentially abundant lncRNAs measured in sEVs from blood serum. The blue-red scale represents the fold change of differentially abundant lncRNAs

The exploratory cohort included peripheral blood samples from 76 patients and 29 healthy controls. Utilizing a minimal starting material of 150 µl of blood serum for sEV isolation, we successfully prepared sequencing libraries from RNA purified from sEVs, despite the RNA concentration being below the detection level. Our RNA sequencing data revealed that 76% of sEV content comprised coding RNA and most of the non-coding RNA fraction was lncRNAs (21%). The overview of gene biotypes detected by RNA sequencing is shown in Fig. 2. Overall, we identified 460 genes (including protein coding genes, lncRNAs, and pseudogenes) that had different levels between CRC patients and healthy controls (*P* < 0.01; log2FC > 0.3 or <  − 0.3; normalized reads > 50). Interestingly, 379 out of 460 dysregulated genes had higher levels in CRC patients, while only 81 genes were detected with higher abundance in healthy controls. Approximately 80% of dysregulated genes were protein coding, and 20% were lncRNAs. Shifting focus to these lncRNAs, the heatmap in Fig. 3 shows the 20 most significantly dysregulated lncRNAs, of which 7 lncRNAs had higher levels, while 13 lncRNAs were detected at reduced levels in CRC patients compared to healthy controls. Table 2 presents lncRNAs with significantly different levels in sEVs from CRC patients compared to healthy controls. Based on Transcript Support Level values from the Ensembl database, 14 lncRNAs from Table 2 were selected for initial expression analysis in a smaller cohort of 20 CRC patients and 20 healthy controls. During this preliminary analysis, *LINC01451*, *AC026523.1*, *AC087664.2* were found to be not expressed in a significant majority of the samples, with over 90% showing a Ct value greater than 35, indicating negligible expression (data not shown). Consequently, these three lncRNAs were excluded from the subsequent validation.

**Table 2:** LncRNAs with significantly different levels in sEVs purified from blood serum specimens of CRC patients and healthy controls in the exploratory phase of the study

Gene ID	Gene name	Average number of normalized reads	Fold change (log2)	*P* value
ENSG00000251310.1	**AC107391.1**	**70.8**	** − 0.470**	**3.24 × 10**^**−5**^
ENSG00000251471.1	**AC016933.1**	**138.9**	** − 0.320**	**5.74 × 10**^**−5**^
ENSG00000250436.1	**LINC02499**	**143.3**	** − 0.327**	**8.42 × 10**^**−5**^
ENSG00000286937.1	**AC055788.1**	**275.5**	** − 0.322**	**1.03 × 10**^**−4**^
ENSG00000259582.4	AC026523.1	89.6	** − **0.427	1.47 × 10^−4^
ENSG00000258718.3	**LINC02311**	**72.4**	** − 0.413**	**2.52 × 10**^**−4**^
ENSG00000237476.1	**LINC01637**	**100.4**	**0.395**	**2.68 × 10**^**−4**^
ENSG00000286901.1	Z69667.1	53.8	0.444	3.12 × 10^−4^
ENSG00000228495.2	**LINC01013**	**195.6**	** − 0.332**	**4.61 × 10**^**−4**^
ENSG00000237886.1	**NALT1**	**58.7**	**0.587**	**4.79 × 10**^**−4**^
ENSG00000196756.13	SNHG17	159.0	0.317	6.51 × 10^−4^
ENSG00000285998.1	**AC104790.1**	**62.8**	** − 0.454**	**6.92 × 10**^**−4**^
ENSG00000253879.2	AC087664.2	78.1	− 0.410	6.96 × 10^−4^
ENSG00000236453.5	AC003092.1	246.8	− 0.313	7.66 × 10^−4^
ENSG00000248744.1	**AC108467.1**	**191.8**	** − 0.328**	**8.48 × 10**^**−4**^
ENSG00000230107.1	AL022316.1	109.7	0.382	1.05 × 10^−3^
ENSG00000279141.3	LINC01451	71.0	0.483	1.19 × 10^−3^
ENSG00000231977.1	**AL096828.1**	**189.2**	**0.349**	**1.25 × 10**^**−3**^
ENSG00000254366.7	AC062004.1	625.2	− 0.330	1.79 × 10^−3^
ENSG00000287178.1	AC026320.3	210.6	− 0.328	2.00 × 10^−3^

LncRNAs in bold were selected for the validation phase of the study

### Validation of selected lncRNAs by RT-qPCR

**Fig. 4:**
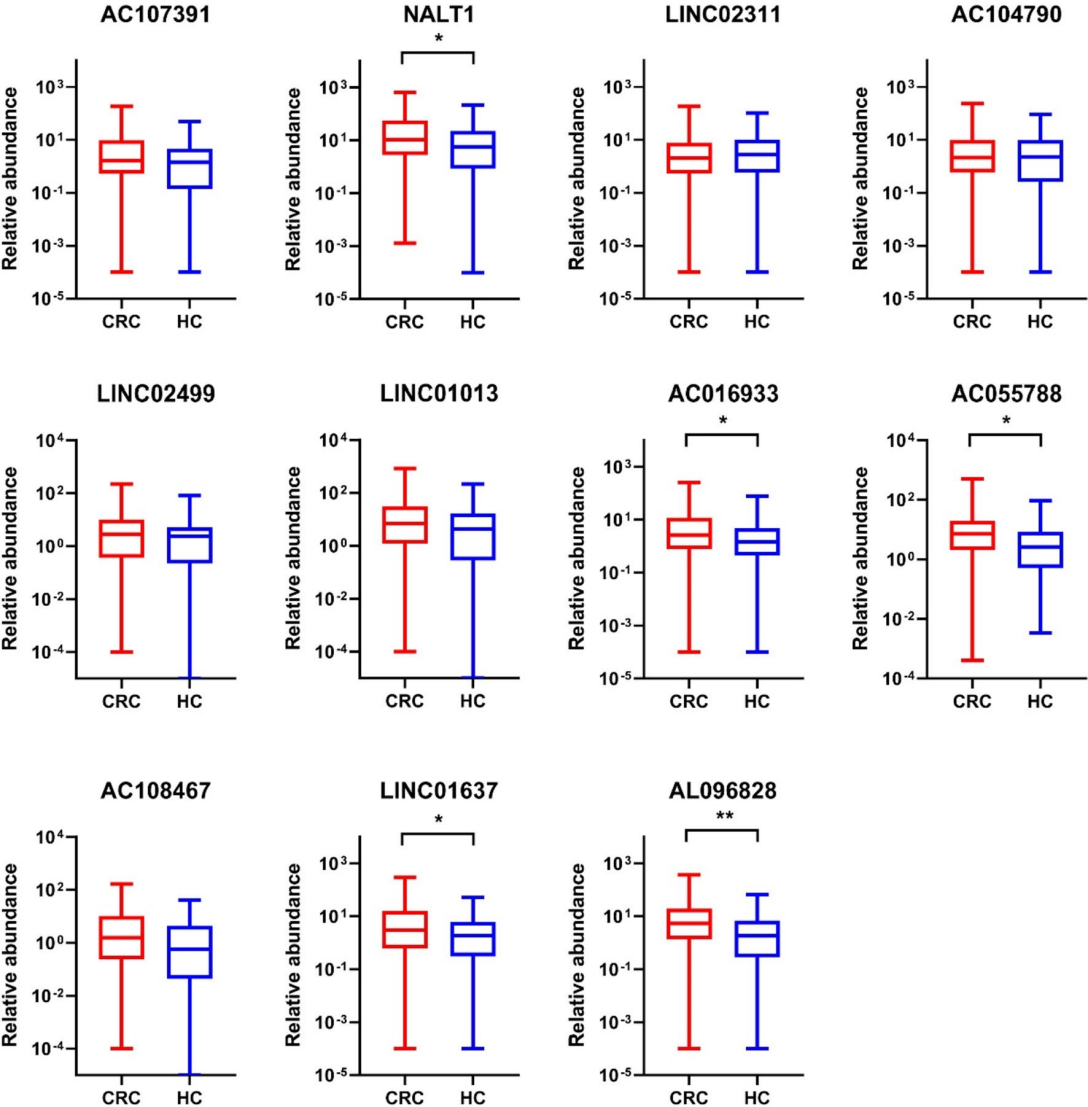
Box plots showing the levels of lncRNAs in sEVs of CRC patients and healthy controls. *P < 0.05; **P < 0.01

The validation phase therefore focused on the remaining 11 lncRNAs, which demonstrated differential levels suitable for further investigation. These 11 lncRNAs, highlighted in bold in Table 2, were analyzed by RT-qPCR on an independent set of 53 pools of CRC patients and 46 pools of healthy controls. Due to low RNA concentration, three RNA samples were combined in sample pools according to gender, age, and for patient samples, also by tumor localization and clinical stage (Supplementary Table S2). The obtained data were normalized using a combination of *GAPDH* and *ACTB* as reference genes. First, we compared the levels of lncRNAs in healthy controls and CRC patients using the nonparametric Mann–Whitney test with the significance level set at *P* < 0.05. Figure 4 shows graphs for the relative abundance of tested lncRNAs. Of the 11 lncRNAs, five genes showed significantly different levels between CRC patients and healthy controls. An overlap with RNA sequencing results was confirmed for three lncRNAs (*AL096828*, *LINC01637* and *NALT1*), which had elevated levels in CRC patients. Two lncRNAs (*AC016933*, *AC055788*) were also significantly increased in the serum of patients; however, this is in contrast with RNA sequencing results, which showed a significant decrease.

Relative abundance of selected lncRNAs was correlated with various locations of tumors, namely malignant neoplasm of the colon (C18), the rectosigmoid (C19), or the rectum (C20). The analysis was performed using Kruskal–Wallis test at a significance level of *P* < 0.05 (Supplementary Fig. S1). The pools with mixed tumor locations were excluded from the analysis. No significant correlation with tumor localization was confirmed for tested lncRNAs. However, for all lncRNAs, the lowest median value was observed for C20 diagnosis, and despite no statistically significant difference, we observed the trend of decreased abundance in rectal tumor (C20) compared to colon (C18).

### Correlation of lncRNA abundance in patient sEVs with CRC staging

**Fig. 5:**
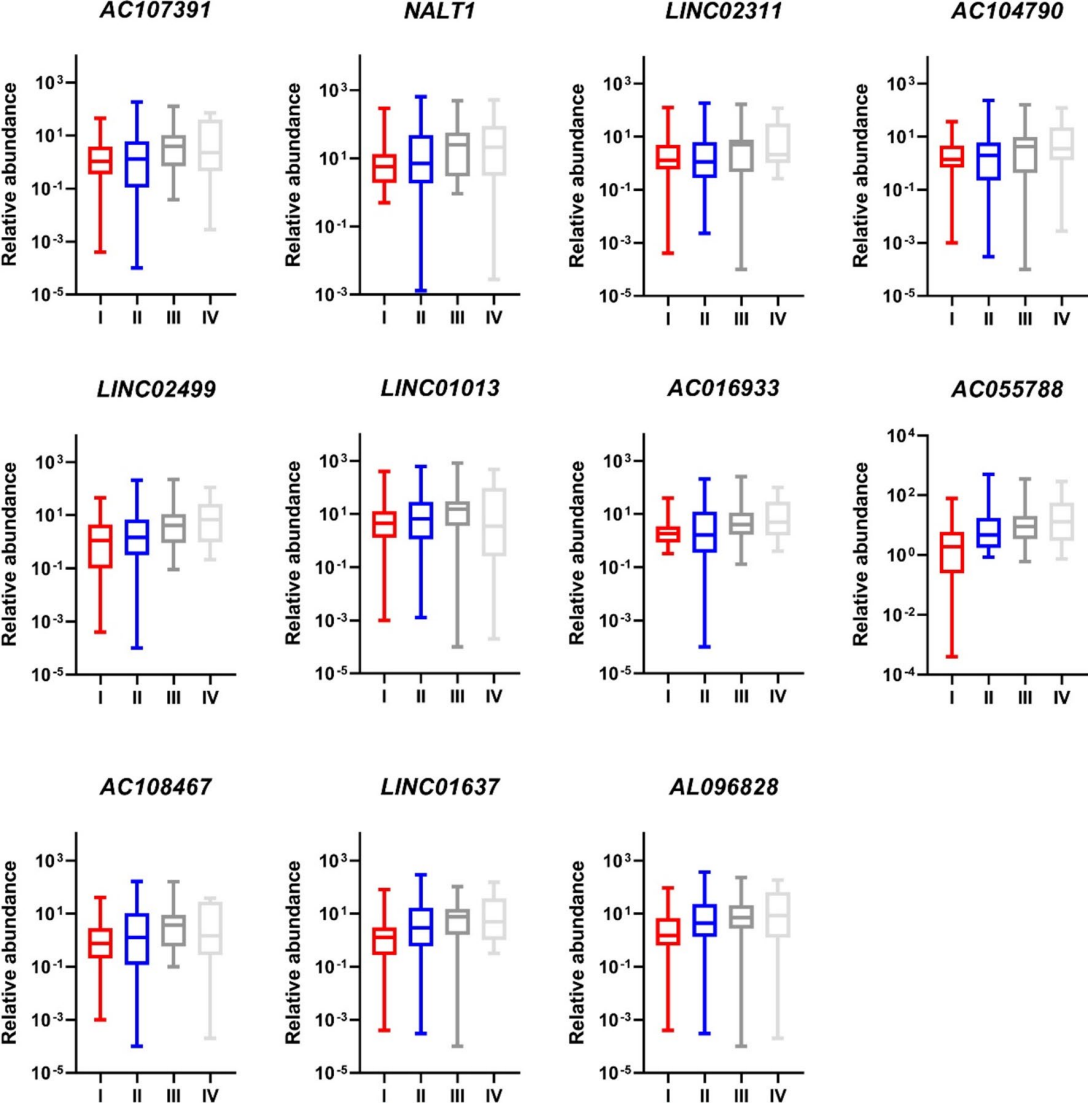
Box plots showing the levels of lncRNAs in sEVs of CRC patients with different clinical stages. The correlation was borderline significant for *AC055788* (*P* = 0.052). For other lncRNAs, there was a trend of increasing abundance with more advanced stages of colorectal cancer

The analysis of the abundance of selected lncRNAs in different clinical stages of CRC is summarized in Fig. 5. After excluding one sample pool due to mixed clinical stages, we observed a general trend of increasing lncRNA levels with disease progression, although they did not reach statistical significance. Notably,* AC055788* demonstrated a trend towards higher abundance in more advanced stages (*P* = 0.052), suggesting potential relevance to disease progression.

**Fig. 6:**
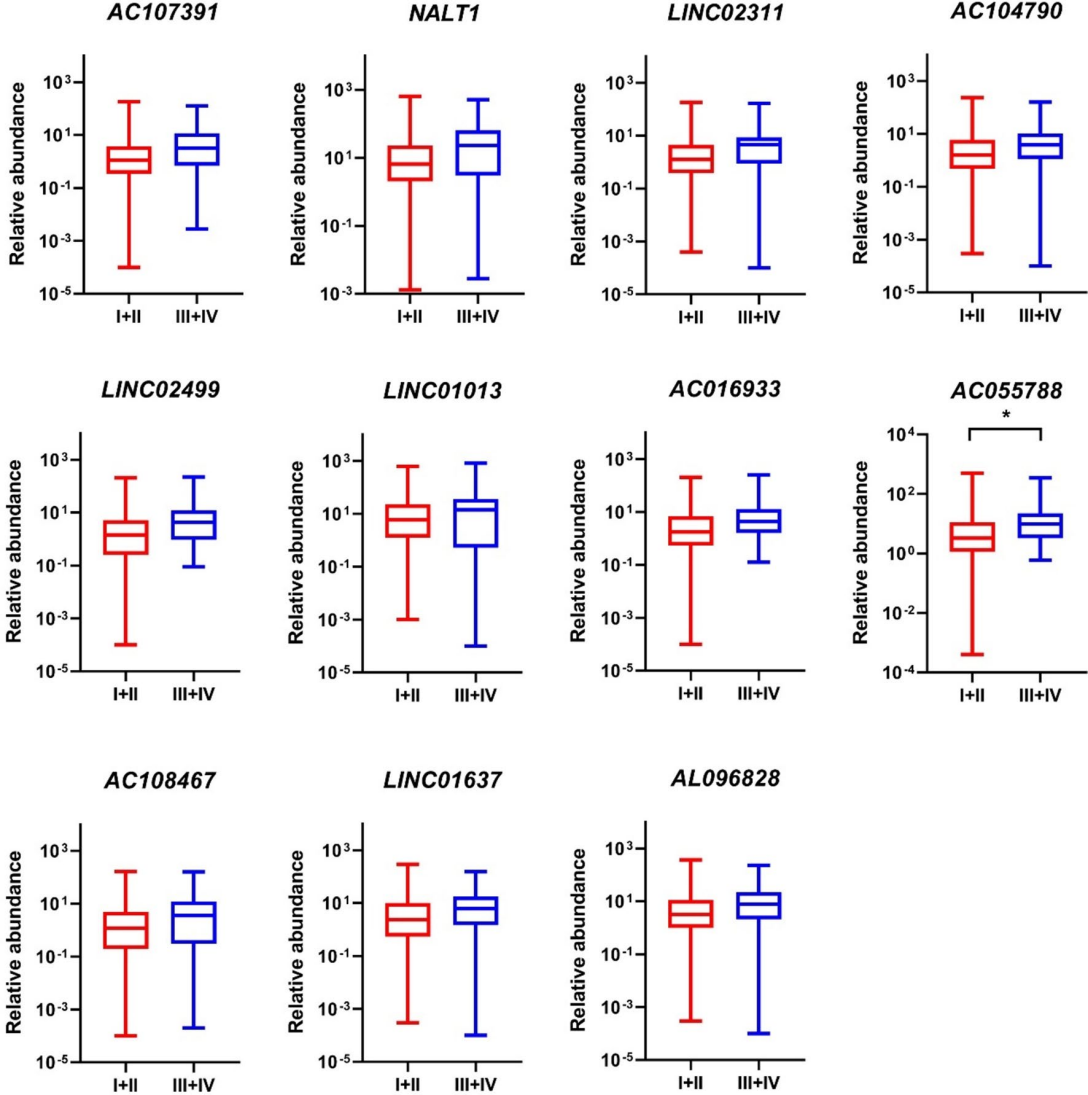
Levels of tested lncRNAs in sEVs of patients with early and advanced stages of CRC. The levels of *AC055788* were significantly increased in advanced stages (III + IV) compared to early stages (I + II) of CRC (*P* = 0.029). *P < 0.05

This increase in lncRNA levels between stage I and IV led to another comparison analysis that evaluated lncRNA content of sEVs at early stages (stage I + II) and advanced stages (stage III + IV) of CRC with the significance level set to *P* < 0.05. The results of the analysis are depicted in Fig. 6. As expected, the significance of lncRNA *AC055788* increased in advanced stages compared to early stages (*P* = 0.029). For the other lncRNAs analyzed, a trend of increased abundance could be observed in advanced stages compared to early stages as well, with *LINC02499* levels approaching borderline significance (*P* = 0.063).

## Discussion

LncRNAs are increasingly considered critical regulators of many cellular functions. In intestinal tissue, they modulate several signaling pathways that are crucial for maintaining its homeostasis [21]. Conversely, their dysregulation in cancer can alter these signaling cascades and allow malignant cells to proliferate and spread [12, 22]. Expression profiling of lncRNAs can identify potential targets that can be used for early disease detection. Despite the increased interest in lncRNA identification, their use as disease biomarkers remains largely unexplored. Importantly, RNA sequencing of EV content offers the possibility of developing biomarkers derived non-invasively from blood serum or plasma.

Our comprehensive study presents significant insights into the roles of lncRNAs from sEVs in CRC. This research was conducted in two phases: an exploratory phase that focused on RNA sequencing and a subsequent validation phase involving a larger cohort for further analysis of identified lncRNAs. However, the process of purifying and characterizing sEVs was an important aspect of our research. For sEV characterization, we used DLS and TEM to determine the size and concentration of EV fractions obtained by SEC from blood serum. Our analyses confirmed the presence of sEVs in samples from both CRC patients and healthy controls. A slightly elevated concentration of sEVs was observed in the samples from CRC patients, which could support the hypothesis that sEVs play a significant role in the pathogenesis of tumor development. Additionally, we identified a marginal subset of particles exhibiting larger sizes, suggesting slight heterogeneity in the vesicle population. Further confirmation of sEVs in our samples was performed by Western blot, which successfully detected key protein markers characteristic of these vesicles. Interestingly, in samples from CRC patients, there was an enhanced signal for CD81, indicative of a potentially higher abundance of sEVs in CRC patients compared to healthy controls. This observation aligns with findings from previous research [23–26]. In the study by Ricklefs et al., the authors demonstrated that in cancer tissues and cell lines, CD81 was significantly upregulated and associated with decreased overall survival. This pattern of increased protein marker expression extends beyond CD81, as shown by Tian et al., who reported higher abundance levels of CD63 in EVs from metastatic breast cancer patients compared to non-metastatic breast cancer patients and healthy donors. Additionally, Odaka and colleagues found that serum levels of CD63-positive EVs were significantly higher in pancreatic ductal adenocarcinoma patients compared to healthy controls.

The detection of a weakly positive calnexin signal in our SEC-isolated samples suggested the possible co-isolation of a different EV subtype, with a different size or distinct biogenesis pathway. To obtain a pure fraction of sEVs, a combination of different approaches is recommended; however, it is not feasible without high losses of vesicles.

Our exploratory cohort aimed to identify the lncRNA profiles in sEVs from blood serum of 76 CRC patients and 29 healthy controls. First, to perform the downstream analysis, we isolated RNA from the obtained sEV samples. However, RNA concentrations were below the detection limit of conventional techniques, so we employed vacuum evaporation to concentrate the samples. Despite lower RNA input, we were able to enhance RNA concentration and successfully prepare sequencing libraries. Using a high-throughput RNA sequencing approach, we detected differences in the sEV levels of 460 genes, which included mainly protein coding genes, lncRNAs, and pseudogenes. This differential analysis was statistically significant (*P* < 0.01), with a majority of these genes showing higher abundance in sEVs from CRC patients. Notably, the analysis revealed that about 20% of genes were lncRNAs, specifically differentiating between patients and healthy controls. Further statistical analysis highlighted the most significantly dysregulated lncRNAs, revealing tumor-specific lncRNAs not yet described in the context of CRC. We have also performed additional bioinformatic analyses related to stage and grade of CRC patients, the results are included in the supplementary data section (Tables S4–S12).

Next, RT-qPCR was used for validation of lncRNAs in larger study cohort of 159 CRC patients and 138 healthy controls. Of the top 20 lncRNAs from exploratory phase, 11 were selected for the validation phase of the study. However, quantifying these lncRNAs through RT-qPCR proved challenging due to their low concentration in the sEV samples. To overcome this, we prepared sample pools based on similar clinicopathological data and concentrated the RNA before cDNA preamplification and qPCR validation. These steps including preamplification enabled the measurement of previously undetectable molecules. However, it is important to note that while preamplification increases the detectability of low-abundance transcripts, it may also introduce artifacts in the amplification process. The RT-qPCR analysis confirmed the upregulation of three lncRNAs (*NALT1*, *AL096828*, and *LINC01637*) in CRC patients, which was in agreement with our sequencing data. Additionally, the RT-qPCR results also revealed elevated levels of *AC055788* and *AC016933*, which were not identified as upregulated in the RNA sequencing analysis. Certain methodological factors, particularly the limited volume of blood serum used for RNA isolation from sEVs and the absence of RNA concentration measurements, might have contributed to the discrepancy observed between our sequencing results and RT-qPCR validation.

The dysregulation of *NALT* expression was explored in the study by Wang et al*.* [27] that demonstrated a significant upregulation of *NALT* in association with *NOTCH1* in human samples in pediatric T cell acute lymphoblastic leukemia. High expression of *NALT* correlated with increased levels of *NOTCH1*, and their interaction promoted cell proliferation both in vitro and in vivo. A similar observation was described by Ye and colleagues [28], who showed upregulated levels of *NALT1* in patients with advanced CRC stage and in CRC cell lines. In their study, *NALT1* contributed to cancer progression by acting as a molecular sponge for *microRNA-574-5p*. This interaction led to increased expression of the *PEG10* gene, promoting CRC cell proliferation, migration, and invasion. In another study [29], *NALT1* was significantly overexpressed in gastric cancer tissues and cells, and this overexpression was closely associated with tumor invasion, metastasis, and poor prognosis in gastric cancer patients. In our study, high-throughput RNA sequencing results supported the findings of the referenced studies, showing higher levels of *NALT1* in cancer patients, specifically in sEVs isolated from peripheral blood of individuals with CRC. Additionally, our validation testing confirmed significantly increased levels of *NALT1* in CRC patients compared to healthy controls. Although not statistically significant, we also observed a higher abundance of *NALT1* in more advanced stages of the disease.

Similar to the reported roles of *NALT1* in various cancers, apart from our CRC findings, dysregulation of *LINC02499* was detected in a hepatocellular cancer (HCC). The study by Ma et al*.* [30] revealed that *LINC02499* was significantly downregulated in HCC and its lower expression was associated with poorer patient survival. Furthermore, the overexpression of *LINC02499 *in vitro had an inhibitory effect on the proliferation, migration, and invasion of HCC cell lines. A similar observation was reported by Zhang et al*.* [31] who showed *LINC02499* to be downregulated in HCC tissues compared to adjacent normal tissues. The authors identified *LINC02499* as the lncRNA most significantly correlated with a range of clinicopathological factors in HCC and demonstrated its significance in predicting overall survival in HCC patients. *LINC02499* was recognized as a protective factor against the progression of the disease. While the function of *LINC02499* has been described in relation to HCC, its role in CRC, particularly in sEVs, remains unexplored. In CRC, we observed a similar downregulation of *LINC02499* in the sequencing analysis of patient-derived sEVs, reflecting its expression pattern in HCC. This could suggest a potentially universal role of *LINC02499* as a tumor suppressor across different cancer types. Despite the lack of confirmation in the validation phase for differences between CRC patients and healthy controls, we observed a noticeable trend indicating *LINC02499*'s differential abundance between early (I + II) and late (III + IV) stages of CRC. This trend was close to reaching statistical significance.

Chung and colleagues [32] found that the lncRNA *LINC01013* was prominently overexpressed in tumor tissue specimens of anaplastic large-cell lymphoma (ALCL), as well as being significantly upregulated in invasive ALCL cell lines. This lncRNA influenced tumor behavior and promoted cell proliferation, suggesting its use as a prognostic marker in ALCL. Similarly, Wang et al*.* [33] showed that *LINC01013* was significantly overexpressed in HCC tumors, and its upregulation was associated with a worse prognosis of HCC patients. Moreover, loss- and gain-of-function experiments revealed that *LINC01013* could promote HCC cell proliferation and tumor progression by enhancing stemness of cells both in vitro and in vivo. In contrast, our sequencing data interestingly revealed that *LINC01013* was significantly downregulated in sEVs isolated from CRC patients compared to healthy controls, suggesting a distinct role of *LINC01013* in CRC. However, this observation was not significant in our validation cohort, highlighting a potential complexity in the behavior of *LINC01013* across different biological matrices and cancer types.

In pancreatic adenocarcinoma (PAAD), *LINC01637*, also known as *XXbac-B135H6.15*, was identified as significant in the study by Deng et al*.* [34]. In this study, the high expression of *LINC01637* was associated with better overall survival in PAAD patients, indicating its potential as a protective factor against disease progression. Additionally, its expression inversely correlated with the increasing risk score in PAAD, suggesting its importance as a potential prognostic biomarker for this type of cancer. Huang et al*.* [35] identified *LINC01637* as being overexpressed in bladder cancer cell lines T24 and J82 compared to a less aggressive cell line of bladder cancer. However, overexpression of *LINC01637* in the cell lines did not translate to enhanced levels in the exosomes derived from these cells. In contrast to its roles in PAAD and bladder cancer, our study investigates *LINC01637* in the context of CRC, specifically examining its abundance in sEVs. The analysis of RNA sequencing data revealed a significant elevation of *LINC01637* in patient samples relative to healthy controls, indicating its distinct role in CRC compared to documented functions in other cancers. Importantly, we validated these findings by a larger study cohort, which confirmed the high abundance of *LINC01637* in sEVs from CRC patient blood serum, suggesting its potential as a non-invasive biomarker in CRC diagnostics.

While our study provides substantial insights into the relative abundance of lncRNAs in sEVs from CRC patients, we have encountered some limitations. Firstly, the pooling of samples, while necessary due to low RNA concentrations, could mask individual variability and relevant differences between patients. This approach, combined with the challenges of quantifying low amounts of RNA, may limit the direct clinical applicability of our findings.

Secondly, while preamplification enables the detection and quantification of RNA molecules that would otherwise be below the threshold of detection, it is not without its drawbacks. This process can introduce amplification biases and non-specific artifacts that can lead to disproportionate representation of certain RNA sequences, which may not accurately reflect their true abundance in the original sample. Despite these challenges, the use of preamplification was a necessary compromise given the current technological constraints and the low RNA yield from sEVs.

Thirdly, although our RNA sequencing approach identified a significant number of lncRNAs with different levels between CRC patients and healthy controls, the analytical power of specific lncRNA for clinical use might be limited. This could be partially due to the technical challenges associated with the isolation of sEV by SEC, which can introduce variability by co-isolation of other EV subtypes. Nevertheless, our findings highlight the biological significance of lncRNAs isolated from sEVs, revealing their potential as non-invasive biomarkers of CRC.

## Conclusions

Our observations across different lncRNAs and cancer types highlight the varied roles of lncRNAs in oncology, offering new perspectives for biomarker discovery and potential therapeutic targets. Importantly, our study demonstrates the potential of EV-enriched lncRNAs as non-invasive biomarkers for distinguishing CRC patients from healthy controls, a finding confirmed in a larger patient cohort. We also recognize that certain methodological aspects, such as the limited volume of blood serum used for sEV RNA isolation and challenges in RNA quantification, have highlighted areas for further optimization.

## Supplementary Information

Below is the link to the electronic supplementary material.

Supplementary file1 (DOCX 466 kb)

## Data Availability

Raw sequencing data were generated at CEITEC Genomics Core Facility and are publicly available at the Sequence Read Archive under accession number PRJNA1071008.

## Abbreviations

CRC: Colorectal cancer
lncRNAs: Long non-coding RNAs
sEVs: Small extracellular vesicles
MMCI: Masaryk Memorial Cancer Institute
SEC: Size exclusion chromatography
TEM: Transmission electron microscopy
DLS: Dynamic light scattering
PdI: Polydispersity index
HCC: Hepatocellular cancer
ALCL: Anaplastic large-cell lymphoma
PAAD: Pancreatic adenocarcinoma
ICD: International Classification of Diseases
