# Supplementary material for: Investigation of long non-coding RNAs in extracellular vesicles from low-volume blood serum specimens of colorectal cancer patients
Source: Clin Exp Med. 2024 Apr 3;24(1):67. doi: 10.1007/s10238-024-01323-1 (PMC10991038; doi:10.1007/s10238-024-01323-1)
Supplement: Supplementary file 1 — Supplementary file1 (DOCX 466 kb) [file 10238_2024_1323_MOESM1_ESM.docx]

# **Investigation of long non-coding RNAs in extracellular vesicles from low-volume blood serum specimens of colorectal cancer patients**

Marie Boudna^1,2^, Tana Machackova^1^, Petra Vychytilova-Faltejskova^1,2^, Karolina Trachtova^1^, Renata Bartosova^1^, Tina Catela Ivkovic^1^, Dagmar Al Tukmachi^1^, Robin Jugas^1^, Lucie Pifkova^1^, Jana Orlickova^1^, Jan Kotoucek^3^, Marketa Pavlikova^1^, Milana Sachlova^4^, Lucia Bohovicova^5^, Teodor Stanek^6^, Jana Halamkova^5^, Igor Kiss^5^, Vladimir Prochazka^7^, Martin Svoboda^7^, Zdenek Kala^7^, Kamila Souckova^1^* and Ondrej Slaby^1,2^*

**Supplementary Material**

**Supplementary Figures**

**
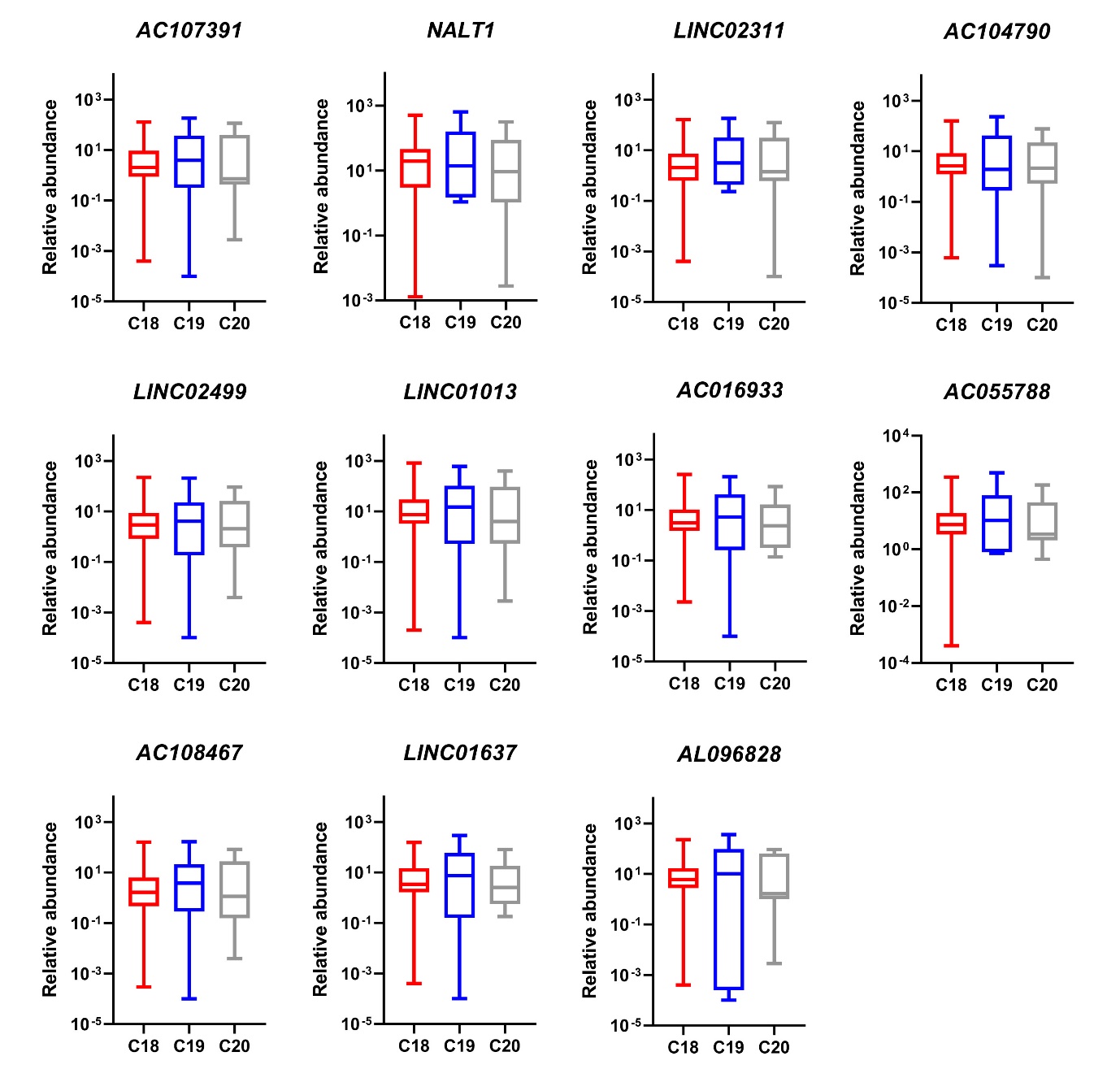
**

**Fig. S1** Box plots showing the levels of lncRNAs in EVs of CRC patients according to tumour localization (P < 0.05). C18 - malignant neoplasm of the colon; C19 - malignant neoplasm of the rectosigmoid; C20 - malignant neoplasm of the rectum.

**Supplementary Tables**

**Table S1** Selected lncRNAs with primer sequences used for RT-qPCR analysis in the validation phase.

| **Gene name** | **Primer sequence** |
| --- | --- |
| NALT1 | FWD-TCCAGATGTGGTTTCAGTGTC |
|  | REV-CCGAATCCATCCTAGAGCAAG |
| LINC02311 | FWD-CTCCCTAACATGATACATATACAAAGAC |
|  | REV-ACCATATTCTTAGATGAGTGGTTCA |
| AC104790.1 | FWD-TTGGTTTCTTCCTTATGAATCATGG |
|  | REV-GTCAGCTCAAGGTCCAGAAA |
| LINC01637 | FWD-GGAACCAGAATAATCAGCCCTAA |
|  | REV-TCTCACCTGTCATTTACCAGAAC |
| LINC02499 | FWD-AAGCAACTCATGGAGAAGTACAA |
|  | REV-GGTGAGTGCATTCTGAAGGTAA |
| LINC01013 | FWD-GTAAGTGTTGTTGCTTAGTGTGTC |
|  | REV-TTTCTGATGGTCAGTGGCTAAA |
| AC016933.1 | FWD-CCAGGAAGTGAAGCGAGATTAT |
|  | REV-TGTGCCAGATAGGACCTCA |
| AC055788.1 | FWD-GAGTTCCGCTCTGGGAAGT |
|  | REV-CCCGAGAGATCAGAAGCCT |
| AL096828.1 | FWD-ATGCAGGGCAGTCCATGTT |
|  | REV-CATGTGCCACAGGGTCCAC |
| AC108467.1 | FWD-ATGAAGTGAGGGTACATGGAAA |
|  | REV-CTCAGAAGTCTAGAGGGTAGAAATG |
| AC107391.1 | FWD-CGCATCCTCAGAAGCTGTAA |
|  | REV-CTCTCCCAAGGTGTCAAACTC |

**Table S2** Sample pools with clinicopathological data of CRC patients and healthy controls enrolled in the validation phase.

| CRC Patient pools | Gender | Age | Diagnosis | Stage | Grade |
| --- | --- | --- | --- | --- | --- |
| 1 |  |  |  |  |  |
|  | female | 75 | C18 | I | 1 |
|  | female | 68 | C18 | I | 1 |
|  | female | 82 | C18 | I | 2 |
| 2 |  |  |  |  |  |
|  | male | 76 | C18 | I | 2 |
|  | male | 73 | C18 | I | 2 |
|  | male | 74 | C18 | I | 2 |
| 3 |  |  |  |  |  |
|  | male | 65 | C18 | I | 2 |
|  | male | 66 | C18 | I | 2 |
|  | male | 69 | C18 | I | 1 |
| 4 |  |  |  |  |  |
|  | male | 57 | C18 | III | 2 |
|  | male | 80 | C19 | III | 2 |
|  | male | 32 | C18 | II | 2 |
| 5 |  |  |  |  |  |
|  | male | 55 | C18 | I | 1 |
|  | male | 62 | C18 | I | 1 |
|  | female | 37 | C19 | I | 2 |
| 6 |  |  |  |  |  |
|  | female | 76 | C20 | I | 1 |
|  | female | 62 | C20 | I | 2 |
|  | female | 74 | C20 | I | 2 |
| 7 |  |  |  |  |  |
|  | male | 49 | C20 | I | 1 |
|  | male | 54 | C20 | I | 1 |
|  | male | 56 | C20 | I | 1 |
| 8 |  |  |  |  |  |
|  | male | 63 | C20 | I | 1 |
|  | male | 69 | C20 | I | 2 |
|  | male | 66 | C20 | I | 2 |
| 9 |  |  |  |  |  |
|  | male | 77 | C20 | I | 2 |
|  | male | 83 | C20 | I | 1 |
|  | male | 76 | C20 | I | 2 |
| 10 |  |  |  |  |  |
|  | male | 60 | C20 | I | 2 |
|  | male | 73 | C20 | I | 2 |
|  | male | 60 | C20 | I | 2 |
| 11 |  |  |  |  |  |
|  | female | 56 | C18 | II | 3 |
|  | female | 79 | C18 | II | 3 |
|  | female | 80 | C18 | II | 2 |
| 12 |  |  |  |  |  |
|  | male | 65 | C18 | II | 3 |
|  | male | 64 | C18 | II | 1 |
|  | male | 65 | C18 | II | 2 |
| 13 |  |  |  |  |  |
|  | male | 73 | C18 | II | 1 |
|  | male | 71 | C18 | II | 1 |
|  | male | 72 | C18 | II | 2 |
| 14 |  |  |  |  |  |
|  | male | 68 | C18 | II | 2 |
|  | male | 70 | C18 | II | 1 |
|  | male | 63 | C18 | II | 2 |
| 15 |  |  |  |  |  |
|  | male | 54 | C18 | II | 1 |
|  | male | 44 | C18 | II | 2 |
|  | male | 51 | C18 | II | 2 |
| 16 |  |  |  |  |  |
|  | male | 64 | C19 | II | 2 |
|  | male | 68 | C19 | II | 2 |
|  | male | 60 | C19 | II | 2 |
| 17 |  |  |  |  |  |
|  | male | 71 | C19 | II | x |
|  | male | 71 | C19 | II | 1 |
|  | male | 75 | C19 | II | 2 |
| 18 |  |  |  |  |  |
|  | female | 78 | C19 | II | 1 |
|  | female | 71 | C19 | II | 2 |
|  | female | 71 | C19 | II | 1 |
| 19 |  |  |  |  |  |
|  | female | 70 | C19 | II | 3 |
|  | female | 64 | C19 | II | 1 |
|  | male | 47 | C19 | II | 2 |
| 20 |  |  |  |  |  |
|  | female | 59 | C20 | II | 3 |
|  | female | 66 | C20 | II | 3 |
|  | female | 64 | C20 | II | 2 |
| 21 |  |  |  |  |  |
|  | female | 73 | C20 | II | 2 |
|  | female | 75 | C20 | II | 1 |
|  | female | 69 | C20 | II | 2 |
| 22 |  |  |  |  |  |
|  | female | 53 | C18 | III | 2 |
|  | female | 51 | C18 | III | 2 |
|  | female | 59 | C18 | III | 1 |
| 23 |  |  |  |  |  |
|  | female | 70 | C18 | III | 2 |
|  | female | 82 | C18 | III | x |
|  | female | 67 | C18 | III | 2 |
| 24 |  |  |  |  |  |
|  | female | 70 | C18 | III | 2 |
|  | female | 64 | C18 | III | 1 |
|  | female | 67 | C18 | III | 2 |
| 25 |  |  |  |  |  |
|  | male | 63 | C18 | III | 1 |
|  | male | 64 | C18 | III | 2 |
|  | male | 61 | C18 | III | 1 |
| 26 |  |  |  |  |  |
|  | male | 67 | C18 | III | 1 |
|  | male | 68 | C18 | III | 2 |
|  | male | 68 | C18 | III | 2 |
| 27 |  |  |  |  |  |
|  | male | 79 | C18 | III | 2 |
|  | male | 82 | C18 | III | 2 |
|  | male | 84 | C18 | III | 2 |
| 28 |  |  |  |  |  |
|  | male | 60 | C18 | III | 2 |
|  | male | 60 | C18 | III | x |
|  | male | 60 | C18 | III | 2 |
| 29 |  |  |  |  |  |
|  | male | 72 | C18 | III | 2 |
|  | male | 71 | C18 | III | 3 |
|  | male | 73 | C18 | III | 3 |
| 30 |  |  |  |  |  |
|  | male | 74 | C18 | III | 2 |
|  | male | 76 | C18 | III | 3 |
|  | male | 67 | C18 | III | 2 |
| 31 |  |  |  |  |  |
|  | male | 59 | C18 | III | 1 |
|  | male | 58 | C18 | III | 2 |
|  | male | 59 | C18 | III | 2 |
| 32 |  |  |  |  |  |
|  | male | 70 | C19 | III | 1 |
|  | male | 66 | C19 | III | 2 |
|  | male | 70 | C19 | III | 2 |
| 33 |  |  |  |  |  |
|  | male | 55 | C19 | III | 2 |
|  | male | 53 | C19 | III | 2 |
|  | male | 78 | C19 | III | x |
| 34 |  |  |  |  |  |
|  | female | 54 | C19 | III | 3 |
|  | female | 52 | C19 | III | 2 |
|  | female | 45 | C19 | III | 1 |
| 35 |  |  |  |  |  |
|  | male | 77 | C20 | III | 2 |
|  | male | 78 | C20 | III | 2 |
|  | male | 79 | C20 | III | 2 |
| 36 |  |  |  |  |  |
|  | male | 62 | C18 | IV | 3 |
|  | male | 62 | C18 | IV | 2 |
|  | male | 64 | C18 | IV | 3 |
| 37 |  |  |  |  |  |
|  | male | 78 | C18 | IV | 3 |
|  | male | 77 | C18 | IV | 2 |
|  | male | 78 | C18 | IV | 2 |
| 38 |  |  |  |  |  |
|  | male | 58 | C18 | IV | 3 |
|  | male | 48 | C18 | IV | 2 |
|  | male | 49 | C18 | IV | 2 |
| 39 |  |  |  |  |  |
|  | female | 55 | C18 | IV | x |
|  | female | 71 | C18 | IV | 3 |
|  | female | 68 | C18 | IV | 2 |
| 40 |  |  |  |  |  |
|  | female | 74 | C18 | IV | 2 |
|  | female | 61 | C18 | IV | 3 |
|  | female | 72 | C18 | IV | 3 |
| 41 |  |  |  |  |  |
|  | female | 50 | C18 | IV | 4 |
|  | female | 58 | C18 | IV | 3 |
|  | female | 51 | C18 | IV | x |
| 42 |  |  |  |  |  |
|  | male | 47 | C19 | IV | 2 |
|  | male | 46 | C19 | IV | 2 |
|  | male | 58 | C19 | IV | 1 |
| 43 |  |  |  |  |  |
|  | male | 72 | C19 | IV | 2 |
|  | male | 72 | C19 | IV | 3 |
|  | male | 76 | C19 | IV | 1 |
| 44 |  |  |  |  |  |
|  | female | 59 | C19 | IV | 2 |
|  | female | 67 | C19 | IV | 3 |
|  | male | 76 | C18 | IV | 1 |
| 45 |  |  |  |  |  |
|  | female | 46 | C20 | IV | 2 |
|  | female | 66 | C20 | IV | 2 |
|  | female | 61 | C20 | IV | 3 |
| 46 |  |  |  |  |  |
|  | male | 83 | C20 | IV | 1 |
|  | male | 79 | C20 | IV | 2 |
|  | male | 79 | C20 | IV | 3 |
| 47 |  |  |  |  |  |
|  | female | 68 | C18 | II | 3 |
|  | female | 43 | C18 | II | 1 |
|  | female | 71 | C18 | II | 2 |
| 48 |  |  |  |  |  |
|  | male | 71 | C20 | II | 2 |
|  | male | 66 | C20 | II | 1 |
|  | male | 64 | C20 | II | 2 |
| 49 |  |  |  |  |  |
|  | male | 65 | C20 | II | 1 |
|  | male | 71 | C20 | II | 2 |
|  | male | 66 | C20 | II | 2 |
| 50 |  |  |  |  |  |
|  | male | 60 | C20 | II | 2 |
|  | male | 53 | C20 | II | 2 |
|  | male | 76 | C20 | II | 2 |
| 51 |  |  |  |  |  |
|  | male | 73 | C18 | II | 1 |
|  | male | 58 | C18 | II | 2 |
|  | male | 68 | C18 | II | 2 |
| 52 |  |  |  |  |  |
|  | male | 49 | C20 | III | 3 |
|  | female | 43 | C20 | III | 3 |
|  | female | 68 | C20 | III | 3 |
| 53 |  |  |  |  |  |
|  | male | 61 | C20 | I | 1 |
|  | female | 75 | C20 | I | 1 |
|  | female | 38 | C20 | I | 2 |
|  |  |  |  |  |  |

| Healthy control pools | Gender | Age |
| --- | --- | --- |
| 1 |  |  |
|  | female | 54 |
|  | female | 54 |
|  | female | 54 |
| 2 |  |  |
|  | female | 51 |
|  | female | 51 |
|  | female | 51 |
| 3 |  |  |
|  | female | 52 |
|  | female | 52 |
|  | female | 52 |
| 4 |  |  |
|  | female | 50 |
|  | female | 50 |
|  | female | 50 |
| 5 |  |  |
|  | female | 50 |
|  | female | 50 |
|  | female | 50 |
| 6 |  |  |
|  | female | 52 |
|  | female | 53 |
|  | female | 52 |
| 7 |  |  |
|  | female | 58 |
|  | female | 58 |
|  | female | 58 |
| 8 |  |  |
|  | female | 58 |
|  | female | 59 |
|  | female | 59 |
| 9 |  |  |
|  | female | 60 |
|  | female | 60 |
|  | female | 60 |
| 10 |  |  |
|  | female | 57 |
|  | female | 57 |
|  | female | 57 |
| 11 |  |  |
|  | female | 57 |
|  | female | 57 |
|  | female | 57 |
| 12 |  |  |
|  | female | 56 |
|  | female | 56 |
|  | female | 56 |
| 13 |  |  |
|  | female | 61 |
|  | female | 61 |
|  | female | 61 |
| 14 |  |  |
|  | female | 61 |
|  | female | 61 |
|  | female | 61 |
| 15 |  |  |
|  | female | 72 |
|  | female | 73 |
|  | female | 73 |
| 16 |  |  |
|  | female | 67 |
|  | female | 68 |
|  | female | 67 |
| 17 |  |  |
|  | female | 77 |
|  | female | 74 |
|  | female | 77 |
| 18 |  |  |
|  | female | 62 |
|  | female | 62 |
|  | female | 62 |
| 19 |  |  |
|  | female | 63 |
|  | female | 64 |
|  | female | 64 |
| 20 |  |  |
|  | female | 69 |
|  | female | 68 |
|  | female | 70 |
| 21 |  |  |
|  | female | 62 |
|  | female | 61 |
|  | female | 60 |
| 22 |  |  |
|  | female | 65 |
|  | female | 66 |
|  | female | 65 |
| 23 |  |  |
|  | male | 53 |
|  | male | 53 |
|  | male | 54 |
| 24 |  |  |
|  | male | 52 |
|  | male | 52 |
|  | male | 53 |
| 25 |  |  |
|  | male | 50 |
|  | male | 50 |
|  | male | 50 |
| 26 |  |  |
|  | male | 52 |
|  | male | 52 |
|  | male | 51 |
| 27 |  |  |
|  | male | 51 |
|  | male | 51 |
|  | male | 50 |
| 28 |  |  |
|  | male | 56 |
|  | male | 55 |
|  | male | 55 |
| 29 |  |  |
|  | male | 65 |
|  | male | 65 |
|  | male | 65 |
| 30 |  |  |
|  | male | 57 |
|  | male | 56 |
|  | male | 56 |
| 31 |  |  |
|  | male | 66 |
|  | male | 66 |
|  | male | 66 |
| 32 |  |  |
|  | male | 71 |
|  | male | 71 |
|  | male | 71 |
| 33 |  |  |
|  | male | 71 |
|  | male | 68 |
|  | male | 68 |
| 34 |  |  |
|  | male | 67 |
|  | male | 67 |
|  | male | 66 |
| 35 |  |  |
|  | male | 57 |
|  | male | 57 |
|  | male | 57 |
| 36 |  |  |
|  | male | 78 |
|  | male | 81 |
|  | male | 73 |
| 37 |  |  |
|  | male | 64 |
|  | male | 64 |
|  | male | 64 |
| 38 |  |  |
|  | male | 65 |
|  | male | 65 |
|  | male | 64 |
| 39 |  |  |
|  | male | 60 |
|  | male | 60 |
|  | male | 60 |
| 40 |  |  |
|  | male | 60 |
|  | male | 60 |
|  | male | 60 |
| 41 |  |  |
|  | male | 58 |
|  | male | 58 |
|  | male | 58 |
| 42 |  |  |
|  | male | 57 |
|  | male | 57 |
|  | male | 57 |
| 43 |  |  |
|  | male | 59 |
|  | male | 60 |
|  | male | 60 |
| 44 |  |  |
|  | male | 58 |
|  | male | 58 |
|  | male | 58 |
| 45 |  |  |
|  | male | 56 |
|  | male | 56 |
|  | male | 57 |
| 46 |  |  |
|  | male | 58 |
|  | male | 57 |
|  | male | 57 |
|  |  |  |

**Table S3** The list of differentially expressed lncRNAs from RNA-sequencing.

|  | baseMean | log2FoldChange | pvalue | padj | gene_name | gene_type |
| --- | --- | --- | --- | --- | --- | --- |
| ENSG00000251310.1 | 70.79714 | -0.47005 | 3.24E-05 |  | AC107391.1 | lncRNA |
| ENSG00000251471.1 | 138.898 | -0.31983 | 5.75E-05 |  | AC016933.1 | lncRNA |
| ENSG00000250436.1 | 143.3127 | -0.32737 | 8.42E-05 |  | LINC02499 | lncRNA |
| ENSG00000285596.1 | 50.71911 | 0.392122 | 8.69E-05 |  | AC017116.2 | lncRNA |
| ENSG00000286937.1 | 275.5368 | -0.32172 | 0.000103 |  | AC055788.1 | lncRNA |
| ENSG00000259582.4 | 89.6075 | -0.42648 | 0.000147 |  | AC026523.1 | lncRNA |
| ENSG00000224409.1 | 77.23061 | 0.311164 | 0.000178 |  | AC114489.1 | lncRNA |
| ENSG00000258718.3 | 72.40041 | -0.41274 | 0.000252 |  | LINC02311 | lncRNA |
| ENSG00000237476.1 | 100.3727 | 0.395134 | 0.000268 |  | LINC01637 | lncRNA |
| ENSG00000224970.2 | 55.54051 | 0.375554 | 0.000271 |  | AC245427.1 | lncRNA |
| ENSG00000286901.1 | 53.8021 | 0.443705 | 0.000312 |  | Z69667.1 | lncRNA |
| ENSG00000259230.1 | 125.9439 | 0.302789 | 0.000323 |  | LINC02323 | lncRNA |
| ENSG00000237390.1 | 80.75883 | 0.331853 | 0.000408 |  | AL139130.1 | lncRNA |
| ENSG00000228495.2 | 195.5934 | -0.3316 | 0.000461 |  | LINC01013 | lncRNA |
| ENSG00000237886.1 | 58.67829 | 0.586881 | 0.000479 |  | NALT1 | lncRNA |
| ENSG00000231113.2 | 79.66514 | 0.302647 | 0.000578 |  | AL035587.1 | lncRNA |
| ENSG00000281778.1 | 87.42012 | -0.3819 | 0.000616 |  | LINC00550 | lncRNA |
| ENSG00000196756.13 | 158.974 | 0.316803 | 0.000651 |  | SNHG17 | lncRNA |
| ENSG00000285998.1 | 62.84302 | -0.45433 | 0.000692 |  | AC104790.1 | lncRNA |
| ENSG00000253879.2 | 78.06521 | -0.40954 | 0.000696 |  | AC087664.2 | lncRNA |
| ENSG00000236453.5 | 246.7591 | -0.31331 | 0.000766 |  | AC003092.1 | lncRNA |
| ENSG00000234698.1 | 58.88598 | 0.331116 | 0.00083 |  | AL161937.1 | lncRNA |
| ENSG00000248744.1 | 191.7919 | -0.32787 | 0.000848 |  | AC108467.1 | lncRNA |
| ENSG00000255606.1 | 84.59203 | 0.318016 | 0.000911 |  | AP000439.1 | lncRNA |
| ENSG00000230107.1 | 109.7164 | 0.38223 | 0.001047 |  | AL022316.1 | lncRNA |
| ENSG00000267108.1 | 57.61405 | 0.356797 | 0.00106 |  | AP001029.1 | lncRNA |
| ENSG00000231742.6 | 54.17916 | 0.336034 | 0.001147 |  | LINC01273 | lncRNA |
| ENSG00000227459.1 | 52.80797 | 0.35786 | 0.001151 |  | AC079612.2 | lncRNA |
| ENSG00000279141.3 | 71.00212 | 0.483364 | 0.001187 |  | LINC01451 | lncRNA |
| ENSG00000231977.1 | 189.1824 | 0.34883 | 0.001253 |  | AL096828.1 | lncRNA |
| ENSG00000229414.2 | 146.7232 | 0.307769 | 0.001268 |  | KCNQ1-AS1 | lncRNA |
| ENSG00000258498.8 | 93.10765 | 0.37252 | 0.001289 |  | DIO3OS | lncRNA |
| ENSG00000248757.2 | 68.61575 | -0.38211 | 0.001301 |  | LINC02115 | lncRNA |
| ENSG00000267750.6 | 74.60155 | 0.300071 | 0.00137 |  | RUNDC3A-AS1 | lncRNA |
| ENSG00000186235.11 | 89.07374 | 0.3312 | 0.001464 |  | LINC02610 | lncRNA |
| ENSG00000205300.3 | 54.73743 | 0.390527 | 0.001488 |  | AL356414.1 | lncRNA |
| ENSG00000231133.8 | 122.0314 | 0.308974 | 0.001595 |  | HAR1B | lncRNA |
| ENSG00000254366.7 | 625.1662 | -0.32999 | 0.001794 | 0.046499 | AC062004.1 | lncRNA |
| ENSG00000288560.1 | 469.7857 | -0.3045 | 0.00197 |  | AL360015.1 | lncRNA |
| ENSG00000287178.1 | 210.6413 | -0.3276 | 0.002 |  | AC026320.3 | lncRNA |
| ENSG00000260630.7 | 173.1362 | 0.304852 | 0.002208 |  | SNAI3-AS1 | lncRNA |
| ENSG00000287356.1 | 85.40518 | 0.425383 | 0.002258 |  | AL590822.3 | lncRNA |
| ENSG00000275239.4 | 88.58994 | 0.34991 | 0.002361 |  | FAM242F | lncRNA |
| ENSG00000253407.2 | 78.80354 | -0.37658 | 0.002857 |  | AC087341.1 | lncRNA |
| ENSG00000234311.1 | 84.25168 | 0.337863 | 0.003081 |  | AL451069.3 | lncRNA |
| ENSG00000235056.1 | 102.7556 | -0.37895 | 0.003147 |  | AC010983.1 | lncRNA |
| ENSG00000183250.12 | 87.99597 | 0.346636 | 0.003354 |  | LINC01547 | lncRNA |
| ENSG00000250775.2 | 304.7562 | -0.31088 | 0.003444 |  | AC093730.1 | lncRNA |
| ENSG00000242339.2 | 67.22181 | -0.30951 | 0.003588 |  | LINC02025 | lncRNA |
| ENSG00000234584.1 | 55.55787 | -0.33312 | 0.003847 |  | AC019186.1 | lncRNA |
| ENSG00000253894.2 | 53.53423 | -0.31807 | 0.003848 |  | AC011124.2 | lncRNA |
| ENSG00000234520.6 | 187.1592 | -0.30229 | 0.004672 |  | HRAT17 | lncRNA |
| ENSG00000227227.1 | 77.13236 | -0.31283 | 0.004838 |  | AC017101.1 | lncRNA |
| ENSG00000286872.1 | 51.33291 | 0.307328 | 0.004989 |  | AC024270.4 | lncRNA |
| ENSG00000197251.3 | 53.58082 | 0.328377 | 0.004999 |  | LINC00336 | lncRNA |
| ENSG00000250241.6 | 346.9547 | -0.3032 | 0.00512 |  | AC105383.1 | lncRNA |
| ENSG00000227244.2 | 51.99291 | -0.45124 | 0.00521 |  | LINC00845 | lncRNA |
| ENSG00000262223.8 | 113.3801 | 0.316801 | 0.005277 |  | AC110285.1 | lncRNA |
| ENSG00000223764.2 | 56.3454 | 0.353253 | 0.005443 |  | LINC02593 | lncRNA |
| ENSG00000258532.2 | 61.26005 | -0.31938 | 0.005783 |  | LINC02305 | lncRNA |
| ENSG00000285798.1 | 218.9423 | -0.33333 | 0.005974 |  | AC092958.4 | lncRNA |
| ENSG00000259052.1 | 108.304 | 0.325728 | 0.006046 |  | AL157871.6 | lncRNA |
| ENSG00000285462.1 | 107.4841 | -0.31006 | 0.006101 |  | LINC02792 | lncRNA |
| ENSG00000235237.1 | 65.26878 | 0.335496 | 0.006155 |  | Z82188.2 | lncRNA |
| ENSG00000261713.6 | 152.9537 | 0.323436 | 0.00626 |  | SSTR5-AS1 | lncRNA |
| ENSG00000238164.6 | 61.42043 | 0.337521 | 0.006375 |  | TNFRSF14-AS1 | lncRNA |
| ENSG00000280916.2 | 80.31425 | 0.327487 | 0.007059 |  | FOXCUT | lncRNA |
| ENSG00000286621.1 | 51.74785 | 0.316153 | 0.007171 |  | AC064843.1 | lncRNA |
| ENSG00000264634.1 | 76.53777 | -0.33616 | 0.00732 |  | AC100844.1 | lncRNA |
| ENSG00000241636.1 | 71.73161 | -0.34222 | 0.007525 |  | LINC01323 | lncRNA |
| ENSG00000273550.2 | 358.6353 | -0.30199 | 0.007825 |  | AL354810.1 | lncRNA |
| ENSG00000285598.1 | 68.64884 | -0.30607 | 0.008327 |  | AL023283.1 | lncRNA |
| ENSG00000287321.1 | 63.67091 | -0.33061 | 0.008444 |  | AC026523.4 | lncRNA |
| ENSG00000225285.1 | 50.6308 | 0.373673 | 0.008503 |  | LINC01770 | lncRNA |
| ENSG00000250107.1 | 60.88898 | 0.335369 | 0.008648 |  | CACNA1G-AS1 | lncRNA |
| ENSG00000234880.1 | 55.75435 | 0.396075 | 0.008733 |  | LINC00163 | lncRNA |
| ENSG00000287520.1 | 87.01345 | -0.37023 | 0.008999 |  | AC093765.4 | lncRNA |
| ENSG00000205562.3 | 59.6976 | -0.30992 | 0.009004 |  | AL049775.1 | lncRNA |
| ENSG00000248456.1 | 58.26881 | -0.30423 | 0.009392 |  | LINC02485 | lncRNA |
| ENSG00000184856.7 | 84.96069 | -0.3206 | 0.009423 |  | LINC00308 | lncRNA |
| ENSG00000244650.2 | 140.7284 | -0.30998 | 0.009464 |  | AC025566.1 | lncRNA |
| ENSG00000287290.1 | 154.6253 | -0.33359 | 0.0097 |  | AC093765.3 | lncRNA |

| gene_id | baseMean | log2FoldChange | pvalue | padj | gene_name |
| --- | --- | --- | --- | --- | --- |
| ENSG00000258464.2 | 219.4467 | -0.30301 | 0.000649 | 0.999967 | AL160237.1 |
| ENSG00000285999.1 | 51.54052 | 0.550246 | 0.000887 | 0.999967 | AC025442.2 |
| ENSG00000251256.1 | 94.73982 | -0.3877 | 0.001555 | 0.999967 | LINC02358 |
| ENSG00000265489.1 | 155.1319 | -0.30371 | 0.001661 | 0.999967 | AC005358.1 |
| ENSG00000251562.8 | 367.0497 | 1.360375 | 0.002481 | 0.999967 | MALAT1 |
| ENSG00000227888.4 | 78.90924 | -0.87731 | 0.002997 | 0.999967 | FAM66A |
| ENSG00000235097.1 | 96.56196 | -0.33302 | 0.005421 | 0.999967 | LINC00330 |
| ENSG00000287285.1 | 54.83215 | -0.34845 | 0.012517 | 0.999967 | AP000547.4 |
| ENSG00000264265.1 | 53.70626 | 0.428368 | 0.012771 | 0.999967 | LINC01925 |
| ENSG00000287707.1 | 55.88379 | -0.32477 | 0.016208 | 0.999967 | AC115284.4 |
| ENSG00000285930.1 | 88.36718 | -0.30955 | 0.017253 | 0.999967 | AC015813.8 |
| ENSG00000288548.1 | 62.68197 | 0.320792 | 0.019921 | 0.999967 | AL035653.1 |
| ENSG00000232212.1 | 203.0616 | 0.392299 | 0.020678 | 0.999967 | LINC01701 |
| ENSG00000261026.1 | 55.07969 | -0.30714 | 0.020937 | 0.999967 | AC105046.1 |
| ENSG00000285998.1 | 58.77148 | -0.39382 | 0.021236 | 0.999967 | AC104790.1 |
| ENSG00000253123.4 | 52.30942 | -0.36071 | 0.021924 | 0.999967 | AC091182.1 |
| ENSG00000287370.1 | 132.4442 | -0.37075 | 0.022263 | 0.999967 | AL356317.1 |
| ENSG00000255399.4 | 55.31846 | -0.36686 | 0.023547 | 0.999967 | TBX5-AS1 |
| ENSG00000285533.2 | 94.23732 | 0.314021 | 0.024199 | 0.999967 | RELA-DT |
| ENSG00000264920.2 | 50.76216 | 0.322605 | 0.024702 | 0.999967 | AC018521.5 |
| ENSG00000248533.1 | 80.76997 | 0.323112 | 0.024876 | 0.999967 | AC034226.1 |
| ENSG00000257762.6 | 56.9517 | -0.32344 | 0.026265 | 0.999967 | LINC02401 |
| ENSG00000231711.2 | 68.4021 | 0.341264 | 0.027403 | 0.999967 | LINC00899 |
| ENSG00000236354.1 | 67.64681 | -0.30741 | 0.031832 | 0.999967 | LINC00437 |
| ENSG00000267626.1 | 58.72486 | 0.32404 | 0.037235 | 0.999967 | AC002115.1 |
| ENSG00000251665.1 | 58.39671 | 0.388102 | 0.039238 | 0.999967 | AC005920.3 |
| ENSG00000233791.5 | 67.51096 | 0.302188 | 0.039641 | 0.999967 | LINC01136 |
| ENSG00000255284.2 | 72.89565 | 0.323871 | 0.040553 | 0.999967 | AP006621.3 |
| ENSG00000260604.2 | 64.97533 | -0.32695 | 0.044133 | 0.999967 | AL590004.3 |
| ENSG00000261173.1 | 53.77494 | -0.31434 | 0.044714 | 0.999967 | AC018845.3 |
| ENSG00000287246.1 | 60.02091 | 0.340741 | 0.046778 | 0.999967 | AC022535.3 |
| ENSG00000285889.1 | 60.88769 | 0.324001 | 0.047486 | 0.999967 | AL355312.4 |

**Table S4** The list of differentially expressed lncRNAs between stage I and stage II of CRC patients detected in serum sEVs.

**Table S5** The list of differentially expressed lncRNAs between stage I and stage III of CRC patients detected in serum sEVs.

| gene_id | baseMean | log2FoldChange | pvalue | padj | gene_name |
| --- | --- | --- | --- | --- | --- |
| ENSG00000255983.1 | 141.1068 | 0.406431 | 0.000225 | 0.99999785 | AC007848.1 |
| ENSG00000287925.1 | 146.5728 | 0.326028 | 0.000304 | 0.99999785 | AL353576.1 |
| ENSG00000255491.3 | 81.65491 | -0.33653 | 0.005996 | 0.99999785 | AC100858.2 |
| ENSG00000227713.2 | 57.89708 | 0.433036 | 0.010001 | 0.99999785 | AC092159.1 |
| ENSG00000255355.1 | 65.00846 | 0.301224 | 0.01078 | 0.99999785 | AP000640.2 |
| ENSG00000287463.1 | 61.83346 | -0.3553 | 0.011158 | 0.99999785 | AC110620.2 |
| ENSG00000286480.1 | 52.16032 | -0.45862 | 0.014481 | 0.99999785 | AC009501.2 |
| ENSG00000256695.2 | 78.07485 | -0.35392 | 0.015517 | 0.99999785 | AC003982.1 |
| ENSG00000286381.1 | 57.57536 | -0.30935 | 0.017256 | 0.99999785 | AL078622.1 |
| ENSG00000244513.7 | 57.9862 | -0.4003 | 0.019474 | 0.99999785 | AC109587.1 |
| ENSG00000283999.1 | 60.58742 | 0.323455 | 0.024495 | 0.99999785 | AL358215.3 |
| ENSG00000288549.1 | 60.93308 | 0.339304 | 0.026395 | 0.99999785 | AC026412.4 |
| ENSG00000264920.2 | 50.76216 | 0.315354 | 0.033907 | 0.99999785 | AC018521.5 |
| ENSG00000249276.1 | 67.45415 | 0.434258 | 0.035676 | 0.99999785 | AC104119.1 |
| ENSG00000234752.1 | 89.09894 | 0.312164 | 0.040905 | 0.99999785 | LINC02676 |
| ENSG00000237281.1 | 88.00073 | -0.30048 | 0.041281 | 0.99999785 | CATIP-AS2 |
| ENSG00000234494.7 | 103.0478 | -0.30793 | 0.042959 | 0.99999785 | SP2-AS1 |
| ENSG00000255516.1 | 65.42387 | 0.360413 | 0.043784 | 0.99999785 | AP005436.3 |
| ENSG00000285563.1 | 66.63141 | -0.3136 | 0.04528 | 0.99999785 | AC106872.12 |

**Table S6** The list of differentially expressed lncRNAs between stage I and stage IV of CRC patients detected in serum sEVs.

| gene_id | baseMean | log2FoldChange | pvalue | padj | gene_name |
| --- | --- | --- | --- | --- | --- |
| ENSG00000283757.1 | 107.2098 | -0.47176 | 0.000129 | 0.828748 | AL031686.1 |
| ENSG00000267827.5 | 98.33099 | -0.44035 | 0.000835 | 0.999977 | AC011468.2 |
| ENSG00000244055.2 | 145.5197 | -0.38858 | 0.001417 | 0.999977 | AC007566.1 |
| ENSG00000231326.7 | 143.9229 | 0.358931 | 0.001676 | 0.999977 | LINC02662 |
| ENSG00000285999.1 | 51.54052 | 0.582007 | 0.002624 | 0.999977 | AC025442.2 |
| ENSG00000253182.1 | 109.8046 | 0.38182 | 0.003642 | 0.999977 | AC084026.1 |
| ENSG00000251364.7 | 154.2367 | 0.321855 | 0.004717 | 0.999977 | AC107884.1 |
| ENSG00000287707.1 | 55.88379 | -0.43246 | 0.005569 | 0.999977 | AC115284.4 |
| ENSG00000253281.7 | 83.20485 | -0.55246 | 0.006513 | 0.999977 | AC092819.1 |
| ENSG00000253894.2 | 52.58266 | 0.496893 | 0.006841 | 0.999977 | AC011124.2 |
| ENSG00000251562.8 | 367.0497 | 1.40435 | 0.007158 | 0.999977 | MALAT1 |
| ENSG00000238131.2 | 102.6305 | 0.512092 | 0.007268 | 0.999977 | LINC02854 |
| ENSG00000267772.1 | 191.8065 | -0.30696 | 0.008539 | 0.999977 | LINC01999 |
| ENSG00000231079.7 | 55.259 | -0.51652 | 0.008578 | 0.999977 | KIF5C-AS1 |
| ENSG00000286822.1 | 89.49292 | -0.34038 | 0.009182 | 0.999977 | AC112504.3 |
| ENSG00000226506.6 | 95.04816 | 0.340048 | 0.011056 | 0.999977 | AC007463.1 |
| ENSG00000257262.1 | 135.5361 | 0.350809 | 0.011639 | 0.999977 | AC023511.1 |
| ENSG00000249787.2 | 152.7029 | 0.372527 | 0.011823 | 0.999977 | AC113385.1 |
| ENSG00000231492.3 | 118.3748 | 0.308402 | 0.012003 | 0.999977 | AP003774.1 |
| ENSG00000258473.2 | 80.39489 | 0.339919 | 0.013054 | 0.999977 | AC007686.2 |
| ENSG00000255036.6 | 60.48473 | -0.49642 | 0.013259 | 0.999977 | SUGT1P4-STRA6LP-CCDC180 |
| ENSG00000228506.2 | 56.55874 | -0.38376 | 0.014514 | 0.999977 | AL513550.1 |
| ENSG00000240033.1 | 59.89615 | 0.430338 | 0.015474 | 0.999977 | AC069439.2 |
| ENSG00000230269.7 | 118.5833 | -0.36261 | 0.01584 | 0.999977 | LINC02525 |
| ENSG00000241048.1 | 109.4594 | -0.33785 | 0.017373 | 0.999977 | AC117386.1 |
| ENSG00000281128.2 | 63.1488 | -0.4896 | 0.019178 | 0.999977 | PTENP1-AS |
| ENSG00000260440.3 | 83.11316 | 0.319428 | 0.019723 | 0.999977 | LINC01544 |
| ENSG00000223631.2 | 50.72406 | -0.35539 | 0.020332 | 0.999977 | LINC01120 |
| ENSG00000240842.2 | 56.02386 | 0.408 | 0.023224 | 0.999977 | AC099328.2 |
| ENSG00000254007.2 | 72.36161 | 0.363107 | 0.023518 | 0.999977 | AC084768.1 |
| ENSG00000287183.1 | 74.35452 | 0.300329 | 0.023923 | 0.999977 | AC093824.2 |
| ENSG00000268416.1 | 64.16766 | -0.32178 | 0.024873 | 0.999977 | AC010329.1 |
| ENSG00000233017.3 | 252.7134 | -0.32042 | 0.024943 | 0.999977 | AL121832.1 |
| ENSG00000284722.2 | 81.03136 | 0.351885 | 0.024966 | 0.999977 | AP003175.1 |
| ENSG00000259723.1 | 86.50534 | -0.31843 | 0.025285 | 0.999977 | AL354993.2 |
| ENSG00000235295.1 | 75.87852 | -0.33418 | 0.025483 | 0.999977 | LINC01634 |
| ENSG00000227809.2 | 73.72173 | -0.36005 | 0.025682 | 0.999977 | AL355674.1 |
| ENSG00000258616.5 | 58.06184 | -0.43313 | 0.026173 | 0.999977 | LINC02303 |
| ENSG00000229109.2 | 107.8501 | -0.3291 | 0.026348 | 0.999977 | AL137847.1 |
| ENSG00000167920.10 | 74.76879 | -0.36508 | 0.028236 | 0.999977 | TMEM99 |
| ENSG00000276502.1 | 50.15944 | 0.372416 | 0.029086 | 0.999977 | AL354718.2 |
| ENSG00000185433.10 | 273.0733 | -0.32261 | 0.030828 | 0.999977 | LINC00158 |
| ENSG00000286010.1 | 69.79086 | -0.40806 | 0.030895 | 0.999977 | AC108925.1 |
| ENSG00000236354.1 | 67.64681 | -0.35807 | 0.030936 | 0.999977 | LINC00437 |
| ENSG00000285600.1 | 60.58786 | -0.3319 | 0.032763 | 0.999977 | AC023593.1 |
| ENSG00000239991.1 | 67.40084 | 0.319821 | 0.034476 | 0.999977 | AC092059.1 |
| ENSG00000287657.1 | 81.74437 | -0.34512 | 0.03489 | 0.999977 | AC068228.3 |
| ENSG00000267413.1 | 164.147 | -0.32765 | 0.035428 | 0.999977 | LINC01901 |
| ENSG00000267321.3 | 70.86617 | -0.37872 | 0.036207 | 0.999977 | SNHG30 |
| ENSG00000261729.2 | 82.32498 | 0.354219 | 0.039028 | 0.999977 | AL133383.1 |
| ENSG00000258649.1 | 54.73096 | -0.35871 | 0.043494 | 0.999977 | AL392023.1 |
| ENSG00000258140.2 | 91.20081 | -0.30479 | 0.043556 | 0.999977 | AC127894.1 |
| ENSG00000286901.1 | 61.28534 | -0.39812 | 0.045197 | 0.999977 | Z69667.1 |
| ENSG00000287746.1 | 59.28036 | -0.31027 | 0.046437 | 0.999977 | AL139035.2 |
| ENSG00000249099.1 | 75.07327 | 0.3676 | 0.048539 | 0.999977 | AC025475.1 |
| ENSG00000285159.1 | 59.44721 | 0.332922 | 0.049073 | 0.999977 | AL627422.2 |

**Table S7** The list of differentially expressed lncRNAs between stage II and stage III of CRC patients detected in serum sEVs.

| gene_id | baseMean | log2FoldChange | pvalue | padj | gene_name |
| --- | --- | --- | --- | --- | --- |
| ENSG00000255983.1 | 141.1068 | 0.313824 | 0.003 | 0.999954 | AC007848.1 |
| ENSG00000285872.1 | 143.7697 | -0.30305 | 0.00425 | 0.999954 | AC007240.3 |
| ENSG00000287182.1 | 58.61305 | -0.30865 | 0.005508 | 0.999954 | AC131953.2 |
| ENSG00000278996.1 | 495.7779 | -0.65765 | 0.006592 | 0.999954 | FP671120.4 |
| ENSG00000237101.1 | 71.08772 | 0.328986 | 0.0075 | 0.999954 | AC092809.4 |
| ENSG00000257221.4 | 98.20937 | -0.30333 | 0.008031 | 0.999954 | AC007569.1 |
| ENSG00000280916.2 | 90.16645 | -0.41378 | 0.010558 | 0.999954 | FOXCUT |
| ENSG00000245910.8 | 52.8829 | -0.34289 | 0.01244 | 0.999954 | SNHG6 |
| ENSG00000260887.3 | 75.51537 | 0.373882 | 0.012704 | 0.999954 | CASC22 |
| ENSG00000245532.9 | 252.2796 | -0.39534 | 0.012763 | 0.999954 | NEAT1 |
| ENSG00000286933.1 | 66.33217 | -0.32183 | 0.014501 | 0.999954 | AL022170.1 |
| ENSG00000258770.1 | 126.0313 | 0.322724 | 0.014542 | 0.999954 | LINC02330 |
| ENSG00000267095.1 | 55.09145 | -0.44411 | 0.016578 | 0.999954 | AC025048.1 |
| ENSG00000204860.5 | 54.17357 | -0.39003 | 0.017458 | 0.999954 | FAM201A |
| ENSG00000283999.1 | 60.58742 | 0.327878 | 0.017468 | 0.999954 | AL358215.3 |
| ENSG00000167459.17 | 88.52786 | -0.31397 | 0.020559 | 0.999954 | LINC00905 |
| ENSG00000261864.1 | 78.78476 | -0.3138 | 0.026997 | 0.999954 | AC130462.2 |
| ENSG00000197813.6 | 53.90991 | -0.38478 | 0.027371 | 0.999954 | AC011450.1 |
| ENSG00000232860.8 | 89.55553 | -0.31932 | 0.027499 | 0.999954 | SMG7-AS1 |
| ENSG00000235077.1 | 95.96083 | -0.32147 | 0.030499 | 0.999954 | AC073842.1 |
| ENSG00000260032.2 | 82.13245 | -0.70748 | 0.031241 | 0.999954 | NORAD |
| ENSG00000259478.2 | 63.48004 | 0.306107 | 0.032394 | 0.999954 | AC024651.1 |
| ENSG00000183250.12 | 99.10962 | -0.32773 | 0.033791 | 0.999954 | LINC01547 |
| ENSG00000226370.1 | 63.36664 | -0.36679 | 0.035739 | 0.999954 | LINC00375 |
| ENSG00000251562.8 | 367.0497 | -0.92911 | 0.036189 | 0.999954 | MALAT1 |
| ENSG00000225177.6 | 67.49785 | -0.35112 | 0.036979 | 0.999954 | AL590617.2 |
| ENSG00000288062.1 | 54.91375 | -0.36486 | 0.037419 | 0.999954 | AL136981.3 |
| ENSG00000231358.4 | 50.66795 | -0.3443 | 0.039477 | 0.999954 | AL355516.1 |
| ENSG00000284734.1 | 50.47672 | 0.346261 | 0.03958 | 0.999954 | AC099063.4 |
| ENSG00000267626.1 | 58.72486 | -0.31421 | 0.041485 | 0.999954 | AC002115.1 |
| ENSG00000257337.7 | 64.45057 | -0.32877 | 0.041822 | 0.999954 | AC068888.1 |
| ENSG00000285999.1 | 51.54052 | -0.33184 | 0.044042 | 0.999954 | AC025442.2 |
| ENSG00000243572.1 | 71.19046 | 0.344791 | 0.044176 | 0.999954 | LINC02017 |
| ENSG00000260362.1 | 75.86752 | 0.302938 | 0.049805 | 0.999954 | AC007218.1 |

**Table S8** The list of differentially expressed lncRNAs between stage II and stage IV of CRC patients detected in serum sEVs.

| gene_id | baseMean | log2FoldChange | pvalue | padj | gene_name |
| --- | --- | --- | --- | --- | --- |
| ENSG00000286562.1 | 82.95792 | -0.38544 | 0.001412 | 0.999984 | Z98742.4 |
| ENSG00000240040.6 | 252.389 | 0.302656 | 0.001476 | 0.999984 | AC244205.1 |
| ENSG00000260254.1 | 77.92449 | 0.484546 | 0.001593 | 0.999984 | AP000997.3 |
| ENSG00000235450.1 | 255.5118 | 0.355118 | 0.001678 | 0.999984 | AC079760.1 |
| ENSG00000272568.6 | 105.3916 | 0.328784 | 0.002351 | 0.999984 | AC005162.2 |
| ENSG00000268006.1 | 129.109 | -0.34117 | 0.002583 | 0.999984 | PTOV1-AS1 |
| ENSG00000231079.7 | 55.259 | -0.56836 | 0.002772 | 0.999984 | KIF5C-AS1 |
| ENSG00000267772.1 | 191.8065 | -0.33562 | 0.002919 | 0.999984 | LINC01999 |
| ENSG00000287183.1 | 74.35452 | 0.369445 | 0.00408 | 0.999984 | AC093824.2 |
| ENSG00000247363.2 | 80.96244 | -0.35667 | 0.004089 | 0.999984 | AC090061.1 |
| ENSG00000230641.1 | 62.79405 | -0.44104 | 0.004572 | 0.999984 | USP12-AS2 |
| ENSG00000226506.6 | 95.04816 | 0.3634 | 0.00501 | 0.999984 | AC007463.1 |
| ENSG00000270332.2 | 81.83514 | 0.521699 | 0.005314 | 0.999984 | SMC2-AS1 |
| ENSG00000227125.1 | 76.3346 | 0.430494 | 0.005514 | 0.999984 | AP002856.1 |
| ENSG00000263072.8 | 253.4104 | -0.33304 | 0.005905 | 0.999984 | ZNF213-AS1 |
| ENSG00000247796.3 | 121.0169 | 0.30846 | 0.006694 | 0.999984 | AC008966.1 |
| ENSG00000286821.1 | 128.5729 | 0.311148 | 0.0083 | 0.999984 | AC019193.4 |
| ENSG00000227888.4 | 78.90924 | 0.872239 | 0.008526 | 0.999984 | FAM66A |
| ENSG00000234177.5 | 101.8812 | 0.351377 | 0.00863 | 0.999984 | LINC01114 |
| ENSG00000231492.3 | 118.3748 | 0.30502 | 0.010251 | 0.999984 | AP003774.1 |
| ENSG00000256538.2 | 76.26682 | -0.45698 | 0.01063 | 0.999984 | AC046130.2 |
| ENSG00000284694.1 | 62.88296 | 0.480215 | 0.011949 | 0.999984 | AL355602.1 |
| ENSG00000239991.1 | 67.40084 | 0.36719 | 0.012082 | 0.999984 | AC092059.1 |
| ENSG00000282527.1 | 113.724 | -0.35244 | 0.012188 | 0.999984 | AC110491.3 |
| ENSG00000242578.1 | 68.22515 | -0.42184 | 0.014204 | 0.999984 | AC073288.2 |
| ENSG00000249725.1 | 131.4967 | 0.311852 | 0.014833 | 0.999984 | AC079942.1 |
| ENSG00000238171.1 | 53.17164 | -0.49344 | 0.015043 | 0.999984 | AC068196.1 |
| ENSG00000237523.2 | 85.56354 | 0.33285 | 0.015857 | 0.999984 | LINC00857 |
| ENSG00000277449.1 | 74.38753 | -0.40899 | 0.016156 | 0.999984 | CEBPB-AS1 |
| ENSG00000233017.3 | 252.7134 | -0.33147 | 0.016227 | 0.999984 | AL121832.1 |
| ENSG00000225102.2 | 68.74531 | -0.38868 | 0.016489 | 0.999984 | AL157373.1 |
| ENSG00000286621.1 | 56.83503 | -0.44272 | 0.017041 | 0.999984 | AC064843.1 |
| ENSG00000255036.6 | 60.48473 | -0.4614 | 0.017075 | 0.999984 | SUGT1P4-STRA6LP-CCDC180 |
| ENSG00000259478.2 | 63.48004 | 0.388995 | 0.017108 | 0.999984 | AC024651.1 |
| ENSG00000177410.13 | 168.698 | -0.31909 | 0.01721 | 0.999984 | ZFAS1 |
| ENSG00000286901.1 | 61.28534 | -0.45657 | 0.017499 | 0.999984 | Z69667.1 |
| ENSG00000260911.2 | 73.37264 | -0.34953 | 0.019854 | 0.999984 | AC135050.3 |
| ENSG00000253894.2 | 52.58266 | 0.413934 | 0.020161 | 0.999984 | AC011124.2 |
| ENSG00000287084.1 | 61.41895 | 0.36602 | 0.020167 | 0.999984 | AL355337.1 |
| ENSG00000284606.1 | 119.3198 | 0.32466 | 0.021543 | 0.999984 | AC105233.4 |
| ENSG00000257262.1 | 135.5361 | 0.306771 | 0.022538 | 0.999984 | AC023511.1 |
| ENSG00000269289.6 | 85.95789 | 0.338941 | 0.022592 | 0.999984 | AC011503.1 |
| ENSG00000287020.1 | 169.0001 | 0.381394 | 0.022942 | 0.999984 | AC091074.3 |
| ENSG00000237896.6 | 119.6368 | 0.388066 | 0.022951 | 0.999984 | AC005008.2 |
| ENSG00000287737.1 | 78.81244 | -0.36767 | 0.02372 | 0.999984 | AC132872.5 |
| ENSG00000253787.1 | 64.89887 | 0.384848 | 0.024397 | 0.999984 | LINC02219 |
| ENSG00000261864.1 | 78.78476 | -0.35616 | 0.026603 | 0.999984 | AC130462.2 |
| ENSG00000245864.3 | 140.3195 | -0.33217 | 0.026603 | 0.999984 | MEF2C-AS2 |
| ENSG00000231711.2 | 68.4021 | -0.38428 | 0.026662 | 0.999984 | LINC00899 |
| ENSG00000268945.1 | 55.77156 | -0.37592 | 0.02681 | 0.999984 | AC010422.2 |
| ENSG00000254055.2 | 119.2054 | -0.31033 | 0.0272 | 0.999984 | AC009597.1 |
| ENSG00000286933.1 | 66.33217 | -0.32729 | 0.028106 | 0.999984 | AL022170.1 |
| ENSG00000258586.1 | 70.62742 | -0.3308 | 0.028476 | 0.999984 | LINC02274 |
| ENSG00000259104.2 | 57.68388 | 0.402424 | 0.030266 | 0.999984 | PTCSC3 |
| ENSG00000253281.7 | 83.20485 | -0.42107 | 0.031429 | 0.999984 | AC092819.1 |
| ENSG00000238131.2 | 102.6305 | 0.394595 | 0.032438 | 0.999984 | LINC02854 |
| ENSG00000248533.1 | 80.76997 | -0.33943 | 0.035604 | 0.999984 | AC034226.1 |
| ENSG00000254007.2 | 72.36161 | 0.324265 | 0.036714 | 0.999984 | AC084768.1 |
| ENSG00000243572.1 | 71.19046 | 0.407301 | 0.036734 | 0.999984 | LINC02017 |
| ENSG00000228559.2 | 61.15981 | -0.35641 | 0.036951 | 0.999984 | AL033519.4 |
| ENSG00000285680.2 | 59.69578 | -0.42182 | 0.038058 | 0.999984 | AL355481.1 |
| ENSG00000248996.1 | 63.55184 | -0.40935 | 0.038613 | 0.999984 | AC145098.1 |
| ENSG00000267321.3 | 70.86617 | -0.35946 | 0.039507 | 0.999984 | SNHG30 |
| ENSG00000280916.2 | 90.16645 | -0.37625 | 0.040385 | 0.999984 | FOXCUT |
| ENSG00000267151.5 | 54.71381 | -0.30304 | 0.040386 | 0.999984 | MIR2117HG |
| ENSG00000275356.5 | 87.08294 | 0.366876 | 0.040673 | 0.999984 | C7orf77 |
| ENSG00000254321.2 | 55.75258 | 0.432746 | 0.042062 | 0.999984 | AC016813.1 |
| ENSG00000259772.6 | 81.48211 | -0.31199 | 0.043023 | 0.999984 | AC012236.1 |
| ENSG00000233695.2 | 55.16397 | -0.34502 | 0.043728 | 0.999984 | GAS6-AS1 |
| ENSG00000288024.1 | 96.04135 | 0.405743 | 0.04417 | 0.999984 | AC005301.2 |
| ENSG00000257379.1 | 60.38793 | -0.34897 | 0.04456 | 0.999984 | AC023509.1 |
| ENSG00000225177.6 | 67.49785 | -0.37961 | 0.046483 | 0.999984 | AL590617.2 |
| ENSG00000261729.2 | 82.32498 | 0.329849 | 0.046896 | 0.999984 | AL133383.1 |
| ENSG00000223711.1 | 63.34099 | -0.30511 | 0.046936 | 0.999984 | AC069213.1 |
| ENSG00000245928.2 | 78.41141 | 0.31845 | 0.0471 | 0.999984 | SDAD1-AS1 |
| ENSG00000268744.1 | 51.13378 | -0.30137 | 0.048627 | 0.999984 | AC008758.4 |
| ENSG00000284735.1 | 67.09675 | -0.36166 | 0.049526 | 0.999984 | AL139424.3 |

**Table S9** The list of differentially expressed lncRNAs between stage III and stage IV of CRC patients detected in serum sEVs.

| gene_id | baseMean | log2FoldChange | pvalue | padj | gene_name |
| --- | --- | --- | --- | --- | --- |
| ENSG00000244055.2 | 145.5197 | -0.44818 | 0.000213 | 0.999952 | AC007566.1 |
| ENSG00000286562.1 | 82.95792 | -0.45261 | 0.000276 | 0.999952 | Z98742.4 |
| ENSG00000231079.7 | 55.259 | -0.67968 | 0.000515 | 0.999952 | KIF5C-AS1 |
| ENSG00000248969.1 | 95.02306 | -0.36092 | 0.003028 | 0.999952 | AC137810.1 |
| ENSG00000258140.2 | 91.20081 | -0.41411 | 0.005856 | 0.999952 | AC127894.1 |
| ENSG00000246740.2 | 71.3414 | 0.35586 | 0.006022 | 0.999952 | PLA2G4E-AS1 |
| ENSG00000227713.2 | 57.89708 | -0.50884 | 0.006475 | 0.999952 | AC092159.1 |
| ENSG00000272568.6 | 105.3916 | 0.300516 | 0.006802 | 0.999952 | AC005162.2 |
| ENSG00000237396.1 | 156.5565 | -0.30924 | 0.007509 | 0.999952 | LNCNEF |
| ENSG00000237477.1 | 82.37774 | 0.359012 | 0.007624 | 0.999952 | AC093911.1 |
| ENSG00000287281.1 | 111.5609 | 0.385936 | 0.009258 | 0.999952 | AC012101.2 |
| ENSG00000253894.2 | 52.58266 | 0.466988 | 0.010568 | 0.999952 | AC011124.2 |
| ENSG00000260478.2 | 121.4832 | -0.45742 | 0.010888 | 0.999952 | AC007333.1 |
| ENSG00000255036.6 | 60.48473 | -0.49381 | 0.013045 | 0.999952 | SUGT1P4-STRA6LP-CCDC180 |
| ENSG00000230269.7 | 118.5833 | -0.37008 | 0.01309 | 0.999952 | LINC02525 |
| ENSG00000282527.1 | 113.724 | -0.35799 | 0.013226 | 0.999952 | AC110491.3 |
| ENSG00000228775.8 | 83.40945 | 0.356505 | 0.013924 | 0.999952 | WEE2-AS1 |
| ENSG00000223631.2 | 50.72406 | -0.36862 | 0.015658 | 0.999952 | LINC01120 |
| ENSG00000240842.2 | 56.02386 | 0.41684 | 0.019555 | 0.999952 | AC099328.2 |
| ENSG00000240152.2 | 54.73304 | -0.59098 | 0.019913 | 0.999952 | LINC02271 |
| ENSG00000235446.2 | 102.5074 | 0.376618 | 0.01997 | 0.999952 | LINC02791 |
| ENSG00000255269.3 | 74.78245 | -0.31214 | 0.020336 | 0.999952 | LINC02710 |
| ENSG00000287465.1 | 55.32885 | -0.50509 | 0.02183 | 0.999952 | AL358053.1 |
| ENSG00000236255.2 | 83.44994 | 0.33705 | 0.022108 | 0.999952 | AC009404.1 |
| ENSG00000225102.2 | 68.74531 | -0.38106 | 0.022161 | 0.999952 | AL157373.1 |
| ENSG00000287976.1 | 103.5247 | -0.31041 | 0.022453 | 0.999952 | AL445123.2 |
| ENSG00000287836.1 | 99.87729 | 0.377471 | 0.023094 | 0.999952 | AC016168.4 |
| ENSG00000246731.3 | 50.04076 | 0.490388 | 0.029633 | 0.999952 | MGC16275 |
| ENSG00000254055.2 | 119.2054 | -0.31231 | 0.030522 | 0.999952 | AC009597.1 |
| ENSG00000254192.1 | 64.7146 | -0.37164 | 0.030722 | 0.999952 | AC011365.1 |
| ENSG00000256695.2 | 78.07485 | 0.352959 | 0.030931 | 0.999952 | AC003982.1 |
| ENSG00000283999.1 | 60.58742 | -0.34333 | 0.032149 | 0.999952 | AL358215.3 |
| ENSG00000253177.2 | 79.24559 | -0.36577 | 0.032862 | 0.999952 | AC104211.1 |
| ENSG00000284694.1 | 62.88296 | 0.414621 | 0.034639 | 0.999952 | AL355602.1 |
| ENSG00000259269.2 | 154.2747 | -0.37583 | 0.035439 | 0.999952 | AC109630.1 |
| ENSG00000272815.1 | 92.64802 | 0.418997 | 0.037869 | 0.999952 | AC098850.4 |
| ENSG00000287189.1 | 68.668 | -0.3391 | 0.039522 | 0.999952 | AL121956.6 |
| ENSG00000287588.1 | 51.56328 | -0.33882 | 0.041308 | 0.999952 | AC092634.8 |
| ENSG00000233047.7 | 140.8937 | -0.30826 | 0.042352 | 0.999952 | LINC01677 |
| ENSG00000267199.1 | 74.31203 | 0.319308 | 0.044001 | 0.999952 | AP001029.2 |
| ENSG00000259772.6 | 81.48211 | -0.31886 | 0.044203 | 0.999952 | AC012236.1 |
| ENSG00000250024.1 | 76.36443 | -0.31411 | 0.045334 | 0.999952 | AC122138.1 |
| ENSG00000242770.2 | 63.41308 | 0.379292 | 0.046172 | 0.999952 | CD200R1L-AS1 |
| ENSG00000239628.1 | 72.76976 | -0.32611 | 0.0472 | 0.999952 | AC073288.1 |
| ENSG00000287349.1 | 68.64725 | -0.30756 | 0.04789 | 0.999952 | AC108477.2 |
| ENSG00000281128.2 | 63.1488 | -0.40747 | 0.049191 | 0.999952 | PTENP1-AS |
| ENSG00000265458.2 | 51.60769 | -0.38344 | 0.049894 | 0.999952 | AC132938.3 |

**Table S10** The list of differentially expressed lncRNAs between grade II and grade I of CRC patients detected in serum sEVs.

| gene_id | baseMean | log2FoldChange | pvalue | padj | gene_name |
| --- | --- | --- | --- | --- | --- |
| ENSG00000238131.2 | 103.9213 | 0.928048 | 1.29E-06 | 0.020604 | LINC02854 |
| ENSG00000254339.5 | 64.11535 | 0.744623 | 8.47E-06 | 0.040703 | AC064802.1 |
| ENSG00000281333.2 | 111.6421 | 0.874465 | 9.12E-06 | 0.040703 | AC024941.2 |
| ENSG00000260910.2 | 66.32979 | -0.78218 | 1.09E-05 | 0.040703 | LINC00565 |
| ENSG00000229401.1 | 163.3869 | 0.453901 | 1.15E-05 | 0.040703 | MIR5689HG |
| ENSG00000286439.1 | 59.4645 | 0.91468 | 4.32E-05 | 0.092011 | AL354916.1 |
| ENSG00000232193.1 | 90.8435 | 0.832965 | 6.23E-05 | 0.098439 | AL157359.2 |
| ENSG00000287364.1 | 71.19015 | 1.051344 | 7.49E-05 | 0.098439 | AL353709.1 |
| ENSG00000249171.1 | 359.9578 | 0.473786 | 9.85E-05 | 0.098439 | AC021192.1 |
| ENSG00000228679.1 | 137.27 | 0.564939 | 0.000106 | 0.098439 | AL034347.1 |
| ENSG00000230014.1 | 66.41013 | 0.747926 | 0.000113 | 0.098439 | LINC00709 |
| ENSG00000254444.1 | 414.259 | 0.335819 | 0.000148 | 0.108824 | AC022762.1 |
| ENSG00000272622.2 | 92.33298 | 0.711311 | 0.000186 | 0.116709 | AC010735.2 |
| ENSG00000235448.3 | 327.5333 | 0.598022 | 0.000218 | 0.125613 | LURAP1L-AS1 |
| ENSG00000232954.2 | 96.72258 | 0.779891 | 0.00022 | 0.125613 | LINC00374 |
| ENSG00000227075.1 | 212.6732 | 0.573807 | 0.000284 | 0.146291 | AP000472.1 |
| ENSG00000241383.2 | 185.3965 | 0.604933 | 0.000304 | 0.149296 | LINC01997 |
| ENSG00000287048.1 | 75.76512 | 0.828972 | 0.000312 | 0.149296 | AC096589.2 |
| ENSG00000287448.1 | 161.6254 | 0.536314 | 0.000328 | 0.149296 | AC018558.7 |
| ENSG00000258414.1 | 69.29947 | 0.705026 | 0.000393 | 0.155886 | AL121790.1 |
| ENSG00000251577.5 | 125.8266 | 0.710461 | 0.000405 | 0.155886 | AC105460.2 |
| ENSG00000268006.1 | 129.3558 | -0.42805 | 0.000408 | 0.155886 | PTOV1-AS1 |
| ENSG00000248837.7 | 1157.475 | 0.417567 | 0.000417 | 0.155886 | AC097512.1 |
| ENSG00000205056.8 | 50.09075 | 0.842352 | 0.000503 | 0.165733 | LINC02397 |
| ENSG00000258175.1 | 108.9367 | 0.743886 | 0.000566 | 0.166096 | LINC02300 |
| ENSG00000229246.2 | 141.0117 | 0.538785 | 0.000581 | 0.166096 | LINC00377 |
| ENSG00000241696.2 | 304.354 | 0.522233 | 0.000581 | 0.166096 | LINC02053 |
| ENSG00000248605.5 | 191.3566 | 0.629481 | 0.000603 | 0.166096 | AC022140.1 |
| ENSG00000253738.2 | 62.52917 | 0.73576 | 0.000689 | 0.175583 | OTUD6B-AS1 |
| ENSG00000285729.1 | 234.4711 | 0.345471 | 0.000702 | 0.176391 | AC103874.1 |
| ENSG00000197568.14 | 250.3547 | 0.372228 | 0.000707 | 0.176391 | HHLA3 |
| ENSG00000229395.1 | 80.64608 | 0.644307 | 0.000724 | 0.176583 | AC062039.1 |
| ENSG00000287854.1 | 80.22759 | 0.715407 | 0.000739 | 0.178875 | AC073409.2 |
| ENSG00000249996.1 | 110.0436 | 0.698794 | 0.000749 | 0.179338 | PPIC-AS1 |
| ENSG00000269966.1 | 81.43844 | 0.587367 | 0.000784 | 0.179338 | AL136164.2 |
| ENSG00000224851.2 | 181.5695 | 0.455827 | 0.000797 | 0.179338 | LINC00502 |
| ENSG00000244650.2 | 140.778 | 0.68292 | 0.00085 | 0.179338 | AC025566.1 |
| ENSG00000256193.6 | 124.5238 | 0.419343 | 0.000865 | 0.179338 | LINC00507 |
| ENSG00000125899.8 | 198.0014 | 0.49027 | 0.000882 | 0.179338 | LINC02871 |
| ENSG00000286023.1 | 263.9954 | 0.482854 | 0.001129 | 0.199611 | AP001924.1 |
| ENSG00000287708.1 | 481.9285 | 0.328494 | 0.001199 | 0.199611 | AL137017.1 |
| ENSG00000258028.3 | 798.8836 | 0.477897 | 0.001216 | 0.199695 | AL135878.1 |
| ENSG00000254380.2 | 289.8013 | 0.303643 | 0.001319 | 0.201171 | AC084734.1 |
| ENSG00000240006.1 | 73.04644 | -0.48684 | 0.001341 | 0.201171 | LINC02004 |
| ENSG00000286822.1 | 89.61895 | -0.43276 | 0.001353 | 0.201958 | AC112504.3 |
| ENSG00000258422.5 | 241.8795 | 0.34161 | 0.001381 | 0.204307 | AL160191.1 |
| ENSG00000262619.2 | 388.9531 | 0.314876 | 0.001412 | 0.204307 | LINC00621 |
| ENSG00000258107.3 | 139.8596 | 0.515186 | 0.001431 | 0.205089 | AL158058.1 |
| ENSG00000257585.1 | 375.5439 | 0.373707 | 0.001444 | 0.205089 | LINC00609 |
| ENSG00000226919.4 | 70.40867 | 0.516085 | 0.001445 | 0.205089 | AL365184.1 |
| ENSG00000286211.1 | 188.618 | 0.466209 | 0.001554 | 0.212088 | AC098817.1 |
| ENSG00000203565.3 | 161.6323 | 0.450634 | 0.001585 | 0.212088 | AL450313.1 |
| ENSG00000250846.6 | 144.6507 | 0.545448 | 0.001603 | 0.212088 | EPHA5-AS1 |
| ENSG00000269929.3 | 78.15981 | 0.591064 | 0.001611 | 0.212088 | MIRLET7A1HG |
| ENSG00000231674.1 | 197.162 | 0.547311 | 0.001613 | 0.212088 | LINC00410 |
| ENSG00000268744.1 | 51.76996 | -0.4927 | 0.00163 | 0.213414 | AC008758.4 |
| ENSG00000229459.1 | 221.1022 | 0.318633 | 0.001702 | 0.213522 | AC023669.1 |
| ENSG00000253949.1 | 157.6271 | 0.418007 | 0.001721 | 0.213522 | AC022634.2 |
| ENSG00000285737.1 | 204.1661 | 0.381305 | 0.001736 | 0.213522 | LINC02680 |
| ENSG00000226488.2 | 96.54467 | 0.493796 | 0.001787 | 0.216404 | LINC01824 |
| ENSG00000235079.1 | 91.01235 | 0.476424 | 0.00181 | 0.218249 | ZRANB2-AS1 |
| ENSG00000247373.3 | 108.6794 | -0.48248 | 0.001827 | 0.219327 | TMED2-DT |
| ENSG00000288062.1 | 54.43856 | -0.64566 | 0.001884 | 0.220823 | AL136981.3 |
| ENSG00000255193.1 | 168.6021 | 0.518636 | 0.001891 | 0.220823 | LINC02726 |
| ENSG00000280029.4 | 66.62378 | 0.630565 | 0.001905 | 0.220823 | AC244517.11 |
| ENSG00000261156.6 | 110.5939 | 0.376721 | 0.001988 | 0.224404 | LINC01989 |
| ENSG00000259724.2 | 249.5879 | 0.383347 | 0.002022 | 0.224993 | LINC01581 |
| ENSG00000285902.1 | 165.9413 | 0.43033 | 0.002043 | 0.224993 | AL136442.1 |
| ENSG00000286470.1 | 197.1286 | 0.432461 | 0.00211 | 0.228052 | AL034427.1 |
| ENSG00000234840.2 | 274.1165 | 0.453604 | 0.002145 | 0.228052 | LINC01239 |
| ENSG00000259129.6 | 165.7961 | 0.514916 | 0.002151 | 0.228052 | LINC00648 |
| ENSG00000234540.1 | 80.59104 | 0.600428 | 0.002173 | 0.228052 | AL080313.1 |
| ENSG00000237735.2 | 290.1835 | 0.516543 | 0.002202 | 0.228052 | AF130359.1 |
| ENSG00000224711.2 | 136.1991 | 0.546774 | 0.002225 | 0.228052 | LINC01706 |
| ENSG00000287001.1 | 122.7379 | -0.35584 | 0.002316 | 0.230504 | AC010624.5 |
| ENSG00000282381.1 | 234.7886 | 0.357855 | 0.002367 | 0.230504 | AC104073.4 |
| ENSG00000285568.1 | 136.8645 | 0.436998 | 0.002427 | 0.230504 | AP003398.2 |
| ENSG00000227902.2 | 88.48223 | 0.631454 | 0.002432 | 0.230504 | AC062032.1 |
| ENSG00000287989.1 | 385.1417 | 0.434938 | 0.002434 | 0.230504 | AL450352.1 |
| ENSG00000251055.2 | 70.70783 | 0.684392 | 0.002434 | 0.230504 | AC097491.1 |
| ENSG00000287499.1 | 95.22457 | 0.655975 | 0.002453 | 0.230504 | AL357139.2 |
| ENSG00000244358.1 | 99.49408 | 0.66352 | 0.00254 | 0.23436 | AC055758.2 |
| ENSG00000248801.7 | 179.7064 | 0.331412 | 0.002573 | 0.235222 | C8orf34-AS1 |
| ENSG00000250488.2 | 587.2884 | 0.389274 | 0.002621 | 0.236197 | LINC02233 |
| ENSG00000235815.1 | 64.30501 | -0.58366 | 0.0027 | 0.236197 | AL136099.1 |
| ENSG00000259230.1 | 139.1275 | -0.41485 | 0.002724 | 0.237084 | LINC02323 |
| ENSG00000253891.2 | 52.18416 | 0.602418 | 0.00274 | 0.237554 | AC023202.1 |
| ENSG00000236013.7 | 599.6515 | 0.381218 | 0.002779 | 0.238189 | AL357146.1 |
| ENSG00000251088.1 | 1191.364 | 0.314617 | 0.00278 | 0.238189 | AC117473.1 |
| ENSG00000254363.6 | 141.7239 | -0.32147 | 0.002934 | 0.243476 | AC011379.2 |
| ENSG00000287372.1 | 180.987 | 0.431136 | 0.003105 | 0.247078 | AL627316.1 |
| ENSG00000266258.1 | 86.89157 | 0.407294 | 0.003126 | 0.247199 | LINC01909 |
| ENSG00000285998.1 | 60.69002 | 0.627992 | 0.003244 | 0.253847 | AC104790.1 |
| ENSG00000281641.3 | 1636.822 | 0.301866 | 0.003289 | 0.254363 | SAMD12-AS1 |
| ENSG00000255470.1 | 235.9886 | 0.379387 | 0.003562 | 0.262363 | AC090099.1 |
| ENSG00000226566.2 | 340.0382 | 0.49369 | 0.003595 | 0.262363 | AL390962.1 |
| ENSG00000231364.3 | 130.782 | 0.507763 | 0.003811 | 0.265916 | LINC01712 |
| ENSG00000251266.2 | 277.4984 | 0.499217 | 0.003819 | 0.265916 | LINC02429 |
| ENSG00000286827.1 | 333.4682 | 0.381386 | 0.003835 | 0.265916 | AC072026.2 |
| ENSG00000246366.6 | 187.2841 | 0.316805 | 0.003874 | 0.266419 | LACTB2-AS1 |
| ENSG00000250038.7 | 305.727 | 0.503588 | 0.003883 | 0.266419 | AC093791.1 |
| ENSG00000228506.2 | 57.21984 | -0.47664 | 0.0039 | 0.266792 | AL513550.1 |
| ENSG00000235884.4 | 99.17776 | 0.447994 | 0.003963 | 0.269897 | LINC00941 |
| ENSG00000283897.2 | 105.4855 | 0.366986 | 0.003994 | 0.270796 | AC011416.3 |
| ENSG00000249742.2 | 172.7597 | 0.386653 | 0.00401 | 0.270796 | AC110772.1 |
| ENSG00000253956.1 | 69.08161 | 0.699095 | 0.004101 | 0.271864 | AC073023.1 |
| ENSG00000254768.6 | 292.6015 | 0.420977 | 0.00412 | 0.272377 | AC104009.1 |
| ENSG00000238265.1 | 73.79212 | 0.647375 | 0.004145 | 0.272377 | LINC00317 |
| ENSG00000233296.2 | 154.6419 | 0.376092 | 0.00419 | 0.273588 | TMEM18-DT |
| ENSG00000238062.6 | 77.57182 | -0.5257 | 0.00424 | 0.273918 | SPATA3-AS1 |
| ENSG00000233930.4 | 272.7179 | -0.36156 | 0.004278 | 0.274979 | KRTAP5-AS1 |
| ENSG00000233960.1 | 162.3564 | 0.353074 | 0.004446 | 0.278331 | AC079763.1 |
| ENSG00000254334.2 | 66.43236 | 0.627988 | 0.004471 | 0.278331 | AC021355.1 |
| ENSG00000258084.6 | 380.307 | 0.497353 | 0.004534 | 0.279552 | AC128707.1 |
| ENSG00000286778.1 | 221.0417 | 0.493668 | 0.004561 | 0.279552 | AC013265.1 |
| ENSG00000233304.7 | 106.5984 | -0.37255 | 0.004635 | 0.279552 | LINC01346 |
| ENSG00000257262.1 | 135.5916 | 0.441395 | 0.004674 | 0.279552 | AC023511.1 |
| ENSG00000287485.1 | 162.3229 | 0.348756 | 0.004737 | 0.279552 | AL451166.1 |
| ENSG00000248744.1 | 191.1878 | 0.482966 | 0.004949 | 0.282828 | AC108467.1 |
| ENSG00000230269.7 | 118.0756 | -0.46454 | 0.005018 | 0.282859 | LINC02525 |
| ENSG00000267686.2 | 205.7475 | 0.419555 | 0.00502 | 0.282859 | AC090771.2 |
| ENSG00000259336.1 | 397.3825 | 0.382551 | 0.005074 | 0.28296 | AC021231.1 |
| ENSG00000286197.1 | 196.5203 | 0.340576 | 0.005145 | 0.28296 | AC084364.3 |
| ENSG00000287566.1 | 62.79337 | -0.62168 | 0.005285 | 0.286665 | AL732437.3 |
| ENSG00000286809.1 | 92.92308 | 0.435751 | 0.005286 | 0.286665 | AC008964.1 |
| ENSG00000280441.3 | 258.6878 | 1.345882 | 0.005386 | 0.287801 | FP236383.3 |
| ENSG00000286476.1 | 173.1621 | 0.496045 | 0.005411 | 0.287801 | AC079030.1 |
| ENSG00000261446.4 | 315.4502 | 0.385294 | 0.005449 | 0.287801 | LINC00559 |
| ENSG00000244198.7 | 56.95571 | 0.579153 | 0.005453 | 0.287801 | ARHGEF35-AS1 |
| ENSG00000236921.1 | 50.82583 | 0.657815 | 0.005474 | 0.287988 | AL157937.1 |
| ENSG00000248939.1 | 499.7373 | 0.418413 | 0.005534 | 0.289311 | AC111198.1 |
| ENSG00000258616.5 | 61.07581 | 0.61237 | 0.005568 | 0.290634 | LINC02303 |
| ENSG00000234660.2 | 387.3606 | 0.450176 | 0.005652 | 0.291208 | LINC00440 |
| ENSG00000248148.1 | 310.58 | 0.5292 | 0.005742 | 0.294405 | AC114954.1 |
| ENSG00000253164.5 | 198.8508 | -0.32998 | 0.00577 | 0.29457 | AC023403.1 |
| ENSG00000287976.1 | 104.7143 | 0.412584 | 0.005952 | 0.2953 | AL445123.2 |
| ENSG00000286891.1 | 1089.655 | 0.429223 | 0.00604 | 0.29776 | AC080132.1 |
| ENSG00000254202.2 | 1173.168 | 0.344393 | 0.006096 | 0.298616 | AC015522.1 |
| ENSG00000259072.2 | 624.8882 | 0.421475 | 0.006148 | 0.29902 | AL355835.1 |
| ENSG00000248397.1 | 65.0324 | 0.543864 | 0.006232 | 0.300668 | LINC00498 |
| ENSG00000256128.6 | 164.2418 | 0.429068 | 0.006293 | 0.300668 | LINC00944 |
| ENSG00000235885.8 | 186.6035 | 0.489939 | 0.006443 | 0.300668 | LINC01828 |
| ENSG00000236769.3 | 66.65479 | -0.45181 | 0.006469 | 0.300815 | LINC02659 |
| ENSG00000232656.9 | 118.5129 | -0.32301 | 0.006634 | 0.304732 | IDI2-AS1 |
| ENSG00000237896.6 | 120.8401 | 0.505015 | 0.006639 | 0.304732 | AC005008.2 |
| ENSG00000248858.7 | 454.0268 | 0.394458 | 0.006751 | 0.305931 | FLJ46284 |
| ENSG00000287474.1 | 2050.048 | 0.35265 | 0.006816 | 0.307111 | AC110614.1 |
| ENSG00000258586.1 | 72.50213 | -0.47871 | 0.006847 | 0.307545 | LINC02274 |
| ENSG00000253500.6 | 699.2812 | 0.347095 | 0.006855 | 0.307545 | AF121898.1 |
| ENSG00000254115.1 | 106.0048 | 0.491462 | 0.007074 | 0.309769 | AC090572.3 |
| ENSG00000250334.6 | 696.8395 | 0.403956 | 0.007076 | 0.309769 | LINC00989 |
| ENSG00000286377.1 | 414.397 | 0.346737 | 0.00713 | 0.31117 | AC114964.2 |
| ENSG00000248227.1 | 132.6482 | 0.403766 | 0.00725 | 0.312133 | LINC02513 |
| ENSG00000249096.7 | 138.3586 | -0.30786 | 0.007277 | 0.312877 | LINC02362 |
| ENSG00000287083.1 | 707.6899 | 0.386747 | 0.007352 | 0.313078 | AC108169.1 |
| ENSG00000232130.1 | 139.6866 | 0.43676 | 0.007406 | 0.313078 | AC092966.1 |
| ENSG00000262728.6 | 50.42929 | 0.542732 | 0.007522 | 0.313078 | AC123768.2 |
| ENSG00000237552.1 | 132.4162 | 0.412647 | 0.00758 | 0.313078 | LINC02567 |
| ENSG00000273507.5 | 963.5194 | 0.303491 | 0.007624 | 0.313078 | AL354809.1 |
| ENSG00000243550.3 | 183.7964 | 0.382825 | 0.007844 | 0.313078 | LINC01214 |
| ENSG00000265374.1 | 134.4702 | 0.399615 | 0.008068 | 0.315124 | LINC01908 |
| ENSG00000235221.3 | 497.0813 | 0.468572 | 0.00812 | 0.316067 | LINC00383 |
| ENSG00000226125.2 | 108.1157 | -0.38992 | 0.008293 | 0.317081 | LINC01907 |
| ENSG00000223470.3 | 57.13077 | 0.439688 | 0.008303 | 0.317081 | LINC02629 |
| ENSG00000257747.2 | 197.5011 | 0.445281 | 0.008331 | 0.317081 | LINC02426 |
| ENSG00000287008.1 | 265.9738 | 0.368631 | 0.008386 | 0.317631 | AC099563.2 |
| ENSG00000264260.2 | 89.77337 | -0.35957 | 0.008468 | 0.318994 | LINC01893 |
| ENSG00000267772.1 | 192.6888 | -0.33078 | 0.008553 | 0.319791 | LINC01999 |
| ENSG00000258743.6 | 530.1885 | 0.420047 | 0.00862 | 0.32099 | LINC02301 |
| ENSG00000287980.1 | 126.5441 | 0.451769 | 0.008672 | 0.32099 | AL391557.1 |
| ENSG00000287038.1 | 77.74235 | 0.493572 | 0.008791 | 0.32099 | AL162388.2 |
| ENSG00000286082.1 | 208.558 | -0.34356 | 0.008791 | 0.32099 | AL133493.1 |
| ENSG00000253796.2 | 1081.522 | 0.307202 | 0.0088 | 0.32099 | AC104248.1 |
| ENSG00000244278.1 | 71.06749 | 0.579168 | 0.00887 | 0.321988 | AP000235.1 |
| ENSG00000251538.7 | 611.6916 | 0.313448 | 0.009006 | 0.322162 | LINC02201 |
| ENSG00000284735.1 | 66.1141 | -0.55563 | 0.009051 | 0.323145 | AL139424.3 |
| ENSG00000287907.1 | 1257.854 | 0.350129 | 0.009104 | 0.323492 | AC005909.2 |
| ENSG00000163597.15 | 58.86931 | -0.48634 | 0.009422 | 0.326637 | SNHG16 |
| ENSG00000286820.1 | 61.23413 | 0.640058 | 0.009452 | 0.326752 | AL590027.1 |
| ENSG00000223812.8 | 901.3967 | 0.343621 | 0.009479 | 0.327004 | AC073365.1 |
| ENSG00000266950.1 | 101.9811 | -0.35039 | 0.009525 | 0.327403 | AC008752.1 |
| ENSG00000288024.1 | 98.62125 | 0.565185 | 0.009593 | 0.327403 | AC005301.2 |
| ENSG00000180869.4 | 58.39345 | 0.572054 | 0.009635 | 0.328498 | LINC01555 |
| ENSG00000286421.1 | 53.16181 | 0.656985 | 0.009796 | 0.330393 | AL360013.4 |
| ENSG00000287876.1 | 1059.475 | 0.381874 | 0.010015 | 0.331948 | AL512452.1 |
| ENSG00000259639.6 | 1057.386 | 0.424954 | 0.010222 | 0.331948 | AC021351.1 |
| ENSG00000236166.1 | 67.99636 | 0.535439 | 0.010291 | 0.331948 | AL021408.1 |
| ENSG00000237527.1 | 147.2717 | 0.456327 | 0.010553 | 0.331948 | AF241725.1 |
| ENSG00000226067.7 | 176.8123 | 0.329156 | 0.01065 | 0.331948 | LINC00623 |
| ENSG00000225721.5 | 81.11867 | -0.44588 | 0.010652 | 0.331948 | AL592166.1 |
| ENSG00000235774.2 | 217.2262 | 0.333481 | 0.010699 | 0.331948 | AC023347.1 |
| ENSG00000248238.2 | 1470.864 | 0.356803 | 0.010738 | 0.331948 | LINC02438 |
| ENSG00000275356.5 | 86.77948 | 0.600698 | 0.010834 | 0.331948 | C7orf77 |
| ENSG00000281207.1 | 55.54315 | -0.44657 | 0.010895 | 0.331948 | SLFNL1-AS1 |
| ENSG00000269834.6 | 92.3906 | -0.38049 | 0.010954 | 0.332058 | ZNF528-AS1 |
| ENSG00000261761.5 | 128.5449 | 0.411664 | 0.011056 | 0.332754 | LINC02616 |
| ENSG00000250863.2 | 242.9819 | 0.518017 | 0.011234 | 0.332754 | AC114757.1 |
| ENSG00000259104.2 | 59.8877 | 0.553992 | 0.011273 | 0.332754 | PTCSC3 |
| ENSG00000223811.1 | 64.99191 | 0.484016 | 0.011373 | 0.332754 | AL589684.1 |
| ENSG00000248708.2 | 154.7601 | 0.44068 | 0.011623 | 0.334557 | LINC02144 |
| ENSG00000253287.1 | 68.2513 | 0.539821 | 0.011652 | 0.334557 | AC104012.1 |
| ENSG00000255814.2 | 327.766 | 0.312411 | 0.011758 | 0.334557 | LINC02439 |
| ENSG00000286647.1 | 74.89058 | 0.421099 | 0.011889 | 0.334557 | AC008459.1 |
| ENSG00000250437.1 | 495.8845 | 0.325892 | 0.012004 | 0.335479 | LINC02161 |
| ENSG00000253103.2 | 698.5574 | 0.366317 | 0.01203 | 0.335631 | LINC01609 |
| ENSG00000286580.1 | 81.52278 | 0.510241 | 0.012044 | 0.335716 | AC034159.2 |
| ENSG00000248131.6 | 1171.336 | 0.331455 | 0.012172 | 0.336374 | LINC01194 |
| ENSG00000260983.2 | 180.784 | 0.337598 | 0.01226 | 0.337636 | AC010528.1 |
| ENSG00000254562.7 | 197.7859 | 0.467554 | 0.01231 | 0.337822 | LINC01493 |
| ENSG00000285961.1 | 894.3696 | 0.399548 | 0.012331 | 0.337822 | AL590814.1 |
| ENSG00000226091.7 | 197.4463 | -0.327 | 0.01242 | 0.338169 | LINC00937 |
| ENSG00000230142.2 | 190.2591 | 0.407938 | 0.0127 | 0.338801 | LINC01075 |
| ENSG00000251182.2 | 256.2764 | 0.342545 | 0.012725 | 0.338801 | LINC02497 |
| ENSG00000250436.1 | 142.1294 | 0.363553 | 0.01276 | 0.338826 | LINC02499 |
| ENSG00000234752.1 | 91.85059 | 0.456385 | 0.012777 | 0.338826 | LINC02676 |
| ENSG00000253108.1 | 541.4946 | 0.34023 | 0.012945 | 0.339822 | AC090993.1 |
| ENSG00000255774.2 | 85.44002 | -0.5139 | 0.013089 | 0.339822 | LINC02747 |
| ENSG00000231231.5 | 92.09681 | -0.38907 | 0.013167 | 0.339822 | LINC01423 |
| ENSG00000286348.1 | 112.4413 | -0.39769 | 0.013239 | 0.339968 | AC084834.1 |
| ENSG00000236780.7 | 487.6347 | 0.308496 | 0.013277 | 0.339968 | LINC01829 |
| ENSG00000251388.1 | 717.0621 | 0.379391 | 0.013344 | 0.340169 | AC079380.1 |
| ENSG00000227542.1 | 123.7314 | 0.428774 | 0.013507 | 0.340714 | AC092614.1 |
| ENSG00000228215.3 | 807.9072 | 0.41729 | 0.013532 | 0.340714 | LINC02770 |
| ENSG00000254854.1 | 102.5125 | -0.33819 | 0.013612 | 0.341021 | AP003390.1 |
| ENSG00000286605.1 | 649.4178 | 0.391986 | 0.013828 | 0.341022 | AC115100.1 |
| ENSG00000280752.1 | 263.4838 | 0.48977 | 0.013905 | 0.341022 | LINC00850 |
| ENSG00000266913.1 | 639.4292 | -0.3025 | 0.014006 | 0.341949 | LINC01841 |
| ENSG00000235450.1 | 258.4886 | 0.306475 | 0.01403 | 0.341949 | AC079760.1 |
| ENSG00000186235.11 | 99.16497 | -0.41557 | 0.014056 | 0.341949 | LINC02610 |
| ENSG00000258751.2 | 470.4835 | 0.425975 | 0.014062 | 0.341949 | AL358335.2 |
| ENSG00000261313.2 | 132.4431 | 0.323701 | 0.014098 | 0.341949 | AC105430.1 |
| ENSG00000205300.3 | 61.5155 | -0.53941 | 0.014147 | 0.342627 | AL356414.1 |
| ENSG00000251680.6 | 814.0826 | 0.32662 | 0.014384 | 0.343479 | AC008591.1 |
| ENSG00000285844.2 | 66.06854 | 0.321212 | 0.014432 | 0.343479 | FO393414.3 |
| ENSG00000258123.1 | 228.2207 | 0.364442 | 0.014721 | 0.343479 | LINC02444 |
| ENSG00000231424.3 | 1581.901 | 0.324478 | 0.014816 | 0.343479 | BX284613.2 |
| ENSG00000259805.1 | 60.15757 | -0.3863 | 0.014827 | 0.343479 | AC022558.2 |
| ENSG00000228400.2 | 81.33104 | 0.451389 | 0.014959 | 0.343864 | AC079154.1 |
| ENSG00000267786.1 | 166.4613 | -0.43539 | 0.015026 | 0.343864 | AF038458.3 |
| ENSG00000264254.1 | 51.70928 | 0.451898 | 0.015038 | 0.343864 | AP001496.1 |
| ENSG00000237283.2 | 137.8716 | 0.523668 | 0.01512 | 0.34526 | AL691515.1 |
| ENSG00000272243.6 | 192.0379 | 0.475663 | 0.015157 | 0.345858 | AL356277.3 |
| ENSG00000227418.6 | 133.0163 | 0.481918 | 0.015259 | 0.347221 | PCGEM1 |
| ENSG00000285796.1 | 101.9736 | -0.37922 | 0.015282 | 0.347221 | AL162458.1 |
| ENSG00000285744.1 | 779.3036 | 0.327702 | 0.015369 | 0.348019 | AC083837.1 |
| ENSG00000254180.2 | 128.3944 | 0.417891 | 0.01551 | 0.349039 | AC004083.1 |
| ENSG00000249942.1 | 184.0692 | 0.373985 | 0.015515 | 0.349039 | AC239584.1 |
| ENSG00000258119.1 | 253.1862 | 0.409056 | 0.015559 | 0.349276 | AC087897.2 |
| ENSG00000224445.3 | 710.5316 | 0.326341 | 0.015691 | 0.349789 | LINC01708 |
| ENSG00000223563.1 | 155.3367 | 0.433403 | 0.015895 | 0.351633 | AP001599.1 |
| ENSG00000239572.3 | 813.4959 | 0.303349 | 0.016036 | 0.353476 | AC108749.1 |
| ENSG00000223685.6 | 1450.638 | 0.339915 | 0.016078 | 0.353476 | LINC00571 |
| ENSG00000223479.3 | 402.2771 | 0.395741 | 0.016259 | 0.354073 | LINC02238 |
| ENSG00000242339.2 | 67.20489 | 0.463249 | 0.016512 | 0.356644 | LINC02025 |
| ENSG00000259111.1 | 74.3831 | -0.35075 | 0.016598 | 0.356828 | AL079307.2 |
| ENSG00000286182.1 | 136.8943 | 0.495176 | 0.01667 | 0.356828 | AC112172.2 |
| ENSG00000232229.6 | 65.80777 | -0.45019 | 0.016956 | 0.358033 | LINC00865 |
| ENSG00000253973.3 | 136.1616 | -0.34426 | 0.016979 | 0.358033 | AC079296.1 |
| ENSG00000259989.1 | 162.6911 | -0.42139 | 0.017045 | 0.358033 | AC135782.2 |
| ENSG00000226733.4 | 426.5291 | 0.471309 | 0.017105 | 0.358033 | AL138826.1 |
| ENSG00000257750.2 | 300.4714 | 0.496899 | 0.017145 | 0.358033 | LINC02445 |
| ENSG00000223711.1 | 63.75794 | -0.39679 | 0.017378 | 0.358033 | AC069213.1 |
| ENSG00000251471.1 | 137.5998 | 0.303041 | 0.017405 | 0.358033 | AC016933.1 |
| ENSG00000232606.2 | 325.0886 | 0.349439 | 0.017422 | 0.358033 | LINC01412 |
| ENSG00000267506.5 | 138.3212 | -0.31889 | 0.017463 | 0.358033 | AC021683.2 |
| ENSG00000257434.1 | 107.1878 | 0.506949 | 0.017471 | 0.358033 | AC073525.1 |
| ENSG00000286784.1 | 873.3815 | 0.344388 | 0.017512 | 0.358033 | AC116611.1 |
| ENSG00000285872.1 | 143.9298 | -0.31358 | 0.017631 | 0.358033 | AC007240.3 |
| ENSG00000241098.1 | 238.2239 | 0.34176 | 0.017742 | 0.358033 | LINC01994 |
| ENSG00000227432.1 | 89.6168 | -0.44575 | 0.017836 | 0.358312 | ASIC4-AS1 |
| ENSG00000255135.4 | 82.73226 | 0.44249 | 0.01788 | 0.358312 | AP002360.1 |
| ENSG00000285804.2 | 125.2883 | 0.328085 | 0.018185 | 0.35958 | AC025774.1 |
| ENSG00000233607.1 | 547.8546 | 0.321421 | 0.018189 | 0.35958 | LINC01392 |
| ENSG00000287629.1 | 134.5274 | -0.30417 | 0.018283 | 0.35958 | AC006059.5 |
| ENSG00000224559.2 | 64.56285 | 0.469807 | 0.018346 | 0.35958 | LINC01087 |
| ENSG00000237844.2 | 3229.405 | 0.33047 | 0.018364 | 0.35958 | AC016766.1 |
| ENSG00000232077.2 | 377.7424 | 0.373343 | 0.018381 | 0.35958 | LINC01031 |
| ENSG00000256508.2 | 64.50823 | -0.44404 | 0.018404 | 0.35958 | MRGPRF-AS1 |
| ENSG00000264701.1 | 168.1619 | -0.30609 | 0.018777 | 0.361739 | AC018521.4 |
| ENSG00000230962.2 | 248.1907 | 0.353641 | 0.018809 | 0.361739 | LINC01520 |
| ENSG00000236678.8 | 60.00063 | 0.566872 | 0.01883 | 0.361739 | LINC00347 |
| ENSG00000255102.1 | 213.0465 | 0.319625 | 0.01884 | 0.361739 | AP005436.1 |
| ENSG00000237612.1 | 149.0369 | -0.3106 | 0.018876 | 0.361739 | AP002856.4 |
| ENSG00000249618.6 | 994.2538 | 0.387723 | 0.018908 | 0.361739 | LINC02465 |
| ENSG00000287211.1 | 151.0173 | 0.506171 | 0.019383 | 0.363808 | AL121904.2 |
| ENSG00000251183.1 | 86.52704 | 0.392197 | 0.019488 | 0.363856 | LINC01861 |
| ENSG00000239628.1 | 72.5546 | -0.41356 | 0.019812 | 0.366358 | AC073288.1 |
| ENSG00000285782.2 | 98.63706 | 0.420769 | 0.019948 | 0.366358 | AC116099.1 |
| ENSG00000249203.2 | 852.2474 | 0.351635 | 0.02004 | 0.366358 | LINC02224 |
| ENSG00000287910.1 | 80.84912 | -0.34409 | 0.020197 | 0.366358 | AC104024.4 |
| ENSG00000286060.1 | 122.4796 | 0.539076 | 0.020257 | 0.366358 | AL365256.1 |
| ENSG00000287389.1 | 112.6511 | 0.555326 | 0.020377 | 0.366358 | AC007106.2 |
| ENSG00000259093.2 | 312.1175 | 0.320097 | 0.020396 | 0.366358 | AL137191.1 |
| ENSG00000227143.1 | 51.6145 | 0.422618 | 0.020473 | 0.366358 | LINC01153 |
| ENSG00000258081.4 | 1725.148 | 0.401094 | 0.020493 | 0.366358 | AL110292.1 |
| ENSG00000260975.1 | 172.0585 | 0.308092 | 0.020561 | 0.366358 | AC007333.2 |
| ENSG00000278305.1 | 128.6702 | 0.399032 | 0.020566 | 0.366358 | AL161616.2 |
| ENSG00000254594.5 | 117.9978 | 0.537327 | 0.020931 | 0.366358 | LINC02686 |
| ENSG00000204460.3 | 216.7206 | 0.326824 | 0.021297 | 0.366358 | LINC01854 |
| ENSG00000280683.1 | 874.605 | 0.330979 | 0.021492 | 0.366682 | LINC01242 |
| ENSG00000287968.1 | 301.3621 | 0.379357 | 0.021538 | 0.366682 | AC093606.1 |
| ENSG00000286530.1 | 79.24724 | 0.532051 | 0.021707 | 0.367861 | AC108057.1 |
| ENSG00000261864.1 | 77.27002 | -0.49062 | 0.021865 | 0.368389 | AC130462.2 |
| ENSG00000232347.1 | 178.9339 | 0.302173 | 0.022191 | 0.368836 | AL390728.4 |
| ENSG00000168367.11 | 163.0976 | -0.30583 | 0.022287 | 0.368836 | LINC00917 |
| ENSG00000238164.6 | 68.37845 | -0.45067 | 0.022395 | 0.368836 | TNFRSF14-AS1 |
| ENSG00000287605.1 | 60.4852 | 0.480201 | 0.0224 | 0.368836 | AC109131.1 |
| ENSG00000286383.1 | 100.9212 | 0.356034 | 0.022553 | 0.368836 | AC096645.1 |
| ENSG00000269994.3 | 199.6793 | 0.324449 | 0.023021 | 0.371205 | AL513318.1 |
| ENSG00000258416.1 | 443.4009 | 0.300231 | 0.023063 | 0.371205 | AF123462.1 |
| ENSG00000255983.1 | 143.8799 | 0.328288 | 0.023216 | 0.37136 | AC007848.1 |
| ENSG00000281167.1 | 578.7129 | 0.322727 | 0.02324 | 0.37136 | AL355390.2 |
| ENSG00000253214.2 | 127.7786 | 0.359727 | 0.023435 | 0.371756 | AC079209.1 |
| ENSG00000249413.2 | 666.6074 | 0.349651 | 0.023524 | 0.371756 | AC116049.1 |
| ENSG00000287398.1 | 147.6552 | 0.307916 | 0.023548 | 0.371756 | AC117481.1 |
| ENSG00000267577.1 | 51.72573 | -0.40124 | 0.023724 | 0.372976 | AC010327.4 |
| ENSG00000287020.1 | 171.214 | 0.410845 | 0.024117 | 0.375292 | AC091074.3 |
| ENSG00000287451.1 | 530.0739 | 0.312631 | 0.024128 | 0.375292 | AC016721.1 |
| ENSG00000225546.6 | 1928.237 | 0.379692 | 0.024223 | 0.375397 | LINC02476 |
| ENSG00000257373.1 | 110.8058 | 0.356689 | 0.024239 | 0.375397 | AC012038.1 |
| ENSG00000286694.1 | 115.7282 | -0.30067 | 0.024313 | 0.375397 | AL161935.3 |
| ENSG00000288537.1 | 53.95056 | -0.37716 | 0.02441 | 0.375397 | AL162391.2 |
| ENSG00000272154.5 | 126.7888 | 0.428118 | 0.024481 | 0.375397 | AC244517.2 |
| ENSG00000241328.1 | 63.6666 | 0.532206 | 0.024639 | 0.375397 | LINC02070 |
| ENSG00000224027.1 | 101.7344 | -0.33292 | 0.025008 | 0.375739 | AL133456.1 |
| ENSG00000235099.1 | 162.8447 | 0.479 | 0.02514 | 0.37593 | AL138731.1 |
| ENSG00000287158.1 | 471.0728 | 0.375313 | 0.025195 | 0.37593 | AC117464.1 |
| ENSG00000251632.1 | 283.1058 | 0.381428 | 0.025242 | 0.37593 | LINC02172 |
| ENSG00000286386.1 | 93.69666 | 0.391749 | 0.02557 | 0.376454 | AC005888.1 |
| ENSG00000275239.4 | 99.5171 | -0.42855 | 0.025584 | 0.376454 | FAM242F |
| ENSG00000256888.6 | 141.6691 | 0.442489 | 0.025703 | 0.376638 | LINC02366 |
| ENSG00000257900.2 | 68.83292 | 0.433601 | 0.025873 | 0.377926 | AL162632.1 |
| ENSG00000286710.1 | 194.3537 | -0.38369 | 0.026145 | 0.37877 | AC188617.1 |
| ENSG00000205830.1 | 66.05608 | 0.411058 | 0.026243 | 0.379155 | AC024132.1 |
| ENSG00000250954.6 | 624.4057 | 0.381217 | 0.026286 | 0.379155 | AC016687.3 |
| ENSG00000285834.1 | 451.6057 | 0.397436 | 0.026408 | 0.37926 | AL356413.1 |
| ENSG00000234899.11 | 125.2968 | 0.302237 | 0.026661 | 0.380552 | SOX9-AS1 |
| ENSG00000235837.1 | 93.60818 | 0.371652 | 0.026673 | 0.380552 | AC073333.1 |
| ENSG00000223768.2 | 126.9343 | -0.37303 | 0.026734 | 0.380918 | LINC00205 |
| ENSG00000232359.1 | 98.9808 | 0.353733 | 0.026894 | 0.381878 | AC104777.1 |
| ENSG00000248685.6 | 909.9634 | 0.308523 | 0.02699 | 0.382071 | LINC02484 |
| ENSG00000225328.2 | 273.5157 | 0.334181 | 0.027553 | 0.383216 | LINC01594 |
| ENSG00000254300.1 | 448.8824 | 0.345574 | 0.027621 | 0.383216 | LINC01111 |
| ENSG00000226317.3 | 974.8079 | 0.312738 | 0.028199 | 0.384901 | LINC00351 |
| ENSG00000225488.3 | 70.99942 | -0.41387 | 0.028281 | 0.385256 | AC092447.4 |
| ENSG00000267642.1 | 149.7163 | 0.346709 | 0.028538 | 0.386102 | AC091198.1 |
| ENSG00000234172.1 | 197.0751 | 0.430895 | 0.028689 | 0.386102 | AC093639.1 |
| ENSG00000265217.2 | 240.5605 | 0.328572 | 0.028884 | 0.387496 | AC090358.1 |
| ENSG00000262880.1 | 74.24188 | -0.41131 | 0.028997 | 0.387496 | AC113189.2 |
| ENSG00000242440.2 | 438.5595 | 0.344496 | 0.029047 | 0.387496 | LINC02046 |
| ENSG00000242317.1 | 83.87373 | 0.329534 | 0.029328 | 0.388915 | AC121764.1 |
| ENSG00000285079.1 | 70.80294 | 0.39118 | 0.029385 | 0.389349 | AL513493.1 |
| ENSG00000265670.1 | 137.1033 | 0.37579 | 0.029515 | 0.389771 | AC016382.1 |
| ENSG00000257165.5 | 285.7171 | 0.329738 | 0.029722 | 0.390612 | AC079362.1 |
| ENSG00000258867.6 | 388.5223 | 0.323435 | 0.029728 | 0.390612 | LINC01146 |
| ENSG00000226486.2 | 635.249 | 0.393322 | 0.029774 | 0.390612 | LINC01035 |
| ENSG00000250585.3 | 165.372 | 0.34659 | 0.02997 | 0.391875 | LINC00604 |
| ENSG00000287329.1 | 140.2469 | -0.45938 | 0.029989 | 0.391875 | FP326651.1 |
| ENSG00000253613.2 | 60.89255 | 0.383478 | 0.03016 | 0.392057 | AC008572.1 |
| ENSG00000231987.1 | 645.4568 | 0.32121 | 0.030205 | 0.392057 | LINC01787 |
| ENSG00000286679.1 | 666.9598 | 0.373952 | 0.03046 | 0.392057 | AC073225.1 |
| ENSG00000258844.2 | 132.4564 | 0.359084 | 0.030501 | 0.392196 | AL162511.1 |
| ENSG00000257221.4 | 97.64729 | -0.30471 | 0.030525 | 0.392246 | AC007569.1 |
| ENSG00000197251.3 | 59.71059 | -0.41581 | 0.030778 | 0.392623 | LINC00336 |
| ENSG00000223930.7 | 524.9458 | 0.303743 | 0.030853 | 0.392623 | AC117453.1 |
| ENSG00000285778.2 | 695.6715 | 0.318015 | 0.031706 | 0.394563 | AL591463.1 |
| ENSG00000251363.3 | 943.1249 | 0.397812 | 0.031814 | 0.394762 | LINC02315 |
| ENSG00000285718.2 | 735.9991 | 0.394644 | 0.031829 | 0.394762 | AL353072.2 |
| ENSG00000286099.1 | 1359.712 | 0.372917 | 0.031975 | 0.395078 | AL390800.1 |
| ENSG00000287014.1 | 579.8012 | 0.407989 | 0.032068 | 0.395078 | AC087883.2 |
| ENSG00000205628.4 | 825.4791 | 0.305097 | 0.032143 | 0.395078 | LINC01446 |
| ENSG00000288016.1 | 1905.148 | 0.334497 | 0.03219 | 0.395501 | AL162493.1 |
| ENSG00000287578.1 | 453.567 | 0.368109 | 0.032253 | 0.395899 | AL034348.1 |
| ENSG00000287421.1 | 327.2948 | 0.338782 | 0.032434 | 0.396004 | AC131237.1 |
| ENSG00000226780.1 | 61.58309 | 0.382122 | 0.032466 | 0.396004 | AC244035.1 |
| ENSG00000231171.4 | 546.348 | 0.385995 | 0.032589 | 0.396121 | LINC01098 |
| ENSG00000227240.2 | 3661.463 | 0.341263 | 0.032651 | 0.396121 | AL136456.1 |
| ENSG00000273550.2 | 359.4148 | 0.4096 | 0.032916 | 0.396121 | AL354810.1 |
| ENSG00000251361.1 | 242.1344 | 0.404706 | 0.032995 | 0.396121 | AC012625.1 |
| ENSG00000263154.1 | 159.0447 | -0.36617 | 0.033656 | 0.398159 | AC110285.3 |
| ENSG00000224914.4 | 54.46705 | 0.548059 | 0.033762 | 0.398268 | LINC00863 |
| ENSG00000239513.6 | 57.602 | 0.374839 | 0.033849 | 0.398268 | LINC01210 |
| ENSG00000288321.1 | 1148.769 | 0.331965 | 0.033933 | 0.398669 | AC079772.1 |
| ENSG00000267287.1 | 156.6599 | -0.33464 | 0.033961 | 0.398679 | AC068473.3 |
| ENSG00000231019.1 | 517.8914 | 0.328514 | 0.034043 | 0.398797 | LINC00373 |
| ENSG00000224944.2 | 690.734 | 0.317241 | 0.034086 | 0.398999 | CASC6 |
| ENSG00000250320.6 | 543.3324 | 0.340641 | 0.034262 | 0.399469 | EDIL3-DT |
| ENSG00000249787.2 | 156.9865 | 0.352175 | 0.034361 | 0.399728 | AC113385.1 |
| ENSG00000249584.1 | 51.37878 | 0.55508 | 0.034568 | 0.400869 | LINC02225 |
| ENSG00000229797.2 | 111.8379 | -0.32732 | 0.034809 | 0.400869 | AC140481.1 |
| ENSG00000235356.2 | 231.1902 | 0.355305 | 0.03513 | 0.401214 | AL592466.1 |
| ENSG00000167920.10 | 76.66553 | 0.393273 | 0.035346 | 0.402257 | TMEM99 |
| ENSG00000288659.1 | 284.498 | 0.310107 | 0.035742 | 0.403171 | AC007511.1 |
| ENSG00000183822.3 | 157.2854 | -0.35694 | 0.035913 | 0.404247 | NCF4-AS1 |
| ENSG00000278464.2 | 57.61951 | 0.341712 | 0.036169 | 0.405178 | AC068506.1 |
| ENSG00000286675.1 | 221.4882 | 0.341638 | 0.036395 | 0.406233 | AC068700.2 |
| ENSG00000241792.1 | 143.1506 | 0.463937 | 0.036532 | 0.406552 | AC092958.2 |
| ENSG00000287820.1 | 206.0166 | 0.401621 | 0.036674 | 0.406552 | AL050338.2 |
| ENSG00000234435.3 | 147.4376 | 0.310961 | 0.036688 | 0.406552 | LINC01432 |
| ENSG00000280916.2 | 88.4199 | -0.45759 | 0.036708 | 0.406552 | FOXCUT |
| ENSG00000251310.1 | 68.04702 | 0.415543 | 0.03679 | 0.406552 | AC107391.1 |
| ENSG00000225177.6 | 66.81517 | -0.45887 | 0.0369 | 0.406889 | AL590617.2 |
| ENSG00000233256.1 | 80.7317 | -0.4254 | 0.036935 | 0.406889 | AL355870.1 |
| ENSG00000204277.1 | 197.0217 | -0.35379 | 0.03694 | 0.406889 | LINC01993 |
| ENSG00000286543.1 | 126.1214 | 0.417758 | 0.037055 | 0.40734 | AC138409.3 |
| ENSG00000285662.2 | 60.10818 | 0.372747 | 0.037112 | 0.407414 | FAM245B |
| ENSG00000237992.3 | 301.1964 | 0.305417 | 0.037292 | 0.407434 | LINC01808 |
| ENSG00000230040.2 | 291.0528 | 0.325238 | 0.037434 | 0.407434 | LINC00364 |
| ENSG00000268945.1 | 55.1472 | -0.40428 | 0.037664 | 0.407434 | AC010422.2 |
| ENSG00000286260.1 | 286.4261 | 0.317645 | 0.037682 | 0.407434 | AC092604.1 |
| ENSG00000255187.2 | 349.9208 | 0.385793 | 0.038071 | 0.407434 | LINC02748 |
| ENSG00000253182.1 | 112.71 | 0.319975 | 0.038082 | 0.407434 | AC084026.1 |
| ENSG00000250453.2 | 1081.313 | 0.330875 | 0.03816 | 0.407434 | AC008825.1 |
| ENSG00000239774.1 | 211.6565 | 0.319762 | 0.038224 | 0.407434 | AC125618.1 |
| ENSG00000233417.2 | 337.2991 | 0.37049 | 0.038256 | 0.407434 | AC004946.1 |
| ENSG00000229775.7 | 259.7372 | 0.331195 | 0.03857 | 0.408244 | LINC02624 |
| ENSG00000267265.5 | 64.29681 | -0.37513 | 0.038883 | 0.408612 | AC011476.3 |
| ENSG00000256422.6 | 847.6807 | 0.301876 | 0.038957 | 0.408612 | LINC02552 |
| ENSG00000287007.1 | 66.90599 | 0.366993 | 0.039248 | 0.408691 | AC087683.3 |
| ENSG00000251339.6 | 483.3291 | 0.445357 | 0.039304 | 0.408691 | AC017091.1 |
| ENSG00000228627.2 | 84.84621 | 0.412799 | 0.039351 | 0.408691 | AC009468.1 |
| ENSG00000260630.7 | 191.1563 | -0.36823 | 0.039581 | 0.409592 | SNAI3-AS1 |
| ENSG00000285686.1 | 72.13121 | -0.32882 | 0.039655 | 0.409593 | LINC02834 |
| ENSG00000284703.1 | 59.01417 | -0.34048 | 0.039674 | 0.409593 | AL805961.1 |
| ENSG00000240541.3 | 58.50708 | -0.36944 | 0.039802 | 0.409992 | TM4SF1-AS1 |
| ENSG00000286442.1 | 64.85426 | 0.483169 | 0.039877 | 0.410112 | AL512603.1 |
| ENSG00000287941.1 | 187.2598 | 0.323097 | 0.04016 | 0.412118 | AC021055.1 |
| ENSG00000274317.2 | 593.5437 | 0.313303 | 0.040368 | 0.413053 | LINC02334 |
| ENSG00000234556.1 | 57.17188 | 0.368378 | 0.040746 | 0.413534 | LINC00701 |
| ENSG00000257729.2 | 673.9482 | 0.372345 | 0.040812 | 0.413534 | AC090679.2 |
| ENSG00000260852.1 | 52.52104 | -0.50438 | 0.040845 | 0.413534 | FBXL19-AS1 |
| ENSG00000253177.2 | 80.75361 | 0.380506 | 0.041068 | 0.414026 | AC104211.1 |
| ENSG00000250064.1 | 776.2629 | 0.321869 | 0.041598 | 0.415517 | AC097480.1 |
| ENSG00000251049.2 | 58.38361 | 0.402082 | 0.041623 | 0.415517 | AC107396.1 |
| ENSG00000249776.5 | 832.9428 | 0.377135 | 0.042341 | 0.417262 | AC124854.1 |
| ENSG00000284646.1 | 74.61628 | -0.43205 | 0.04267 | 0.417296 | AL031291.1 |
| ENSG00000258532.2 | 61.19628 | 0.417667 | 0.042768 | 0.417296 | LINC02305 |
| ENSG00000183250.12 | 97.72824 | -0.42391 | 0.042774 | 0.417296 | LINC01547 |
| ENSG00000240567.1 | 126.0264 | 0.317509 | 0.042938 | 0.417296 | LINC02067 |
| ENSG00000257488.5 | 55.30225 | -0.41132 | 0.043033 | 0.417354 | LINC02354 |
| ENSG00000224505.2 | 94.45912 | -0.32749 | 0.043291 | 0.417556 | AC138150.1 |
| ENSG00000229766.7 | 140.3944 | -0.31688 | 0.043377 | 0.417998 | AL021396.1 |
| ENSG00000287130.1 | 81.4123 | 0.36651 | 0.043437 | 0.418199 | AC068616.3 |
| ENSG00000178734.5 | 51.84334 | 0.390305 | 0.043529 | 0.418328 | LMO7DN |
| ENSG00000269019.2 | 79.93032 | -0.41281 | 0.043565 | 0.418328 | HOMER3-AS1 |
| ENSG00000198491.3 | 62.31534 | -0.36454 | 0.043919 | 0.418991 | AC007920.1 |
| ENSG00000232307.1 | 109.4014 | 0.448634 | 0.044011 | 0.419137 | DAOA-AS1 |
| ENSG00000286121.1 | 802.7366 | 0.33838 | 0.044222 | 0.419137 | AC093298.2 |
| ENSG00000245910.8 | 53.34844 | -0.34374 | 0.044361 | 0.419137 | SNHG6 |
| ENSG00000257557.2 | 56.96775 | 0.423979 | 0.044374 | 0.419137 | PPP1R12A-AS1 |
| ENSG00000228216.1 | 80.38116 | -0.34672 | 0.044528 | 0.419542 | AL355607.1 |
| ENSG00000253879.2 | 75.90045 | 0.436536 | 0.044673 | 0.419542 | AC087664.2 |
| ENSG00000286563.1 | 135.3439 | 0.325035 | 0.045009 | 0.420047 | AC078983.1 |
| ENSG00000286469.1 | 161.0979 | 0.445851 | 0.045461 | 0.421375 | AL445668.1 |
| ENSG00000226900.2 | 117.4128 | -0.30045 | 0.045575 | 0.421495 | AL451069.1 |
| ENSG00000283828.1 | 68.84354 | -0.41017 | 0.045756 | 0.422082 | AL137002.2 |
| ENSG00000253704.2 | 126.0856 | 0.303331 | 0.045942 | 0.422245 | AC023632.2 |
| ENSG00000234184.6 | 346.8332 | 0.387443 | 0.045959 | 0.422245 | LINC01781 |
| ENSG00000226296.1 | 109.652 | 0.346749 | 0.045977 | 0.422245 | AC036101.1 |
| ENSG00000229424.1 | 109.1761 | -0.31622 | 0.047013 | 0.423926 | AC007349.1 |
| ENSG00000237864.1 | 117.5678 | -0.30847 | 0.047745 | 0.424342 | LINC00322 |
| ENSG00000225539.7 | 1023.844 | 0.403766 | 0.048181 | 0.425473 | LINC01821 |
| ENSG00000235997.3 | 127.223 | -0.31184 | 0.048433 | 0.425986 | LINC01936 |
| ENSG00000224018.2 | 198.1665 | 0.310105 | 0.048518 | 0.426385 | AP000470.1 |
| ENSG00000242268.3 | 121.7916 | 0.386507 | 0.048903 | 0.427158 | LINC02082 |
| ENSG00000285638.1 | 1309.802 | 0.36857 | 0.048992 | 0.42717 | AL138927.1 |
| ENSG00000286539.1 | 128.5327 | 0.355951 | 0.049007 | 0.42717 | AC073973.1 |
| ENSG00000226715.4 | 740.1789 | 0.358792 | 0.049051 | 0.42717 | LINC01709 |
| ENSG00000237498.3 | 317.3758 | 0.318429 | 0.049472 | 0.427989 | AC010105.1 |
| ENSG00000287530.1 | 114.7242 | 0.308239 | 0.049494 | 0.427989 | AL445985.1 |
| ENSG00000255323.6 | 249.0319 | 0.358575 | 0.049504 | 0.427989 | LINC01495 |
| ENSG00000249547.1 | 280.6933 | 0.303181 | 0.049637 | 0.428091 | AC092440.1 |

**Table S11** The list of differentially expressed lncRNAs between grade III and grade I of CRC patients detected in serum sEVs.

| gene_id | baseMean | log2FoldChange | pvalue | padj | gene_name |
| --- | --- | --- | --- | --- | --- |
| ENSG00000251183.1 | 86.52704 | 0.711212 | 8.15E-05 | 0.43135 | LINC01861 |
| ENSG00000254339.5 | 64.11535 | 0.669401 | 0.000198 | 0.587291 | AC064802.1 |
| ENSG00000281333.2 | 111.6421 | 0.783975 | 0.000228 | 0.619946 | AC024941.2 |
| ENSG00000203875.13 | 251.3465 | 0.352765 | 0.000259 | 0.619946 | SNHG5 |
| ENSG00000230014.1 | 66.41013 | 0.741539 | 0.000377 | 0.622986 | LINC00709 |
| ENSG00000205056.8 | 50.09075 | 0.901815 | 0.000537 | 0.622986 | LINC02397 |
| ENSG00000229401.1 | 163.3869 | 0.385596 | 0.000549 | 0.622986 | MIR5689HG |
| ENSG00000231418.2 | 51.52831 | 0.727725 | 0.000671 | 0.622986 | AC005090.1 |
| ENSG00000238131.2 | 103.9213 | 0.702065 | 0.000693 | 0.622986 | LINC02854 |
| ENSG00000286439.1 | 59.4645 | 0.808272 | 0.000795 | 0.622986 | AL354916.1 |
| ENSG00000260910.2 | 66.32979 | -0.64578 | 0.000811 | 0.622986 | LINC00565 |
| ENSG00000283897.2 | 105.4855 | 0.457476 | 0.000857 | 0.622986 | AC011416.3 |
| ENSG00000254380.2 | 289.8013 | 0.3277 | 0.001326 | 0.622986 | AC084734.1 |
| ENSG00000197568.14 | 250.3547 | 0.376776 | 0.001502 | 0.622986 | HHLA3 |
| ENSG00000203565.3 | 161.6323 | 0.479675 | 0.001842 | 0.622986 | AL450313.1 |
| ENSG00000259724.2 | 249.5879 | 0.417671 | 0.001849 | 0.622986 | LINC01581 |
| ENSG00000248801.7 | 179.7064 | 0.367554 | 0.001941 | 0.622986 | C8orf34-AS1 |
| ENSG00000236166.1 | 67.99636 | 0.691123 | 0.002109 | 0.622986 | AL021408.1 |
| ENSG00000225329.3 | 154.501 | 0.348513 | 0.002171 | 0.622986 | LHFPL3-AS2 |
| ENSG00000269966.1 | 81.43844 | 0.57612 | 0.002245 | 0.622986 | AL136164.2 |
| ENSG00000249996.1 | 110.0436 | 0.678532 | 0.002433 | 0.622986 | PPIC-AS1 |
| ENSG00000232058.1 | 264.3901 | 0.318886 | 0.002509 | 0.622986 | AC005772.1 |
| ENSG00000226919.4 | 70.40867 | 0.527204 | 0.002509 | 0.622986 | AL365184.1 |
| ENSG00000223470.3 | 57.13077 | 0.537517 | 0.002683 | 0.635289 | LINC02629 |
| ENSG00000287854.1 | 80.22759 | 0.686285 | 0.002694 | 0.635289 | AC073409.2 |
| ENSG00000235448.3 | 327.5333 | 0.520686 | 0.002923 | 0.645367 | LURAP1L-AS1 |
| ENSG00000286647.1 | 74.89058 | 0.532307 | 0.003148 | 0.645367 | AC008459.1 |
| ENSG00000249171.1 | 359.9578 | 0.38791 | 0.003195 | 0.645367 | AC021192.1 |
| ENSG00000253613.2 | 60.89255 | 0.560283 | 0.003228 | 0.645367 | AC008572.1 |
| ENSG00000286115.1 | 174.1436 | 0.331782 | 0.003301 | 0.645367 | AC010092.1 |
| ENSG00000254862.5 | 75.94186 | 0.508138 | 0.003374 | 0.645832 | LGR4-AS1 |
| ENSG00000272622.2 | 92.33298 | 0.595453 | 0.003756 | 0.662742 | AC010735.2 |
| ENSG00000256128.6 | 164.2418 | 0.489396 | 0.003909 | 0.674238 | LINC00944 |
| ENSG00000229459.1 | 221.1022 | 0.31571 | 0.00398 | 0.674238 | AC023669.1 |
| ENSG00000287605.1 | 60.4852 | 0.65135 | 0.004025 | 0.675009 | AC109131.1 |
| ENSG00000266258.1 | 86.89157 | 0.4264 | 0.004076 | 0.675009 | LINC01909 |
| ENSG00000264260.2 | 89.77337 | -0.42478 | 0.004165 | 0.675009 | LINC01893 |
| ENSG00000261260.2 | 123.2453 | 0.380027 | 0.004192 | 0.675009 | AC106736.1 |
| ENSG00000248837.7 | 1157.475 | 0.366038 | 0.004258 | 0.676197 | AC097512.1 |
| ENSG00000287448.1 | 161.6254 | 0.459583 | 0.004378 | 0.679642 | AC018558.7 |
| ENSG00000285729.1 | 234.4711 | 0.312878 | 0.004489 | 0.679642 | AC103874.1 |
| ENSG00000125899.8 | 198.0014 | 0.446858 | 0.005031 | 0.679642 | LINC02871 |
| ENSG00000227712.2 | 362.202 | 0.317412 | 0.005091 | 0.679642 | AL359915.1 |
| ENSG00000237552.1 | 132.4162 | 0.467097 | 0.005106 | 0.679642 | LINC02567 |
| ENSG00000234540.1 | 80.59104 | 0.585214 | 0.005615 | 0.679642 | AL080313.1 |
| ENSG00000257756.6 | 414.2428 | 0.315497 | 0.005629 | 0.679642 | LINC02386 |
| ENSG00000285804.2 | 125.2883 | 0.414398 | 0.005677 | 0.679642 | AC025774.1 |
| ENSG00000286822.1 | 89.61895 | -0.4049 | 0.005689 | 0.679642 | AC112504.3 |
| ENSG00000238018.2 | 90.48232 | -0.40926 | 0.005946 | 0.679642 | AC093110.1 |
| ENSG00000237735.2 | 290.1835 | 0.499619 | 0.00618 | 0.679642 | AF130359.1 |
| ENSG00000283258.2 | 212.6368 | 0.320245 | 0.006211 | 0.679642 | AC007491.1 |
| ENSG00000242770.2 | 63.86993 | 0.600416 | 0.006271 | 0.679642 | CD200R1L-AS1 |
| ENSG00000240006.1 | 73.04644 | -0.44992 | 0.006272 | 0.679642 | LINC02004 |
| ENSG00000251538.7 | 611.6916 | 0.351996 | 0.006705 | 0.679642 | LINC02201 |
| ENSG00000269929.3 | 78.15981 | 0.547501 | 0.006767 | 0.679642 | MIRLET7A1HG |
| ENSG00000232193.1 | 90.8435 | 0.60813 | 0.006807 | 0.679642 | AL157359.2 |
| ENSG00000228679.1 | 137.27 | 0.425619 | 0.006867 | 0.679642 | AL034347.1 |
| ENSG00000257221.4 | 97.64729 | -0.41328 | 0.006873 | 0.679642 | AC007569.1 |
| ENSG00000233304.7 | 106.5984 | -0.38452 | 0.007041 | 0.679642 | LINC01346 |
| ENSG00000246366.6 | 187.2841 | 0.318624 | 0.007128 | 0.679642 | LACTB2-AS1 |
| ENSG00000231364.3 | 130.782 | 0.509911 | 0.00714 | 0.679642 | LINC01712 |
| ENSG00000236921.1 | 50.82583 | 0.685451 | 0.007231 | 0.679642 | AL157937.1 |
| ENSG00000250436.1 | 142.1294 | 0.421678 | 0.007453 | 0.679933 | LINC02499 |
| ENSG00000244198.7 | 56.95571 | 0.600011 | 0.007546 | 0.679933 | ARHGEF35-AS1 |
| ENSG00000254363.6 | 141.7239 | -0.31264 | 0.007606 | 0.679933 | AC011379.2 |
| ENSG00000285758.2 | 208.7738 | 0.320284 | 0.008051 | 0.681796 | AC036214.4 |
| ENSG00000227075.1 | 212.6732 | 0.452604 | 0.008104 | 0.681796 | AP000472.1 |
| ENSG00000235815.1 | 64.30501 | -0.55817 | 0.008159 | 0.681796 | AL136099.1 |
| ENSG00000280029.4 | 66.62378 | 0.576757 | 0.008471 | 0.69158 | AC244517.11 |
| ENSG00000224851.2 | 181.5695 | 0.381877 | 0.009318 | 0.70776 | LINC00502 |
| ENSG00000255998.2 | 206.1678 | 0.301798 | 0.009391 | 0.70776 | LINC02824 |
| ENSG00000234756.1 | 75.52339 | 0.480871 | 0.009672 | 0.70776 | LINC02621 |
| ENSG00000287364.1 | 71.19015 | 0.74165 | 0.009711 | 0.70776 | AL353709.1 |
| ENSG00000257262.1 | 135.5916 | 0.432652 | 0.01025 | 0.70776 | AC023511.1 |
| ENSG00000223675.1 | 52.60321 | 0.570187 | 0.010271 | 0.70776 | AC093117.1 |
| ENSG00000285556.1 | 183.8577 | 0.343716 | 0.010304 | 0.70776 | AL354897.1 |
| ENSG00000256193.6 | 124.5238 | 0.348563 | 0.010311 | 0.70776 | LINC00507 |
| ENSG00000225488.3 | 70.99942 | -0.52527 | 0.010328 | 0.70776 | AC092447.4 |
| ENSG00000237864.1 | 117.5678 | -0.43301 | 0.01042 | 0.70776 | LINC00322 |
| ENSG00000254334.2 | 66.43236 | 0.608744 | 0.010642 | 0.70776 | AC021355.1 |
| ENSG00000227542.1 | 123.7314 | 0.477119 | 0.010917 | 0.70776 | AC092614.1 |
| ENSG00000268744.1 | 51.76996 | -0.43013 | 0.011203 | 0.70776 | AC008758.4 |
| ENSG00000287372.1 | 180.987 | 0.399424 | 0.011236 | 0.70776 | AL627316.1 |
| ENSG00000248148.1 | 310.58 | 0.524306 | 0.011418 | 0.70776 | AC114954.1 |
| ENSG00000253891.2 | 52.18416 | 0.546408 | 0.011696 | 0.708027 | AC023202.1 |
| ENSG00000260995.3 | 362.6918 | 0.312082 | 0.011709 | 0.708027 | AL512634.1 |
| ENSG00000235837.1 | 93.60818 | 0.455757 | 0.011745 | 0.708027 | AC073333.1 |
| ENSG00000278305.1 | 128.6702 | 0.468122 | 0.011876 | 0.708027 | AL161616.2 |
| ENSG00000180869.4 | 58.39345 | 0.598782 | 0.011972 | 0.708027 | LINC01555 |
| ENSG00000286737.1 | 66.92842 | 0.436139 | 0.012048 | 0.711252 | AC092168.3 |
| ENSG00000258918.1 | 179.5848 | 0.30819 | 0.012064 | 0.711252 | AL355922.4 |
| ENSG00000244650.2 | 140.778 | 0.550713 | 0.012831 | 0.732984 | AC025566.1 |
| ENSG00000267287.1 | 156.6599 | -0.4242 | 0.013155 | 0.736153 | AC068473.3 |
| ENSG00000251055.2 | 70.70783 | 0.603698 | 0.01325 | 0.736153 | AC097491.1 |
| ENSG00000232954.2 | 96.72258 | 0.561655 | 0.013811 | 0.739857 | LINC00374 |
| ENSG00000253949.1 | 157.6271 | 0.35411 | 0.013969 | 0.739979 | AC022634.2 |
| ENSG00000238164.6 | 68.37845 | -0.52518 | 0.01418 | 0.74366 | TNFRSF14-AS1 |
| ENSG00000251562.8 | 382.2992 | -1.50201 | 0.014713 | 0.744387 | MALAT1 |
| ENSG00000286809.1 | 92.92308 | 0.410456 | 0.014899 | 0.744387 | AC008964.1 |
| ENSG00000267772.1 | 192.6888 | -0.32857 | 0.015922 | 0.751705 | LINC01999 |
| ENSG00000248227.1 | 132.6482 | 0.391021 | 0.016049 | 0.751705 | LINC02513 |
| ENSG00000287976.1 | 104.7143 | 0.387787 | 0.016614 | 0.751705 | AL445123.2 |
| ENSG00000198054.12 | 224.3028 | 0.327313 | 0.016699 | 0.751705 | DSCR8 |
| ENSG00000264254.1 | 51.70928 | 0.4786 | 0.016796 | 0.751705 | AP001496.1 |
| ENSG00000253479.6 | 148.4348 | 0.321379 | 0.017309 | 0.751705 | LINC01603 |
| ENSG00000267786.1 | 166.4613 | -0.46136 | 0.017403 | 0.751705 | AF038458.3 |
| ENSG00000287566.1 | 62.79337 | -0.57411 | 0.0175 | 0.751705 | AL732437.3 |
| ENSG00000235079.1 | 91.01235 | 0.390525 | 0.017824 | 0.753668 | ZRANB2-AS1 |
| ENSG00000259230.1 | 139.1275 | -0.35467 | 0.018 | 0.754701 | LINC02323 |
| ENSG00000261475.3 | 68.10242 | -0.40634 | 0.01882 | 0.757955 | LINC02190 |
| ENSG00000229246.2 | 141.0117 | 0.397114 | 0.01897 | 0.757955 | LINC00377 |
| ENSG00000286383.1 | 100.9212 | 0.394723 | 0.019102 | 0.757955 | AC096645.1 |
| ENSG00000224711.2 | 136.1991 | 0.452225 | 0.019267 | 0.757955 | LINC01706 |
| ENSG00000287100.1 | 243.1472 | 0.36091 | 0.019558 | 0.757955 | AL078600.1 |
| ENSG00000232170.6 | 67.58621 | 0.480309 | 0.019567 | 0.757955 | LINC00708 |
| ENSG00000285844.2 | 66.06854 | 0.329881 | 0.019586 | 0.757955 | FO393414.3 |
| ENSG00000251324.1 | 126.3233 | 0.338329 | 0.01992 | 0.757955 | LINC01386 |
| ENSG00000285872.1 | 143.9298 | -0.3331 | 0.020013 | 0.757955 | AC007240.3 |
| ENSG00000226091.7 | 197.4463 | -0.32894 | 0.020275 | 0.75872 | LINC00937 |
| ENSG00000249942.1 | 184.0692 | 0.387011 | 0.020493 | 0.759511 | AC239584.1 |
| ENSG00000287941.1 | 187.2598 | 0.393873 | 0.020623 | 0.759777 | AC021055.1 |
| ENSG00000261817.1 | 58.49153 | 0.521288 | 0.020964 | 0.760171 | AL390718.1 |
| ENSG00000287432.1 | 62.7202 | 0.482666 | 0.021122 | 0.760171 | AC244035.4 |
| ENSG00000260975.1 | 172.0585 | 0.330605 | 0.021411 | 0.760398 | AC007333.2 |
| ENSG00000233654.2 | 230.3347 | -0.32003 | 0.021619 | 0.760398 | AC108047.1 |
| ENSG00000225411.3 | 87.92749 | 0.327304 | 0.021973 | 0.760398 | CR786580.1 |
| ENSG00000266903.2 | 406.8663 | -0.31348 | 0.022045 | 0.760398 | AC243964.3 |
| ENSG00000253206.1 | 94.63109 | 0.508545 | 0.022102 | 0.760398 | AC090155.1 |
| ENSG00000248397.1 | 65.0324 | 0.490682 | 0.022189 | 0.760398 | LINC00498 |
| ENSG00000287980.1 | 126.5441 | 0.42505 | 0.022273 | 0.760398 | AL391557.1 |
| ENSG00000285902.1 | 165.9413 | 0.343793 | 0.022628 | 0.765372 | AL136442.1 |
| ENSG00000235356.2 | 231.1902 | 0.413862 | 0.023232 | 0.766109 | AL592466.1 |
| ENSG00000253738.2 | 62.52917 | 0.530174 | 0.023524 | 0.76637 | OTUD6B-AS1 |
| ENSG00000286421.1 | 53.16181 | 0.621171 | 0.02363 | 0.766764 | AL360013.4 |
| ENSG00000231674.1 | 197.162 | 0.423273 | 0.024136 | 0.768187 | LINC00410 |
| ENSG00000273142.1 | 60.29091 | -0.49984 | 0.024462 | 0.768652 | LINC02604 |
| ENSG00000286211.1 | 188.618 | 0.357544 | 0.024799 | 0.769133 | AC098817.1 |
| ENSG00000286778.1 | 221.0417 | 0.420354 | 0.025544 | 0.769133 | AC013265.1 |
| ENSG00000175967.4 | 131.847 | -0.33325 | 0.025609 | 0.769133 | LINC02880 |
| ENSG00000266950.1 | 101.9811 | -0.3268 | 0.025653 | 0.769133 | AC008752.1 |
| ENSG00000287038.1 | 77.74235 | 0.453107 | 0.025865 | 0.769133 | AL162388.2 |
| ENSG00000255135.4 | 82.73226 | 0.449108 | 0.025969 | 0.769133 | AP002360.1 |
| ENSG00000236769.3 | 66.65479 | -0.40033 | 0.026005 | 0.769133 | LINC02659 |
| ENSG00000229766.7 | 140.3944 | -0.37822 | 0.02612 | 0.769133 | AL021396.1 |
| ENSG00000258586.1 | 72.50213 | -0.42624 | 0.026301 | 0.769133 | LINC02274 |
| ENSG00000239513.6 | 57.602 | 0.422613 | 0.026386 | 0.769133 | LINC01210 |
| ENSG00000253955.2 | 65.25527 | 0.32854 | 0.026454 | 0.769133 | AC008663.2 |
| ENSG00000287499.1 | 95.22457 | 0.518615 | 0.026698 | 0.769133 | AL357139.2 |
| ENSG00000261453.2 | 50.65229 | 0.513387 | 0.026706 | 0.769133 | LINC01735 |
| ENSG00000223811.1 | 64.99191 | 0.456999 | 0.026751 | 0.769232 | AL589684.1 |
| ENSG00000226488.2 | 96.54467 | 0.377618 | 0.026993 | 0.771714 | LINC01824 |
| ENSG00000275216.2 | 61.9784 | 0.379096 | 0.027044 | 0.771714 | AL161431.1 |
| ENSG00000233153.6 | 54.67059 | 0.432302 | 0.027447 | 0.771714 | UBE2E2-AS1 |
| ENSG00000272461.1 | 69.72522 | 0.410502 | 0.027591 | 0.771714 | AP005328.1 |
| ENSG00000255845.1 | 72.95145 | -0.34959 | 0.027704 | 0.771921 | AP000777.1 |
| ENSG00000225177.6 | 66.81517 | -0.52356 | 0.028133 | 0.773796 | AL590617.2 |
| ENSG00000235884.4 | 99.17776 | 0.367604 | 0.028581 | 0.775956 | LINC00941 |
| ENSG00000258844.2 | 132.4564 | 0.391597 | 0.028958 | 0.777831 | AL162511.1 |
| ENSG00000287629.1 | 134.5274 | -0.30396 | 0.029532 | 0.78116 | AC006059.5 |
| ENSG00000238062.6 | 77.57182 | -0.43303 | 0.029675 | 0.78116 | SPATA3-AS1 |
| ENSG00000287048.1 | 75.76512 | 0.536783 | 0.030811 | 0.78168 | AC096589.2 |
| ENSG00000254404.1 | 285.351 | 0.310745 | 0.030887 | 0.78168 | AP003306.1 |
| ENSG00000247373.3 | 108.6794 | -0.36141 | 0.031064 | 0.78168 | TMED2-DT |
| ENSG00000241884.2 | 61.15437 | 0.469133 | 0.031488 | 0.78168 | AC114401.1 |
| ENSG00000256672.1 | 98.03587 | 0.345516 | 0.031945 | 0.78168 | LINC02455 |
| ENSG00000228506.2 | 57.21984 | -0.38364 | 0.032017 | 0.78168 | AL513550.1 |
| ENSG00000287398.1 | 147.6552 | 0.314832 | 0.032064 | 0.78168 | AC117481.1 |
| ENSG00000267506.5 | 138.3212 | -0.31113 | 0.032329 | 0.78168 | AC021683.2 |
| ENSG00000232606.2 | 325.0886 | 0.33999 | 0.032465 | 0.78168 | LINC01412 |
| ENSG00000229109.2 | 108.1138 | -0.37342 | 0.03288 | 0.78168 | AL137847.1 |
| ENSG00000254951.7 | 157.9063 | 0.303915 | 0.032907 | 0.78168 | AC044810.2 |
| ENSG00000234752.1 | 91.85059 | 0.422 | 0.03302 | 0.78168 | LINC02676 |
| ENSG00000237896.6 | 120.8401 | 0.426636 | 0.033867 | 0.78168 | AC005008.2 |
| ENSG00000259989.1 | 162.6911 | -0.40588 | 0.033909 | 0.78168 | AC135782.2 |
| ENSG00000167920.10 | 76.66553 | 0.427708 | 0.033939 | 0.78168 | TMEM99 |
| ENSG00000287989.1 | 385.1417 | 0.329192 | 0.03397 | 0.78168 | AL450352.1 |
| ENSG00000251393.4 | 110.0479 | -0.34488 | 0.034289 | 0.78168 | AC005280.1 |
| ENSG00000259129.6 | 165.7961 | 0.382337 | 0.035121 | 0.785196 | LINC00648 |
| ENSG00000233143.2 | 175.023 | 0.313124 | 0.037242 | 0.794749 | DIRC3-AS1 |
| ENSG00000261864.1 | 77.27002 | -0.48283 | 0.037365 | 0.794749 | AC130462.2 |
| ENSG00000234190.1 | 80.12171 | 0.518122 | 0.037562 | 0.794749 | AC241644.2 |
| ENSG00000286442.1 | 64.85426 | 0.527801 | 0.037573 | 0.794749 | AL512603.1 |
| ENSG00000287804.1 | 92.2599 | -0.41603 | 0.037782 | 0.794749 | Z98745.2 |
| ENSG00000231648.2 | 92.58224 | 0.325154 | 0.038249 | 0.795597 | LINC01698 |
| ENSG00000284699.1 | 140.9211 | -0.3249 | 0.038485 | 0.796413 | AL445648.1 |
| ENSG00000238837.3 | 71.96839 | 0.33795 | 0.03854 | 0.796413 | LINC02031 |
| ENSG00000288062.1 | 54.43856 | -0.46443 | 0.038953 | 0.796413 | AL136981.3 |
| ENSG00000243550.3 | 183.7964 | 0.320754 | 0.039349 | 0.796413 | LINC01214 |
| ENSG00000247311.3 | 89.80031 | 0.461953 | 0.039755 | 0.796413 | AC010255.1 |
| ENSG00000281207.1 | 55.54315 | -0.3906 | 0.039901 | 0.796413 | SLFNL1-AS1 |
| ENSG00000232229.6 | 65.80777 | -0.41855 | 0.04055 | 0.796413 | LINC00865 |
| ENSG00000275356.5 | 86.77948 | 0.521616 | 0.040624 | 0.796413 | C7orf77 |
| ENSG00000284376.1 | 130.5237 | 0.352379 | 0.040663 | 0.796413 | AC092017.3 |
| ENSG00000183822.3 | 157.2854 | -0.37728 | 0.040696 | 0.796413 | NCF4-AS1 |
| ENSG00000248533.1 | 81.42643 | -0.39879 | 0.040724 | 0.796413 | AC034226.1 |
| ENSG00000231231.5 | 92.09681 | -0.34729 | 0.041097 | 0.796413 | LINC01423 |
| ENSG00000288234.1 | 52.83489 | -0.4814 | 0.04121 | 0.796413 | BX255925.4 |
| ENSG00000258028.3 | 798.8836 | 0.325442 | 0.041838 | 0.796413 | AL135878.1 |
| ENSG00000255921.1 | 94.85102 | 0.336056 | 0.042296 | 0.796413 | AC026310.1 |
| ENSG00000265962.1 | 95.7726 | 0.392057 | 0.04237 | 0.796413 | GACAT2 |
| ENSG00000285968.1 | 118.0372 | 0.323574 | 0.042496 | 0.796413 | AL022324.4 |
| ENSG00000286981.1 | 53.73254 | 0.399902 | 0.042691 | 0.796413 | AL731553.1 |
| ENSG00000287910.1 | 80.84912 | -0.32474 | 0.043102 | 0.796413 | AC104024.4 |
| ENSG00000163597.15 | 58.86931 | -0.41039 | 0.043178 | 0.796413 | SNHG16 |
| ENSG00000253636.1 | 82.72888 | 0.317963 | 0.04322 | 0.796413 | AC022893.1 |
| ENSG00000238265.1 | 73.79212 | 0.492522 | 0.043532 | 0.796413 | LINC00317 |
| ENSG00000239628.1 | 72.5546 | -0.38804 | 0.043685 | 0.796413 | AC073288.1 |
| ENSG00000267686.2 | 205.7475 | 0.326159 | 0.043709 | 0.796413 | AC090771.2 |
| ENSG00000224467.1 | 256.402 | 0.331748 | 0.043763 | 0.796413 | TANK-AS1 |
| ENSG00000258175.1 | 108.9367 | 0.469814 | 0.044103 | 0.796413 | LINC02300 |
| ENSG00000232458.1 | 114.9567 | 0.308263 | 0.044118 | 0.796413 | LINC01450 |
| ENSG00000269994.3 | 199.6793 | 0.310232 | 0.044379 | 0.796413 | AL513318.1 |
| ENSG00000253871.2 | 198.9535 | 0.306721 | 0.044681 | 0.796625 | AC068075.1 |
| ENSG00000186235.11 | 99.16497 | -0.36794 | 0.044744 | 0.796724 | LINC02610 |
| ENSG00000258386.1 | 94.02278 | 0.315382 | 0.044988 | 0.797063 | AL352984.1 |
| ENSG00000253287.1 | 68.2513 | 0.46184 | 0.045679 | 0.798207 | AC104012.1 |
| ENSG00000287480.1 | 73.29949 | -0.36646 | 0.045738 | 0.798207 | AL158832.2 |
| ENSG00000263154.1 | 159.0447 | -0.37242 | 0.046136 | 0.800503 | AC110285.3 |
| ENSG00000227432.1 | 89.6168 | -0.40573 | 0.046573 | 0.80165 | ASIC4-AS1 |
| ENSG00000245910.8 | 53.34844 | -0.36894 | 0.046644 | 0.80165 | SNHG6 |
| ENSG00000249451.1 | 56.46038 | 0.449192 | 0.047566 | 0.803325 | AC021491.1 |
| ENSG00000258414.1 | 69.29947 | 0.425339 | 0.04787 | 0.803333 | AL121790.1 |
| ENSG00000223768.2 | 126.9343 | -0.36074 | 0.047988 | 0.803333 | LINC00205 |
| ENSG00000232298.2 | 109.0756 | -0.31869 | 0.048675 | 0.80372 | GRHL3-AS1 |

**Table S12** The list of differentially expressed lncRNAs between grade III and grade II of CRC patients detected in serum sEVs.

| gene_id | baseMean | log2FoldChange | pvalue | padj | gene_name |
| --- | --- | --- | --- | --- | --- |
| ENSG00000280441.3 | 258.6878 | -1.29048 | 0.00010578 | 0.999895 | FP236383.3 |
| ENSG00000258107.3 | 139.8596 | -0.37597 | 0.00067194 | 0.999895 | AL158058.1 |
| ENSG00000232130.1 | 139.6866 | -0.36095 | 0.00124838 | 0.999895 | AC092966.1 |
| ENSG00000284601.1 | 65.4542 | 0.371697 | 0.00172958 | 0.999895 | AL590440.2 |
| ENSG00000288537.1 | 53.95056 | 0.343369 | 0.00307433 | 0.999895 | AL162391.2 |
| ENSG00000251183.1 | 86.52704 | 0.319015 | 0.00457351 | 0.999895 | LINC01861 |
| ENSG00000261453.2 | 50.65229 | 0.405582 | 0.00540471 | 0.999895 | LINC01735 |
| ENSG00000275239.4 | 99.5171 | 0.368166 | 0.00550022 | 0.999895 | FAM242F |
| ENSG00000203434.2 | 75.12987 | -0.43124 | 0.00571811 | 0.999895 | AL353740.1 |
| ENSG00000237494.1 | 61.5424 | -0.3391 | 0.00719587 | 0.999895 | AL360007.1 |
| ENSG00000231418.2 | 51.52831 | 0.355311 | 0.00747557 | 0.999895 | AC005090.1 |
| ENSG00000286820.1 | 61.23413 | -0.4306 | 0.01050931 | 0.999895 | AL590027.1 |
| ENSG00000248456.1 | 58.54449 | -0.36221 | 0.01393836 | 0.999895 | LINC02485 |
| ENSG00000258616.5 | 61.07581 | -0.36874 | 0.01413019 | 0.999895 | LINC02303 |
| ENSG00000241792.1 | 143.1506 | -0.37027 | 0.01512801 | 0.999895 | AC092958.2 |
| ENSG00000246640.1 | 56.28893 | 0.348723 | 0.01594009 | 0.999895 | PICART1 |
| ENSG00000225793.3 | 58.17791 | -0.32424 | 0.02011286 | 0.999895 | TMEM30A-DT |
| ENSG00000224028.1 | 162.7663 | -0.31092 | 0.02216366 | 0.999895 | LINC01853 |
| ENSG00000232164.1 | 50.56819 | -0.36234 | 0.02338964 | 0.999895 | LINC01873 |
| ENSG00000258636.2 | 107.6928 | -0.32272 | 0.02519668 | 0.999895 | AL121821.2 |
| ENSG00000273956.1 | 84.87782 | 0.300756 | 0.02581259 | 0.999895 | AC005632.5 |
| ENSG00000235139.2 | 245.0164 | -0.30441 | 0.02675449 | 0.999895 | AC003984.1 |
| ENSG00000227902.2 | 88.48223 | -0.31403 | 0.02680246 | 0.999895 | AC062032.1 |
| ENSG00000263863.1 | 95.13611 | -0.3217 | 0.02851105 | 0.999895 | AC007948.1 |
| ENSG00000287453.1 | 58.97156 | -0.38205 | 0.03555343 | 0.999895 | AL359894.1 |
| ENSG00000255362.1 | 61.79324 | 0.31739 | 0.03711946 | 0.999895 | LINC02761 |
| ENSG00000244278.1 | 71.06749 | -0.31077 | 0.03928251 | 0.999895 | AP000235.1 |
| ENSG00000248330.5 | 208.7997 | -0.31018 | 0.0393581 | 0.999895 | LINC00613 |
| ENSG00000241505.1 | 65.61621 | -0.36048 | 0.04252063 | 0.999895 | AL391645.1 |
| ENSG00000227716.1 | 59.80907 | -0.33627 | 0.04535733 | 0.999895 | AP000459.1 |
